# The Nrf2 Pathway in Depressive Disorders: A Systematic Review of Animal and Human Studies

**DOI:** 10.3390/antiox12040817

**Published:** 2023-03-27

**Authors:** Gabriele Sani, Stella Margoni, Andrea Brugnami, Ottavia Marianna Ferrara, Evelina Bernardi, Alessio Simonetti, Laura Monti, Marianna Mazza, Delfina Janiri, Lorenzo Moccia, Georgios D. Kotzalidis, Daniela Pia Rosaria Chieffo, Luigi Janiri

**Affiliations:** 1 Institute of Psychiatry, Department of Neuroscience, Catholic University of the Sacred Hearth, Rome, Largo Francesco Vito 1, 00168 Rome, Italy; 2 Department of Psychiatry, Department of Neuroscience, Head, Neck and Thorax, Fondazione Policlinico Universitario Agostino Gemelli IRCCS, Largo Agostino Gemelli 1, 00168 Rome, Italy; 3 Menninger Department of Psychiatry and Behavioral Sciences, Baylor College of Medicine, 1 Baylor Plaza, Houston, TX 77030, USA; 4 Centro Lucio Bini, Via Crescenzio 42, 00193 Rome, Italy; 5 UOS Clinical Psychology, Clinical Government, Fondazione Policlinico Universitario Agostino Gemelli IRCCS, Largo Agostino Gemelli 1, 00168 Rome, Italy; 6 NESMOS Department, Faculty of Medicine and Psychology, Sant’Andrea University Hospital, University of Rome La Sapienza, Via di Grottarossa, 1035-1039, 00189 Rome, Italy

**Keywords:** depression, Nuclear factor erythroid-2 (Nrf2), pathophysiology, antioxidant pathways, Haemoxygenase (HO-1), Nuclear factor kappa B (NF-κB)

## Abstract

There is increasing interest in the involvement of antioxidative systems in protecting from depression. Among these, Nrf2 occupies a central place. We aimed to review the role of Nrf2 in depression. For this reason, we conducted a PubMed search using as search strategy (psychiatr*[ti] OR schizo*[ti] OR psychot*[ti] OR psychos*[ti] OR depress*[ti] OR MDD[ti] OR BD[ti] OR bipolar[ti] OR Anxiety[ti] OR antidepress*[ti] OR panic[ti] OR obsess*[ti] OR compulsio*[ti] OR “mood disord*”[ti] OR phobi*[ti] OR agoraphob*[ti] OR anorex*[ti] OR anorect*[ti] OR bulimi*[ti] OR “eating disorder*”[ti] OR neurodevelopm*[ti] OR retardation[ti] OR autism[ti] OR autistic[ti] OR ASM[ti] OR adhd[ti] OR “attention-deficit”[ti]) AND nrf2, which on the 9th of March produced 208 results of which 89 were eligible for our purposes. Eligible articles were studies reporting data of Nrf2 manipulations or content by any treatment in human patients or animals with any animal model of depression. Most studies were on mice only (N = 58), 20 on rats only, and three on both rats and mice. There were two studies on cell lines (*in vitro*) and one each on nematodes and fish. Only four studies were conducted in humans, one of which was *post mortem*. Most studies were conducted on male animals; however, human studies were carried out on both men and women. The results indicate that Nrf2 is lower in depression and that antidepressant methods (drugs or other methods) increase it. Antioxidant systems and plasticity-promoting molecules, such as those in the Nrf2–HO-1, BDNF–TrkB, and cyclic AMP–CREB pathways, could protect from depression, while glycogen synthase kinase-3β and nuclear factor κB oppose these actions, thus increasing depressive-like behaviours. Since Nrf2 is also endowed with tumorigenic and atherogenic potential, the balance between benefits and harms must be taken into account in designing novel drugs aiming at increasing the intracellular content of Nrf2.

## 1. Introduction

Recently, much research has been devoted to the study of inflammation and its role in the context of a wide variety of pathological conditions. Inflammation and oxidative stress were found to enhance each other, thus establishing a pathological state [1], which may be found in various psychiatric disorders. The variety of external stimuli to which organisms are subjected triggers adaptive responses, which are designed to restore homoeostasis through a fine balance between oxidation and antioxidant activity [2].

An imbalance between the generation of reactive oxygen species (ROS) and antioxidant defences results in increased oxidative stress [3], with a consequent increase in neuroinflammation, mitochondrial dysfunction, and cell degeneration processes such as apoptosis and ferroptosis, which proved to be crucial in many psychiatric disorders. This is not surprising if we consider that the brain consumes massive doses of oxygen and contains high concentrations of oxidative lipids, thus being extremely vulnerable to oxidative stress-induced damage [2].

In this framework, it is important to draw attention to nuclear factor erythroid-2 (Nrf2), the main endogenous negative regulator of oxidation [4]. Its activation determines the expression of numerous antioxidants and cytoprotective genes capable of modulating oxidative stress. Nrf2 also presides over the regulation of genes involved in the oxidative stress-related pathological processes mentioned above. Consequently, dysregulation in Nrf2 expression with reduced cortical levels may contribute to the aetiopathogenesis of numerous pathological conditions, including psychiatric disorders and neurodegenerative diseases.

Nrf2 is a protein transcription factor composed of 605 amino acids, encoded by the *NFE2L2* gene and belonging to the Cap’n’collar (CNC) family of transcription factors [5]. It contains seven highly conserved functional domains called Nrf2-ECH homology 1 (Neh1-Neh7). Neh1 and Neh3 interact with specific DNA sequences called antioxidant response elements (ARE) [6], thus promoting the transcription of enzymes with antioxidant activity. Neh2 interacts with Kelch-like ECH-associated protein 1 (Keap1), the main negative regulator of Nrf2. Neh4 and Neh5 interact with the cyclic adenosine monophosphate (cAMP)-response element-binding protein (CREB), which also promotes transcriptional activation [7]. In contrast, Neh6 can link to β-transducin repeat-containing protein (β-TrCP) and is involved in Keap1-independent degradation of Nrf2. Finally, Neh7 inhibits the Nrf2-ARE signalling pathway by binding to retinoic X receptor alpha (RXRα) [8]. Therefore, the activity of Nrf2 is subjected to both Keap1-dependent and Keap1-independent regulation.

Keap 1 represents the main Nrf2 suppressor; it forms a homodimer capable of binding ETGE and DLG motifs (stronger and weaker binding sites of Keap-1, respectively; the former is located in the loop region of the antiparallel β-sheet, while the latter is N-terminal to the α-helix [9]) included in the Neh2 domain of Nrf2. Under physiological conditions, the Keap1-Nrf2 complex combines with the E3-ubiquitin ligase Cullin 3 (Cul3) complex, leading to ubiquitination and proteasomal degradation of Nrf2 [10]. Negative regulation is also mediated by the phosphorylation of Nrf2 by glycogen synthase kinase-3 beta (GSK-3β) and mitogen-activating protein kinase (MAPK) [11].

When oxidative stress increases and ROS accumulation occurs, there is a dissociation of the Keap1-Nrf2 complex induced by conformational changes of a Keap1 domain, inhibiting ubiquitination and subsequent degradation of Nrf2. Dissociated from Keap1, Nrf2 is free to translocate into the nucleus and bind specific genomic sequences in order to promote antioxidant enzyme transcription. At the same time, there is a positive Keap1-independent regulation mediated by other kinases. The kinases involved in the phosphorylation and subsequent activation of Nrf2 include protein kinase C (PKC), casein kinase II (CK2), protein kinase R (PKR), c-Jun N-terminal kinase (JNK), and extracellular regulated kinases (ERKs) [12,13,14,15]. Finally, brain-derived neurotrophic factor (BDNF) can also promote the activation and subsequent migration of Nrf2 into the nucleus [16].

In addition to its antioxidant activity, Nrf2 is also directly involved in oxidative stress-related pathological processes by regulating their activation. In particular, there is a direct cross-talk between Nrf2 and p62, an autophagy key protein. Nrf2 can promote the expression of genes involved in autophagy, while p62 can compete with Nrf2 in binding to Keap1 in a positive feedback that is associated with cytoprotection [6,17]. In addition, Nrf2 is also involved in a special form of autophagy called mitophagy, the alteration of which allegedly plays an important role in psychiatric disorders. While it preserves mitochondrial integrity, mitophagy entails the elimination of damaged or redundant mitochondria through autophagy [18].

Furthermore, recent studies of Nrf2 found that its inducers promote the suppression of the pro-inflammatory phenotype of microglia through regulating BDNF, the reduction of which is found in many psychiatric disorders characterised by neuroinflammation [16,19,20,21]. The existence of reciprocal regulation has emerged between BDNF and Nrf2; the latter, in connecting with the exon I promoter of *bdnf*, can activate BDNF; on the other side, BDNF can increase the nuclear translocation of Nrf2, thus promoting its antioxidant activity [16].

Finally, several studies also cast light on the involvement of Nrf2 in ferroptosis, an iron-dependent form of programmed cell death characterised by the accumulation of lipid peroxides (lipids damaged by oxidation). Notably, Nrf2 does not only regulate numerous genes involved in iron metabolism and homoeostasis but also promotes the basal expression of the lipid hydroperoxidase, glutathione peroxidase-4 (GPX4), which converts toxic lipid peroxides to nontoxic lipid alcohols [22].

Impaired response to oxidative stress has been shown in animal models for stress disorders, such as post-traumatic stress disorder (PTSD) [23,24,25,26], but only a few studies have focused on Nrf2 [27,28,29]. PTSD and depression are long considered to represent stress disorders and share common neurobiological patterns [30]. Recently, the Nrf2-depression connection has received attention [2]. We decided to search the literature for studies investigating the ties between depression paradigms in the animal and/or major depressive disorder in humans and Nrf2 as a proxy of a mechanism that counters oxidative stress. Establishing such a relationship would allow us to concentrate on the production of drugs that would promote the search for drugs interfering with intracellular oxidative processes.

## 2. Methods

To systematically review the ties between depression and Nrf2, we first conducted a PubMed search involving all possible mental and psychiatric disorders and then focused on depression. The inclusion of other than depressive disorders/states was to make sure that depression during the course of other mental disorders was not dealt with. We employed the following search strategy: (psychiatr*[ti] OR schizo*[ti] OR psychot*[ti] OR psychos*[ti] OR depress*[ti] OR MDD[ti] OR BD[ti] OR bipolar[ti] OR Anxiety[ti] OR antidepress*[ti] OR panic[ti] OR obsess*[ti] OR compulsio*[ti] OR “mood disord*”[ti] OR phobi*[ti] OR agoraphob*[ti] OR anorex*[ti] OR anorect*[ti] OR bulimi*[ti] OR “eating disorder*”[ti] OR neurodevelopm*[ti] OR retardation[ti] OR autism[ti] OR autistic[ti] OR ASM[ti] OR adhd[ti] OR “attention-deficit”[ti]) AND nrf2. The choice of the search strategy was based on consultations among all authors.

**Figure 1:**
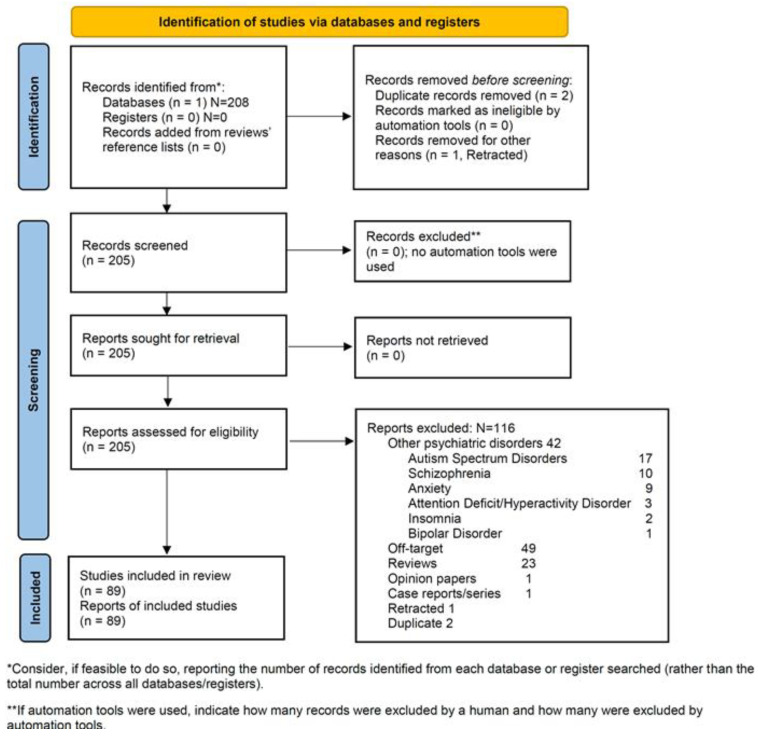
*PRISMA* 2020 flow diagramme (From [31]: Page, M.J.; McKenzie, J.E.; Bossuyt, P.M.; Boutron, I.; Hoffmann, T.C.; Mulrow, C.D.; Shamseer, L.; Tetzlaff, J.M.; Akl, E.A.; Brennan, S.E.; et al. The PRISMA 2020 statement: An updated guideline for reporting systematic reviews. *BMJ* **2021**, *372*, n71. https://doi.org/10.1136/bmj.n71. For more information, visit: http://www.prisma-statement.org/ accessed on 12 February 2023) of the review.

Following search performance, we characterised the nature of all ensuing records and labelled them accordingly. This resulted in their being either included or excluded. The inclusion/exclusion labelling with the reasons for exclusion is shown in the Online Supplemental material and in Figure 1, where the PRISMA flow diagram is displayed. In carrying out our review, we followed the 2020 Preferred Reporting Items for Systematic reviews and Meta-Analyses (PRISMA) statement [31]. The 2020 PRISMA Checklist may be found in the Online Supplement. We assessed the Risk of bias (RoB) of the included studies with the Cochrane RoB 2.0 tool [32]. We performed an evaluation of the RoB for each included study. The results are shown in the Online Supplement.

Eligibility was based on being an original study on any animal or tissue, including humans, on investigating depression or depression models and providing data on Nrf2 levels. All other studies were excluded. Excluded were case reports, opinion articles, such as editorials, letters to the editor, comments of other work, reviews, and meta-analyses (however, we hand-searched their reference lists to identify other possibly eligible studies), and studies not providing data. Eligibility for each paper was established with the consensus of all authors obtained through Delphi rounds, in which all authors participated, either in-person or online. The same applied to the compilation of the RoB.

## 3. Results

Our search, eventually conducted on the 10th of March 2023, yielded 208 results on PubMed, of which 89 studies were eligible, as summarised in Table 1 (human studies, of which two used cell lines *in vitro*) and Table 2 (animal studies). In particular, there were 78 articles labelled Depression and 11 Depression and Anxiety; these amounted to 89 articles. The remaining 116 studies were excluded. Depression-free articles focused on autism (N = 17), schizophrenia (N = 10), anxiety (N = 9), attention-deficit/hyperactivity disorder (N = 3), insomnia (N = 2), and bipolar disorder alone (N = 1). Other articles that did not meet the inclusion criteria were Opinions (N = 1), Case reports (N = 1), and Reviews (N = 23), while many articles were off-target (N = 49) in that they did not focus any psychiatric disorder and were unfocused in their designs or they were unrelated to the subject of our inquiry. Furthermore, there were two duplicates and a retracted paper, but another paper from the same group had not been retracted and dealt with the same issue as the retracted one. Publication dates spanned from 23-March-2006 to 1-March-2023 for the searched papers and 23-April-2013 to 1-March-2023 for the eligible ones. The included and excluded studies with their reasons for exclusion are shown in the Supplement. The selection process and reasons for exclusion are depicted in the PRISMA flowchart (Figure 1).

Of the 89 studies included in this review, most were conducted on mice only (N = 58; 65.17%), 20 were conducted on rats only (22.47%), and three on both rats and mice (3.37%), four on humans (4.49%), two on human cell lines in vitro (2.25%), one on fish and worms each (1.12%). Of the 58 studies carried out on mice only, 50 used only male animals, seven used only female, and one both used animals of both sexes; 31 used C57BL/6 strains; one reported unspecified C57 mice (which were presumably C57BL/6, based on other articles by the same group of authors), 10 used unspecified Swiss strains, five used CD-1, eight used Balb/c, five ICR, four Kunming, one BXD Recombinant Inbred, and one Murphy Roths Large lymphoproliferative Mouse (MRL/lpr); in eight of these studies, investigators used more than one strain. All 20 studies conducted on rats used only male animals, 14 Sprague Dawley and six Wistar, of which two were Hannover and two albino Wistar, while all three studies that employed both mice and rats were conducted using male-only animals, all three used Sprague Dawley rats (1 also Groningen, a strain characterised by high aggression levels [33]) and C57BL/6 mice. The four human studies included patients and matched controls of both sexes, while the only *post mortem* study did not report the sex or the age of included patients. The study that used fish employed the Japanese rice fish, medaka (*Oryzias latipes*), both male and female, and the one that used nematodes used *Caenorhabditis elegans*, while of the two conducted on cell lines, one used the macrophage RAW26.7 line (primary CD14+ monocytes from human donors of both sexes transformed in macrophages through 1-week Colony Stimulation Factor-1 stimulation) and the other used human neuroblastoma SH-SY5Y cells. Of the 85 studies that specified the sex of the animals they used in their experiments, 79 used males (92.94%) and only 12 used females (14.12%). Limiting the sex of animals to the 81 rodent studies, which constituted the bulk of eligible studies included in this review, it results that 73 studies employed male-only animals (90.12%), while only seven studies (9.59%) used female-only animals. This shows a strong bias toward the use of male animals in depression studies of rodent models that cannot be easily translated to humans, given that the majority of people with depression are women [34,35] or female adolescents [36].

Of the eligible studies, most were conducted in China (N = 49, 55.06%; only three were located in Beijing, China’s capital, while six studies were conducted in the Guangdong Province, i.e., three in Shenzhen, two in Guangzhou, and one in Zhanjiang), eight in Brazil (8.99%) and seven in Spain, of which one was a multinational study shared with other four countries (6.97%), five in Japan (5.62%), four in Egypt (4.49%), three in the US (3.37%), two in South Korea and France (2.25% each), two in Germany and Poland, with one multinational shared (1.35% each), one each in India, Iran, Italy, Nigeria, Pakistan, Serbia, and Turkey (1.12% each), and one in both Romania and Sweden, sharing the same multinational study as the other three (0.22% each). Of the 89 included studies, 15 did not use any specific drug to identify its effects on Nrf2 but rather focused on the effects of specific animal depression models on the entire antioxidant system. These 15 studies were conducted in China (N = 8), Spain (N = 3), Serbia, the USA, Brazil, and South Korea (N = 1 each). Plant extracts or animal tissue extracts were tested in 29 Chinese studies, three Japanese studies, one Pakistani, one Nigerian, and one South Korean study, for a total of 35 studies, representing 39.33% of all included studies, with China accounting for 82.86% of these studies and plant extract using studies for 59.18% of all Chinese studies.

**Table 1:** Summary of human studies investigating the role of Nrf2 in depression (*in vivo*, *post mortem*, in vitro on cell lines).

Study	Animal	Paradigm/Model	Location	Design	Results Relating to Nrf2	Conclusions/Observations
Lukic et al., 2014 [37]	Man; 30 patients with MDD (17 ♁; 13 ♂; Ham-D > 14; $\bar{x}$ age 44.77 ± 7.58 yr) vs. 35 HC (19 ♁; 16 ♂; $\bar{x}$ age 39.49 ± 9.64 yr, younger, *p* = 0.018)	OS	Belgrade, Serbia	PBMC investigated with WB for Nrf2, Keap1, NF-κB, AOEs (MnSOD, CuZnSOD, GPx, GLR) in MDD patients and HC	↑ Nrf2, Keap1, NF-κB, MnSOD, CuZnSOD, and CAT in MDD vs. HC in PBMC cytoplasm; ≈GPx and GLR between MDD and HC, but ↓GLR/GPx in MDD; MnSOD, CuZnSOD, and CAT levels correlated directly with Nrf2 levels, while MnSOD and CuZnSOD correlated with NF-κB levels	Impaired oxidative detoxification capacity in MDD, ↓ capacity of GPx to defend from OS in PBMC of MDD patients; the up-regulation of Nrf2 and NF-κB and their down-stream targets MnSOD, CuZnSOD, and CAT indicate OS status in PBMCs of MDD patients
Mellon et al., 2016 [38]	Man; 20 unmedicated MDD patients (♂, ♁) + 20 HC	MDD human model	San Francisco, CA, USA	I group = 20 unmedicated MDD subjects; II group = 20 age-, sex- and ethnicity-matched HC, before initiation of AD treatment, and in 17/20 of the unmedicated MDD subjects after 8 wk of sertraline treatment→ transcriptome-driven bioinformatic strategy to evaluate the activity of several transcriptional Ctrl pathways	In leukocytes from unmedicated MDD subjects ↑ transcriptional activity of cAMP response element-binding/activating TF (CREB/ATF) and Nrf2. 8 wk sertraline treatment was associated with ↓ in Ham-D scores and ↓ activity of Nrf2, but not in CREB/ATF activity. Several other transcriptional regulation pathways, including the glucocorticoid receptor, NF-κB and (EGR1–4) and interferon-responsive TFs, showed either no significant differences as a function of disease or treatment	CREB/ATF and Nrf2 signalling may contribute to MDD by activating immune cell transcriptome dynamics that ultimately influence central nervous system (CNS) motivational and affective processes via circulating mediators
Martín-Hernández et al., 2018 [39]	*Post-mortem* dlPFC samples of 30 Caucasian pts with MDD, ethnic origin-, gender-, and age-matched to 30 HC (sex not specified, although it was said that men were more, age of death not declared)	NI and OS in MDD *post mortem*	Bilbo, Bizcaia, Euskal Herria, Spain	2 MDD groups: AD-free (N = 15) and AD-treated (N = 15). WB for levels of TLR-4, Hsp60, Hsp70, p-ERK 1/2, p-JNK, p-p38, p38 α/β, I3K, Keap-1, p11,DUSP-2, Nrf-2, NF-κB p65 subunit in cytosol and nucleus of dlPFC neurones	↓ Nrf2 pathway in pts with MDD. AD treatments do not reverse the trend	↑ ERK 1/2 (+22%, *t* = 2.293, *p* = 0.03) and JNK (+56%, *t* = 2.468, *p* = 0.02) expression in MDD pts, but not p38-MAPK, compared to HC. p-JNK/total JNK and p-p38/total p38 ↑ in MDD > HC. AD-free and AD-treated showed no significant ↑ in Keap-1 expression compared to HC. 21% ↓ of nuclear expression of Nrf2 in MDD pts
Kubick et al., 2020 [40]	*In vitro* cells (macrophage cell line RAW26.7 from 3 ♁ and 4 ♂ human donors, treated with LPS)	Drug repurposing, LPS-induced OS	Hamburg, Germany; Madrid, Spain; Bucharest, Romania; Stockholm, Sweden; Garbatka, Poland	RNA-seq Data Analysis, AI workflow (which drugs activate NRF2?), in vitro cells treated with ZT, Protein Assay (anti-NRF2), Chemiluminescence	RAW264.7 cells treated with ZT (10 μM, 16 h) showed ↑NRF2 levels compared to plac-treated Ctrl cells	Nrf2 pathway is a putative regulator of M1 function in depression; Nrf2 is a potential drug target; ZT activates Nrf2 and its downstream targets
Goetzl et al., 2021 [41]	Man; 10 MDD Resp patients (6 ♁; 4 ♂; Ham-D > 14; $\bar{x}$ age 39.0 ± 9.4 yr) 10 MDD NResp patients (6 ♁; 4 ♂; Ham-D > 23; $\bar{x}$ age 41.3 ± 11.6 yr) vs. 10 HC (5 ♁; 5 ♂; $\bar{x}$ age 37.5 ± 10.5 yr)	OS	San Francisco, CA; New Haven, CT, USA	Two groups: MDD Resp → sertraline or escitalopram or fluoxetine × 8 wk at sertraline-equivalent doses; MDD NResp → sertaline or escitalopram, fluoxetine or citalopram at sertraline-equivalent doses; ELISA for Nrf2 and MCh proteins	NDEV levels of NRF2 were statistically ↓ in the NResp at BL and Resp at BL groups than in their Ctrl groups; levels ↑ in the NResp and Resp groups after treatment	NDEV levels of MPs of all functional classes, except complex I-6, NRF2 and PGC-1α, were normalised in MDD participants who responded to SSRI treatment but not in those who failed to respond, as assessed by the psychiatrist; the sample was small
X. Li et al., 2022 [42]	Human neuroblastoma SH-SY5Y cells	H_2_O_2_ induced SH-SY5Y cell damage	Beijing, China	Human neuroblastoma SH-SY5Y cells used to mimic OS damage *in vitro*. Four groups: untreated Ctrl, H_2_O_2_-induced injury model, kaempferol treatment, and ginsenoside rh2 treatment. WB to detect Nrf2, Trx, and Akt1. TrxR activity was Measur with the Solarbio thioredoxin reductase activity Kit	Kaempferol and ginsenoside rh2 ↑ the expression of Akt1 and Nrf2, which boosted the targets in the Akt1/Nrf2/Trx pathways cascade working conjointly. Kaempferol works better than ginsenoside rh2 in the Akt1/Nrf2/Trx pathways	Kaempferol and Rh2 could enhance the activity of the Trx system by up-regulating Akt1 to activate Nrf2 *in vitro*

For *abbreviations*, see note to Table 2.

**Table 2:** Summary of animal studies investigating the role of Nrf2 in depression.

Study	Animal	Paradigm/Model	Location	Design	Results Relating to Nrf2	Conclusions/Observations
Martín-de-Saavedra et al., 2013 [43]	♂ WT C517BL/6 mice (Nrf2^+/+^) and Nrf2 KO (Nrf2^−/−^) and ♂ Swiss mice (3–4-month-old)	LPS-induced DLB	Madrid, Spain	1. WT (Nrf2^++^) and KO (Nrf2^--^) mice were subjected to behav tests (TST, OFT, SPlT)+ biochemical analysis; WT mice received LPS (0.1 mg/kg via IP)+ SFN (1 mg/kg/day via ip for 7 days); 3. KO mice + Rofecoxib (2 mg/kg/day for 7 days)	Nrf2 deletion resulted in DLB (↑ in the immobility time in the TST and by a ↓ in the grooming time in the SPlT); ↓ of Dopa and Ser and ↑ Glu in the PFC;↑ of VEGF and synaptophysin;↑microgliosis. Nrf2 KO mice treatment with rofecoxib reversed their DLB; SFN in LPS-induced depression of WT mice afforded AD-like effects	Inflammation due to a deletion of Nrf2 can lead to a depressive-like phenotype, while the induction of Nrf2 could become a new and interesting target for developing novel AD drugs
Mendez-David et al., 2015 [44]	Adult ♂ C57BL/6Ntac mice, Nrf2 WT ^(+/+)^ and knock-out Nrf2^(−/−)^	Mouse CORT model of DLB	Paris, France	Chronic corticosteroids, chronic fluoxetine 4 wk later; OFT, E + M, NSFT, and SPIT testing during the wk following chronic fluoxetine. Immunoblotting for BDNF, Nrf2, and its downstream targets GCLC, NQO1, and HO-1, in cortical and Hippoc membranes	Chronic fluoxetine restored Nrf2 levels in mouse cortex, as well as GCLC, HO-1, and NQO1 levels that were ↓ by chronic CORT. In the Hippoc, Nrf2 was not ↓, but GCLC, HO-1, and NQO1 levels were ↓; chronic fluoxetine restored GCLC and NQO1 levels, but not HO-1. Chronic fluoxetine ↑ cortical BDNF levels and reversed CORT-induced ↓ in Hippoc BDNF; ↓ cortical and Hippoc BDNF levels in Nrf2^(−/−)^ mice; these were ↑ by chronic fluoxetine	Chronic fluoxetine reverts CORT-induced Nrf2 pathway changes in cortex and Hippoc in a mouse model of depression; Nrf2 enhances BDNF, but fluoxetine enhances cortical and Hippoc BDNF through both Nrf2-dependent and independent pathways
Cunha et al., 2016 [45]	Adult ♂ Swiss mice (30–40 g)	Stress-induced DLB	Florianópolis, SC, Brazil	1. I group: creatine or plac + LY294002 icv (PI3K inhibitor), wortmannin icv (PI3K inhibitor), or plac→TST or OFT 2. II group: lithium chloride (nonselective GSK3 inhibitor) or ARA01441 (selective GSK3 inhibitor)→TST or OFT 3. III group: subeffective doses of creatine + subeffective doses of GSK3 inhibitors (ARA01441 or lithium chloride)→TST or OFT 4.1 IV group: CoPP icv (HO-1 activator)→ TST or OFT; creatine or plac + HO-1 inhibitor ZnPP icv or plac→TST and OFT; creatine or plac + CoPP icv or plac→TST or OFT 5. V group: creatine or plac + rapamycin (mTOR inhibitor) or plac→TST and OFT	Treatment with creatine↑ Akt and P70S6K phosphorylation, HO-1/Nrf2, GPx and PSD95 immunocontents. The pretreatment with LY294002 wortmannin, ZnPP (HO-1 inhibitor), or rapamycin (mTOR inhibitor) prevented the AD-like effect of creatine in the TST. Subbeffective dose of either the ARA014418, lithium chloride, or the HO-1 inductor CoPP + subeffective dose of creatine ↓ the immobility time in the TST	The AD-like effect of creatine in the TST depends on the activation of Akt, Nrf2/HO-1, GPx, mTOR, and GSK3 inhibition
Freitas et al., 2016 [46]	♁, Swiss mice, WT C57BL/6 mice (Nrf2^+/+^) and Nrf2 KO (Nrf2^−/−^) (3–months-old, BW 40–45 g)	Mouse CORT model of DLB	Madrid, Spain	1. Mice assigned to 6 groups (8 mice each): (a) plac, (b) IMI/plac, (c) agmatine/plac as the Ctrl groups, (d) plac/CORST, (e) IMI/CORST, and (f) agmatine/CORST. 2. Mice were assigned to 3 groups (6 mice each): (a) Nrf2^+/+^/plac, (b) Nrf2^+/+^/agmatine, as the Ctrl groups, (c) Nrf2^−/−^/plac, and (d) Nrf2^−/−^/agmatine	Agmatine ↓ CORST-induced DLB, ↑BDNF, synaptotagmin I, Ser and Glut levels; ↓the CORST-induced changes in the morphology of astrocytes and microglia in CA1 subregion of Hippoc; ↑ Nora, Ser, and Dopa levels, CREB phosphorylation, mature BDNF and synaptotagmin I immunocontents, in the Hippoc of Ctrl group. Agmatine’s ability to produce an AD-like effect was abolished in Nrf2^(−/−)^ mice	Chronic administration of a low dose of agmatine is able to abolish the behavioural responses in the TST and splash test elicited by the CORST-induced model of depression by a mechanism dependent on the activation of Nrf2 and neuroplasticity-related signalling in mice
Martín-Hernández et al., 2016 [47]	♂ outbred Wistar Hannover rats initial BW 200–225 g	CMS-induced DLB	Madrid, Spain	The following groups (n 8 each) were used: (1) Ctrl; (2) Ctrl group +ip injection of sterile plac for 7 days (CT þ Veh), (3) CMS group; (4) a CMS group+ ip plac (CMS þ Veh) group. For experiments requiring the ip injection of AD, 3 other experimental groups were used: (5) CMS group + desipramine; (6) CMS group + escitalopram and (7) CMS group +duloxetine→behav tests + biochemical analysis	In the PFC, CMS ↓Akt and PI3K mRNA expression. Desipramine and duloxetine ↑ CMS-induced Akt levels, but only desipramine restored PI3K levels. CMS ↓Nrf2 mRNA and protein expression levels. Nrf2 inhibitors Keap-1 and p-GSK-3β/GSK-3β ratio ↑ after CMS. Desipramine and duloxetine to CMS rats restored the expression of Nrf2, returned Keap-1 to Ctrl levels and showed a trend towards returning the p-GSK-3b/GSK-3b ratio to its Ctrl levels. CMS ↓ NQO-1, GPx1.AD treatment restored GPx1 levels. Desipramine ↑HO-1. PAR g is modulated by the AD treatments in the PFC	Nrf2 pathway is differentially regulated by AD in the PFC and Hippoc. The Nrf2 pathway is involved in the oxidative/nitrosative damage detected in the PFC, and AD has a therapeutic action through this pathway. It seems that Nrf2 is not involved in the effects caused by CMS in the Hippoc
Martín-Hernández et al., 2016 [48]	♂ outbred Wistar Hannover rats; initial BW 200–225 g	CMS-induced DLB	Madrid, Spain	Mice divided into 3 groups (n = 8 each): (1) a Ctrl group; (2) CMS group; (3) CMS group treated with antibiotics (CMS þ ATB)→behav test+ biochemical analysis	CMS protocol ↑intestinal permeability and bacterial translocation. CMS also ↑the expression of the activated form of the MAPK p38 while ↓the expression of Nrf2. The actions of antibiotic administration to prevent bacterial translocation↓MAPK and ↑ Nrf2 pathways	Translocated bacteria could play a role in the pathophysiology of depression through the p38 MAPK pathway, which could aggravate the neuroinflammation and the oxidative/nitrosative damage present in this pathology. Moreover, Nrf2 and its activators may be involved in the consequences of the CMS on the brain
Wojnicz et al., 2016 [49]	Adult ♂ Sprague Dawley rats (2.5–3 months old and BW 250–300 g); Nrf2 KO mice	Nrf2 KO mouse model of depression	Madrid, Spain	Seven adult WT rat brain samples→ LC–MS/MS to detect concentrations of neurotransmitters (Adre, Nora, Glu, GABA, DA, 5-HT) and their metabolites (MHPG and 5-HIAA)c. Same procedure in the Hippoc samples of Nrf2 KO mice	LC–MS/MS in adult WT and Nrf2 KO rats showed no significant differences in neurotransmitter values except for GABA, which was strongly ↓ in KO rats	LC–MS/MS method enables rapid quantification of neurotransmitters and their metabolites. It was precise, accurate, sensitive and reproducible. Its application to the mouse model of depression (Nrf2 KO) recorded a↓of Hippoc GABA
Yao et al., 2016 [50]	Adult ♂ C57BL/6 mice, aged 8 wk (BW 20–25 g) and 5 wk; CD-1 mice, aged 14 wk (BW 40–45 g) and ♂adult Nrf2 KO (Nrf2^−/−^) mice	SDS model of depression	Chiba, Japan	WT and KO mice subjected to SDS to induced DLB (exposed to a different CD1 aggressor mouse each day for 10 min for 10 days)→I group: DLB mice + SFN (10 mg/kg), II group: DLB mice + 7,8-DHF (10 mg/kg); III group: DLB mice + ANA-12 (0.5 mg/kg); IV group: Ctrl→behav tests+ biochemical analysis	↓ Keap1 and Nrf2 in the PFC, CA3 and DG of Hippoc in mice with DLB compared to Ctrl; ↑ serum levels of pro-inflammatory cytokines in Nrf2 KO mice compared to WT mice; ↓ BDNF and TrkB in PFC, CA3 and DG play a role in DLB of Nrf2 KO mice. TrkB agonist, 7,8-DHF, but not antagonist ANA-12, produced AD effects in Nrf2 KO mice. Pretreatment with Nrf2 activator sulforaphane (SFN) prevented the DLB induced after repeated SDS	Keap1-Nrf2 system plays a key role in depression and dietary intake of SFN-rich food during juvenile stages and adolescence can confer stress resilience in adulthood (dietary intake of 0.1% glucoraphanin (a precursor of SFN) containing food during juvenile and adolescent stages also prevented the depression-like phenotype evoked in adulthood, after repeated social defeat stress)
Yao et al., 2016 [51]	Adult ♂ C57BL/6N mice BW 20–26 g	LPS-induced DLB	Chiba, Japan	Mice received ip injection of LPS (0.5 mg/Kg)+ Nrf2 activators TBE-31 or MCE-1→behav tests + ELISA	TBE-31 and MCE-1 ↑ nerve growth factor (NGF)-induced neurite outgrowth in PC12 cells in a concentration-dependent manner. TBE-31 or MCE-1↓ an increase in serum levels of TNF-α after LPS administration. In the TST and FST, TBE-31 or MCE-1 ↑ the mobility time after LPS administration	The Nrf2 activators have AD effects in animal models of depression. The novel Nrf2 activators such as TBE-31 and MCE-1 might be potential therapeutic drugs for inflammation-related depression
Bouvier et al., 2017 [52]	♂ Sprague Dawley rats BW 290–310 g (intruder rats); ♂ WT Groningen rats (WTG, resident rats); C57BL/6J background WT (Nrf2^+/+^) and Nrf2-KO (Nrf2^−/−^) 6-wk-old mice	SDS and CMS-induced DLB	Paris, France	Rats received intense stress first hit produced (SD) + second stressful hit (CMS)→ behav test and biochemical analysis; antioxidants were Tempol, 4-hydroxy-2,2,6,6-tetramethylpiperidine 1-oxyl; 288 μmol/kg^−1^·day^−1^, 7,8-DHF on days 5, 7 and 9 after the end of the social defeat protocol, and *t*-BHQ (continuous infusion during 6–7 days)	Only vulnerable animals developed a DLB after CMS derived from a persistent state of OS and reversed by treatment with antioxidants. This persistent state of OS was due to ↓ BDNF levels. ↓BDNF→↓ nuclear translocation of Nrf2. In Nrf2^+/+^ mice, the activation of Nrf2 translocation restored redox homoeostasis and reversed vulnerability to depression. This mechanism was absent in Nrf2^−/−^mice	Low BDNF levels in vulnerable animals prevented Nrf2 translocation and consequently prevented the activation of detoxifying/antioxidant enzymes, resulting in the generation of sustained OS
Zhang et al., 2017 [53]	♂ adult C57BL/6 mice, aged 8 wk (BW 20–25 g)	LPS-induced DLB	Chiba, Japan	Mice received an injection of LPS and SFN→behav test and biochemical analysis. One subgroup of mice received a dietary amount of 0.1% glucoraphanin (a glucosinolate precursor of SFN) at 5 wk→behav test in adulthood (9 wk)	Pretreatment with SFN blocked an ↑ in the serum TNF-α level and an ↑ in microglial activation after LPS administration (0.5 mg/kg); SFN ↑serum IL-10 after LPS administration. In the TST and FST, SFN ↓immobility time after LPS administration, SFN significantly recovered to Ctrl levels for LPS-induced alterations in the proteins such as BDNF, postsynaptic density protein 95 and AMPA receptor 1 (GluA1) and dendritic spine density. Dietary intake of 0.1% glucoraphanin (SFN precursor) food during the juvenile stage and adolescence could prevent the onset of LPS-induced DBS	Dietary intake of SFN-rich broccoli sprouts has prophylactic effects on inflammation-related depressive symptoms→supplementation of SFN-rich broccoli sprouts could be a prophylactic vegetable to prevent or ↓ the relapse by inflammation in the remission state of depressed patients
Zhao et al., 2017 [54]	C57BL/6J ♂ mice (adult, 8-wk-old) BW 20–25 g	CRS and ARS-induced depression	Nanjing, Jiangsu, China	Mice subjected to 2 stress paradigms: 8 wk of CRS and 2 h ARS; mice divided into 2 groups: prolonged (4 wk) and short-term (a single inj) Ipt treatment (i.p. 10 mg/kg)→behav tests+ biochemical analysis (ELISA, RT-PCR, WB, IF)	HPA axis was altered after stress, with different responses to CRS (↓r ACTH and CORT, ↑ AVP, but normal CRH) and ARS (↑ CRH, ACTH and CORT, but normal AVP). Prolonged and short-term Ipt treatment normalised stress-induced HPA axis disorders and abnormal behav in mice. CRS and ARS ↑mRNA levels of TNFα, IL-1_, IL-6 and TLR4 and OS molecules (gp91phox, iNOS and Nrf2) in the hypothalamus. IF showed CRS and ARS ↑ microglia activation (CD11b and TNF_) and OS in neurons (NeuN and gp91phox), which were ↓ by Ipt	Activation of ATP-sensitive potassium channel by ipt normalises stress-induced HPA axis disorder and depressive behav by alleviating inflammation and OS in mouse hypothalamus
López-Granero et al., 2017 [55]	BXD RI strains and C57BL/6 WT mice (5–6 wk), ♂, ♁	BXD recombinant inbred mice depression and anxiety model	New York, NY, USA	Two BXD RI mouse strains, BXD21/TyJ RI, BXD84/RwwJ RI and C57BL/6 WT mice were used with 12 animals per strain and 6 animals per sex→behav tests + biochemical analysis	BXD84/RwwJ RI exhibits social avoidance behav and ↓time in elevated open spaces during the EMT. BXD21/TyJ RI ↓immobility time in the and ♂-specific sensitivity is noted; they also ↑Nrf2mRNA levels (no changes in Keap-1). Same cerebral cortex Gpx1 mRNA in BXD21/TyJ RI, BXD84/RwwJ RI and C57BL/6 WT mice. ↑pro-inflammatory response in ♂ BXD21/TyJ RI compared to BXD84/RwwJ RI and C57BL/6 WT (↑ IL-6 and TNF mRNA)	BXD84/RwwJ RI strain exhibits anxiety disorders, emotional disorders, anxiety-like behav, and social avoidance-like behavior (2) BXD21/TyJ RI strain shows resistance to depression illness
Abuelezz and Hendawy, 2018 [56]	Adult ♂ Wistar rats BW 150–200 g	CRS-induced DLB	Cairo, Egypt	Animals were allocated randomly to one of the following 5 groups (n 12 each): non-restrained Ctrl group, CRS group, and 3 other CRS-groups treated with cilostazol, a phosphodiesterase-3 and ROS inhibitor (7.5, 15, 30 mg/kg/day for 4 wk)→SPT, OFT, FST+ Biochemical, RT-PCR, WB analysis	Hippoc cytoplasmic and nuclear Nrf2 expressions were ↓ in CRS-rats, as well as HO-1 and NQO-1mRNA, compared with the Ctrl group. Cilostazol (15 mg/kg/day) prevented ↓nuclear Nrf2, whereas cilostazol (30 mg/kg/day) prevented ↓ in cytoplasmic and nuclear Nrf2 expression. Cilostazol (15 mg/kg/day) prevented ↓ in HO-1, whereas cilostazol (30 mg/kg/day) prevented the decrease in both HO-1 and NQO-1 mRNA	Cilostazol prevented CRS-induced DBL, improving behav tests and hypothalamus–pituitary–adrenal axis hyperactivity. Cilostazol prevented CRS-induced ↑ in Hippoc lipid peroxidation and 8-hydroxy-2′-deoxyguanosine, and a ↓ in antioxidant activities
Omar and Tash, 2017 [57]	50 Adult ♂ Sprague Dawley rats BW 180–220 g	Chronic mild stress model of depression	Cairo, Egypt	Rats divided into the Ctrl (n = 10) and stress (n = 40) groups. Ctrl rats received distilled water. The stress group, subjected to the CMS procedure, was further subdivided into 4 subgroups (n 10 each): I group = distilled water; II group = fluoxetine (10 mg/kg/day); III group = zinc (15 mg/kg/day); IV group = fluoxetine + zinc (treatment for 28 days)→behav tests + biochemical investigations (ELISA, WB, RT-PCR)	Hippoc mRNA and protein levels of Nrf2, HO-1, MTs, GPR39 and BDNF ↑ in response to a combined therapy of fluoxetine and zinc than to either monotherapy. HO-1 and MTs gene expression was correlated with that of Nrf2 in the fluoxetine-only group	Fluoxetine therapy activated the expression of MTs and HO-1 through an Nrf2-dependent pathway. When fluoxetine was escorted by zinc, activated MTs had a positive impact on BDNF through the zinc signalling receptor GPR39, resulting in ↑in neuronal plasticity as well as ↓ of neuronal atrophy and neuronal cell loss
Li et al., 2017 [58]	8- to 10-wk-old ♂ ICR mice	LPS-induced DLB	Ningbo, Zhejiang, China	Mice treated with IL-1β shRNA lentivirus or NS shRNA (Ctrl) lentivirus by DG regions inj + LPS (1 mg/kg, i.p.) or plac→ behav tests (memory deficits with NORT; anxiety-like behaviors with EZM; DLB with SPTand FST). Furthermore, the levels of MDA, SOD, Nrf2, HO-1, TNFα, VGF and BDNF were assayed	IL-1β KO in the Hippoc ↓ the memory deficits, anxiety- and DLB induced by LPS in mice; it also ameliorated the oxidative and neuroinflammatory responses and abolished the ↓ of VGF and BDNF induced by LPS. Finally, the ↑MDA and ↓SOD, Nrf2 and HO1 induced by LPS were completely prevented with IL-1β shRNA	IL-1β is necessary for the oxidative and neuroinflammatory responses produced by LPS and offers a novel drug target in the IL-1β/oxidative/neuroinflammatory/neurotrophic pathway for treating neuropsychiatric disorders that are closely associated with neuroinflammation, OS and ↓ of VGF and BDNF
Yang et al., 2018 [59]	24 ♂ Sprague Dawley rats, 8-wk-old	LPS-induced DLB	Jining, China	Three groups of 8 rats each: Ctrl, LPS, and LPS + NPB. 24 h after last injection, behav tests + brain tissue analysis	Nrf2 ↓ in LPS group, Nrf, HO1 and NQO-1 levels ↓ in NBP group	Prolonged NBP treatment ameliorated LPS-induced DLB, attenuating LPS-induced NI, and OS
Gao et al., 2019 [60]	♂ CD-1 mice BW 23–25 g and 8-wk-old ♂ C57BL/6 J mice	Effects of allicin on DLB	Yichang, China	Five groups 10 mice each: Ctrl, CSDS, CSDS + allicin (2, 10, or 50 mg/kg). SPT, SIT, and FST → Hippoc tissue collected. Inflam mediator levels assayed through ELISA. Iron concentration and iron-related protein expression Measur by WB. OS and apoptosis markers detected by WB	Allicin ↓ production of ROS, MDA NOX4, and ↑ activities of SOD and Nrf2/HO-1 pathway; CSDS mice performed worse than Ctrl on SPT, SIT, and FST; allicin reversed these impairments, with the highest dose being more effective	Microglia activation and ↑ cytokine in Hippoc of CSDS were ↓ by allicin. Content of iron and protein expression of iron metabolism were aberrant in CSDS mouse Hippoc; allicin improved this phenomenon. It also attenuated enhanced neuronal apoptosis and promoted NLRP3 inflammasome suppression (↓ Hippoc ASC, caspase-1, and IL-1β)
Fan et al., 2018 [61]	72 ♂ 220–240 g Wistar rats	CUMS-induced DLB	Jinan, China	Four groups with N = 18/group: (a) Ctrl (non-CUMS), (b) CUMS, (c) ginsenoside-Rg1 pretreatment (40 mg/kg), (d) ginsenoside-Rg1 pretreatment (40 mg/kg) followed by CUMS. Behav tests + brain removed for immunofluorescence assay, immunohistochemistry and TUNEL staining	Ginsenoside-Rg1 ↑ Nrf2 expression and inhibits p-p38 MAPK and p65 NFκB subunit activation within the vmPFC	Ginsenoside-Rg1 prevented depression-like effects in a rat CUMS model. Chronic ginsenoside-Rg1 pretreatment prior to stress exposure suppressed inflam pathway activity via ↓ proinflam cytokine overexpression and microglial/astrocytic activation; ↓ dendritic spine and synaptic deficits parallel to ↑ synaptic-related proteins in vmPFC. ↓ apoptosis induced by CUMS exposure, ↑ Bcl-2 expression and ↓ cleaved caspase-3 and caspase-9 expression within the vmPFC region
Chu et al., 2019 [62]	24 ♂ pathogen-free Sprague Dawley, 6-wk-old rats + 30 WT and 30 Nrf2^−/−^ KO ♂, 6-wk-old mice	Pollution-induced DLB; tested the Nrf2/NLRP3 pathway in DLB	Shijiazhuang, Hebei Province, China	Twenty-four rats randomised into 3 groups: exposed to FiA, UnA, and CA × 12 wk. 30 WT and 30 Nrf2^−/−^ KO mice randomised into clean air exposure and to UnA × 9 wk. Mice and rats had behav testing. Toxic elements in PFC of rats after PM2.5 exposure were Measur by ICP-MS; neurotransmitter and their metabolites’ determination (NA, 5-HIAA, 5-HT, DA, L-Dopa, DOPAC), GSH and GSSG levels in PFC were Measur by HPLC; histopathological changes, neurotrophic factor levels, cytokines, and NLRP3 inflammasome-related protein expression in PFC of rats detected with IHC and WB	CA rats and KO-UA mice displayed depressive-like responses. The NLRP3 signalling pathway was more activated in Nrf2^−/−^ KO than WT mice after PM2.5 exposure × 9 wk	Li, Be, Al, Cr, Co, Ni, Se, Cd, Ba, Ti and Pb were deposited in rat PFC after PM2.5 exposure. Neurotransmitters were significantly altered in PFC of CA rats. The NLRP3 signalling pathway was more activated in Nrf2^−/−^ than WT mice after PM2.5 exposure × 9 wk. The Nrf2/NLRP3 signalling pathway, by modulating inflammation, might play an important role in ambient PM2.5-induced depression
Dang et al., 2019 [63]	Adult, 8-wk-old C57BL/6 ♂ mice	PCMS in LPS-induced DLB	Xi’an, 710032, Shaanxi, China	Mice with PCMS (5 min with no mobility × 4 wk) and stress-naïve mice. LPS or plac administered via ip injection → Behav tests (FST, OFT, E + M) → brain removal, analysis through IF to detect IBA-1, IL-1β, Nrf2; WB for NLRP3, ASC, caspase-1, Nrf2, HO-1, NQO-1,TXNIP, Trx and β-actin; Real-time PCR to assess the amount and integrity of total Hippoc RNA	mRNA and protein levels of Nrf2 in stress naïve mice ↓ 26 h post-LPS administration compared with plac-treated mice. Though a significant difference in Nrf2 protein levels was not observed, PCMS mice showed increased gene expression of Nrf2 compared with stress-naïve mice; stress-naïve mice performed worse than PCMS mice on FST, OFT, and E + M	PCMS promotes recovery from LPS-induced behav deficits. Stress naïve mice showed nuclear condensation and acidophilic degeneration after LPS treatment; these neuronal injuries were alleviated in PCMS mice. IF for IBA-1 was used to analyse microglial activation, which was attenuated in PCMS mice. PCMS ameliorated LPS-induced OS, with decreased MDA level, enhanced SOD activities and reduced 8-OHdG. Gene expression of pro-apoptotic Bax was largely ↑ in the Hippoc of stress-naïve mice 26 h post-LPS and ↓ in PCMS mice. PCMS mice showed partially inhibited NLRP3 inflammasome activation (↓ in NLRP3 inflammasome component levels and attenuated IL-1β and TNF-α expression)
Gao et al., 2019 [64]	50 5-wk-old ♂ C57 mice	HFD-induced DLB	Yichang, China	To study OS, MCh function, autophagy, insulin resistance, and NOX/Nrf2 imbalance, mice were randomised into 5 groups of 10 each: Ctrl, HFD, HFD + allicin (50, 100, or 200 mg/kg). After HFD and allicin × 15 wk → behav testing. Blood samples were collected after 12 h fasting periods. All hippocampi were removed for subsequent detection	↑mRNA and protein expressions of NOX2 and NOX4, ↓Nrf2/HO-1 signalling in Hippoc of obese mice. Allicin ↓ NOX2/NOX4 expression and ↑ Nrf2/HO-1 levels	Allicin ↓ weight of obese mice, metabolic indicators, CORST, IR, and corrected HFD-triggered aberrant insulin signalling. HFD induced DLB, which was ameliorated by allicin.↑ ROS, MDA, protein carbonylation triggered by HFD were inhibited by allicin. HFD caused ↑ protein expression of autophagy in the Hippoc, which was reverted by allicin. Allicin ameliorated OS-induced damage through ↑ antioxidant SOD, CAT, GSH, and GPx activity
Arioz et al., 2019 [65]	♁ Balb/c, 12–14-wk-old mice	OS, LPS-DLB	Izmir, Turkey	Effect of MT on NLRP3 inflammasome activation and SIRT1/Nrf2 pathway. Mice randomised into 3 groups: Ctrl, LPS, MT (30 mg/kg × 4) + LPS (5 mg/kg ip). 24 h later, animals performed behav experiments TST, FST → sacrificed. Hippocampi were isolated and used for further analyses; glial cell culture	MT ↑ Nrf2 translocation to nucleus (WB) and Nrf2 target genes HO-1, NQO1, GSTP1, GCLM (qPCR). Cross-talk between Nrf2 and SIRT1 protective pathways: siRNA-mediated Nrf2 knockdown inhibited basal SIRT1 expression; siRNA-mediated SIRT1 knockdown ↓Nrf2 translocation. The beneficial effects of MT on NLRP inflammasome activation were associated with Nrf2 and SIRT1	MT ameliorated LPS-induced behav abnormalities in a mouse model of acute systemic inflammation and depression and decreased NLRP3 inflammasome activation in mice hippocampi (qPCR, WB and IF staining). Beneficial actions of MT are partly and significantly dependent on Nrf2 and SIRT1 activation in LPS and ATP-challenged murine microglia
Cigliano et al., 2019 [66]	24 ♂ MRL/lpr mice brain samples (8-, 22- or 17-wk-old)	MRL/MpJ-Faslpr lupus-prone depression murine model	Napoli, Italy	To test CLA and FO modulation of the Nrf2 pathway in a mouse depression model, brain samples from 2 groups (n = 8 each), composed of 8- (Young) or 22-wk old (Old) mice were examined to evaluate the age-dependent occurrence of depressive disorder markers (BDNF, TrkB, Synaptophysin, Synapsin I; Synaptotagmin I, PPAR-α, PPAR-γ and the modification of DHA, C18:1, C16:0, and C18:0 content) with rtPCR and WB. 2 additional groups composed of 17-wk-old mice (n = 8 each) were supplemented with FO or CLA × 5 wk, when they reached old age, and were compared with untreated Old mice	FO or CLA ability in modulating Nrf2 pathway was investigated in brain cortex of all experimental groups. Old animals exhibited higher G6PD and GSR activities. Compensatory hyperactivation of GSR and G6PD, as well as ↑GCL and GSRmRNA levels exhibited by Old mice (*p* < 0.05), were ↓by FO and CLA. ↑Nrf2 involvement in the antioxidant activity elicited by FO or CLA (↓ Nrf2 content in nuclear extracts of FO + Old and CLA + Old animals)	Old mice exhibit disrupted Redox homoeostasis, compensatory Nrf2 hyperactivation, ↓ DHA, ↓ BDNF and ↓ of synaptic function proteins (Synaptophysin, Synaptotagmin I, Synapsin I) compared to Young mice. FO and CLA relieve almost all depression markers at a level comparable to Young mice, improving Nrf2-mediated antioxidant defences, ↓ auto-antibody titre and TNF-α concentration, ↑ BDNF and synaptic function proteins (FO > CLA)
Liu et al., 2019 [67]	♂ WT C57BL/6 mice (adult, PRMT1^+/+^) BW 22–25 g; PRMT1 KO (PRMT1^−/−^) mice with C57BL/6 background	LPS-induced DLB	Liaocheng, China	PRMT1^+/+^ and PRMT1^−/−^mice received plac (10 mL/kg) or LPS (0.5 mg/kg) (ip) → behav testing → sacrifice; Hippoc analysed for total RNA with rt-qPCR. pNF-κB p65, NF-κB, Nrf-2, GFAP, PRMT1 and IBA-1, GAPDH were detected with WB. ROS levels in AST were determined using a specific probe	LPS ↓Nrf-2 expression; PRMT1 deficiency countered this effect. Nrf-2 expression in AST ↓ by ML385, an Nrf-2 inhibitor. PRMT1 KO ↓ expression of IL-1 β and TNF-α in LPS-exposed AST; this was prevented by ML385 pretreatment. PRMT1^−/−^ ↓ ROS generation in LPS-exposed cells; levels were restored by ML385 pretreatment	PRMT1^−/−^ mice ameliorated LPS-induced DLB and ↑BDNF and PSD-95 expression. PRMT1 deletion alleviates LPS-induced brain injury; down-regulating LPS-promoted expression levels of GFAP and IBA-1 compared with PRMT1^+/+^ mice. PRMT1 deficiency ↓IL-1β and TNF-α in Hippoc and PFC of LPS-challenged mice, ↓ pNF-κB, ↑ SOD and GSH-pX activities in Hippoc and ↑ Nrf-2
Rosa et al., 2019 [68]	Adult ♁ Swiss mice (3 months, 30–40 g)	Guanosine AD-like effect via GSK-3β inhibition and MAPK/ERK and Nrf2/HO-1 activation	Florianópolis, Santa Catarina, Brazil	-Effective dose of guanosine (0.05 mg/kg, p.o.)/plac was administered to mice → TST -Sub-effective dose of guanosine (0.01 mg/kg, p.o.)/plac + sub-effective dose of lithium chloride (a non-selective GSK-3β inhibitor, 10 mg/kg, p.o.)/plac → TST, OFT -Sub-effective dose of guanosine/distilled H_2_O + sub-effective dose of the selective GSK-3β inhibitor, ARA014418 (0.01 μg/site, icv)/plac → TST and OFT -Effective dose of guanosine→ Hippoc and PFC WB for β-catenin and Nrf2 immunocontent -Effective dose of guanosine/plac + MEK1/2 inhibitor (5 μg/site, icv)/plac → TST -Effective dose of guanosine/+ ZnPP (HO-1 inhibitor, 10 μg/site, icv)/plac→ TST, OFT and WB Hippoc and PFC analysis for HO-1 detection	Guanosine ↓ immobility time on the TST but did not alter OFT parameters. Guanosine ↑ Nrf2 cytosolic fraction immunocontent in Hippoc and PFC, compared to Ctrl. Nrf2 Hippoc nuclear fraction was not altered with guanosine	The combined treatment with sub-effective doses of guanosine (0.01 mg/kg, p.o.) and selective/non-selective GSK-3β inhibitors produced a synergistic AD-like effect in the TST. The AD-like effect of guanosine (0.05 mg/kg, p.o.) was completely prevented by the treatment with MEK1/2 inhibitors, or ZnPP. Guanosine administration (0.05 mg/kg, p.o.) ↑ the immunocontent of β-catenin in the nuclear fraction and Nrf2 in the cytosolic fraction in the Hippoc and PFC. HO-1 immunocontent was also ↑ in the Hippoc and PFC treated with guanosine. Guanosine ↓ depression by ↓ GSK-3β and ↑ MAPK/ERK and Nrf2/HO-1 pathways
Huang et al., 2020 [69]	18 ♂ C57BL/6 mice (7–8-wk-old)	CMS-induced DBL	Shanghai, China	ADSCs were isolated from mouse fat pads and intravenously administered to CMS-exposed C57BL/6 mice at the dose of 1 × 106/wk × 3 wk. Behav test (SPT, FST, TST) + rt-qPCR analysis of brain RNA, ELISA microglia analysis to detect MCP-1, IL-6, TNF-α, and IL-1β; WB with anti-Nrf2, anti-HO-1, anti-NF-κB1, anti-CD29, anti-CD90, anti-CD44, anti-CD105, anti-CD34, anti-vWF, anti-BDNF, anti-TrkB, and anti-GAPDH Abs	CMS promoted DLB, TLR4/NFκB but suppressed Nrf2/HO-1; ADSC treatment had the opposite effect, ↑SPT and ↓ immobility on TST and FST. ADSC and BV2 microglia cell cocultures showed that the ↑ of TLR4 and NFκB induced by LPS was ↓ by treatment with Nrf2-overexpressing vector ADSCs, while the ↓ of Nrf2 decreased the inhibitory effect of ADSCs on LPS-induced TLR4 and NFκB expression. The ↑ of Nrf2 in ADSCs ↓ BV2 LPS-induced inflammatory factor secretion (MCP-1, TNF-α, IL-1β, and IL-6)	ADSC treatment reversed CMS-induced DLB. The BW of the mice in the CMS group slowly ↓ compared to Ctrl, and ADSC treatment restored the CMS-induced BW reduction. ADSC reversed CMS-induced DBS, CMS-induced inflammatory factor expression, and Hippoc microglial polarisation. CMS ↑ MCP-1, TNF-α, IL-1β, and IL-6 expression in serum, but ADSC reversed CMS-induced inflammatory factor production. Immunohisto-chemical detection also showed that the number of apoptotic neuronal cells ↓ with ADSC treatment. BDNF and TrkB ↓ with but ↑with ADSC. CMS induction promoted TLR4/NFκB signalling but suppressed Nrf2/HO-1 signalling, while ADSC treatment had the opposite effect
Zborowski et al., 2020 [70]	24 ♂ adult Swiss mice, 60 days old, 25–35 g	DLB in STZ-induced DM mice	Santa Maria, Rio Grande do Sul, Brazil	Animals separated into 4 groups (n = 6 each): Ctrl; STZ-induced DM; (p-lPhSe); DM + (p-ClPhSe)2. Groups II and IV received STZ at a single dose of 200 mg/kg. After 14 days, DM+ mice (blood glucose ≥ 200 mg/dL) were enrolled. At day 21, mice performed behav tests (LP, TST, FST). For ex vivo assays, brains were removed, and the samples of the whole cerebral cortex were subjected to WB and OS assays	↓ in Keap1, Nrf2 and HO-1 levels in the cerebral cortex of DM mice compared to Ctrl. (p-ClPhSe)2 ↑ Keap1, Nrf2, and HO-1 levels. A negative correlation was found between glycaemia on the one hand and Keap1, Nrf2 and HO-1 levels on the other	(p-ClPhSe)2 reversed DM+ mice DLB but did not alter mouse spontaneous behaviour; hyperglycaemia; counteracted DM-induced cortical oxidative damage. It did not reverse the ↑ in adrenal gland weight and the DM-induced decrease in GR content. It modulated the Keap1/Nrf2/HO-1 signalling pathway in DM mice and ↓ FJC+ cells (a measure of neurodegeneration) in the cerebral cortex of diabetic mice
Casaril et al., 2020 [71]	♁ BALB/c 5–6-wk-old mice	Tumour-induced DLB	Pelotas, Rio Grande do Sul, Brazil	Mice were injected with 50 μL of tumour cell suspension sc; Ctrl mice were injected with PBS. Once tumours became palpable (day 7), tumour size was monitored wkly, and BW and body temperature were recorded. Treatment with CMI (10 mg/kg, i.g.) or canola oil was initiated at day 14 and continued until day 20. 24 h later, mice were submitted to behav tests followed by killing. PFC and Hippoc samples were analysed	4T1 tumour-bearing mice had ↑ of NFκB, IL-1β, TNF-α, IDO, COX-2, and iNOS and ↓ of IL-10, Nrf2, and BDNF. CMI treatment ↓ the expression of inflammatory markers and ↑ the expression of IL-10, Nrf2, and BDNF	CMI abolished tumour-induced DLB and cognitive impairment; ↓ tumour-induced NI (↓NFκB, IL-1β, TNF-α, IL-10, IDO, and COX-2) and OS (altered expression of iNOS and Nrf2, ROS, NO, lipid peroxidation, and SOD activity) in mouse PFC and Hippoc
Tian et al., 2020 [72]	Adult ♂ Sprague Dawley rats (8–12-wk-old), BW 180–220 g	CUMS-induced DLB	Xi’an, Shaanxi, China	CUMS was used to establish depression and anxiety-like behaviour in rats. The rTMS was performed with a commercially available stimulator for 7 days, and then depression and anxiety-like behav were Measur. Nrf2 expression was Measur by WB and TNF-α, iNOS, IL-1b, IL-6 Measur with ELISA. A small interfering RNA was employed to knockdown Nrf2, after which the neurobehav assessment, Nrf2 nuclear expression, and the amount of inflammation factors were evaluated	CUMS-exposed rats had ↓ Nrf2 expression compared to Ctrl (F_1,8_ = 2.97, *p* < 0.05). One-wk rTMS treatment ↑ nuclear Nrf2 protein expression compared to CUMS (F_1,18_ = 3.48, *p* < 0.05)	Application of rTMS exhibited significant AD and anxiolytic-like effects associated with ↑ Nrf2 nuclear translocation and ↓ level of TNF-α, iNOS, IL-1β, and IL-6 in the Hippoc. Following Nrf2 silencing, AD and anxiolytic-like effects produced by rTMS were abolished. Moreover, the ↑ of Nrf2 nuclear translocation, and the ↓of TNF-α, iNOS, IL-1β, and IL-6 in Hippoc mediated by rTMS, were reversed by Nrf2 knockdown
Li et al., 2020 [73]	6-wk-old ♂ ICR mice, 20−22 g	LPS-induced DLB	Liaocheng, ShanDong, China	For the acute inflammation experiment, mice received ip plac or ip Fen (10, 20 and 40 mg/kg) × 7 days prior to LPS injection. After behav tests, all mice were sacrificed. Blood was collected. Brain tissues were isolated for further analysis (siRNA, WB)	Fen dose-dependently ↑ Nrf2 expression from mRNA and Nrf2 protein levels and ↓ Nrf2 ubiquitination. Fen treatment ↑ Nrf2 expression and nuclear translocation in mouse bEnd.3 cells, promoting Nrf2-ARE transcription activity. Nrf2, HO-1, NQO1, and GCLM mRNA; Fen-induced protein expression levels were abolished by Nrf2 knockdown	Fen ↑ antioxidant capacity in bEnd.3 cells after LPS exposure: ↑ SOD, ↑ GPx, ↑ CAT, ↓ ROS, ↓ MDA; ↓ apoptotic rate promoted by LPS; ↓ IL-1β, IL-18, IL-6, TNF-α, and NO; ↓ TNF-κB nuclear expression; ↓ phosphorylation of IKKβ, IκBα and NF-κB. Fen had minimal impact on mouse histological changes and could alleviate symptoms of LPS-induced DLB
Nakayama et al., 2020 [74]	Adult ♂ and ♁ WT Japanese rice fish (medaka, *Oryzias latipes*)	Seasonal changes-induced DLB	Nagoya, Japan	Medaka fish under winter conditions (SC) were divided into 2 groups: one remained in SC with the other transferred to summer-like conditions (LW). 2 wk later, behav tests. Metabolomic and transcriptomic whole brain analyses (microarray analyses, qPCR). Drug screening was conducted to treat winter-induced depression (celastrol)	Inactivation of Nrf2-mediated antioxidant response under winter-like conditions. Celastrol induced Nrf2 expression and NRF2 target genes (GSTω1, GSH, GPx, PG reductase 1, proteasome subunit α type-6,and β type-7 and c-x-c chemokine receptor type 2)	SC in medaka fish: ↓ sociability; ↑ anxiety-like behav; ↓ in circadian clock genes (*PER2*, *PER3*, *BMAL1*, *CLOCK*, *NPAS2*, *CRY2*); ↓ GSH, tryptophan, and tyrosine;↑ inflammation markers (IL6, IL10, and BAFF, IL1R2); ↑serotonin levels but ↓ serotonin turnover, ↑glutamate and ↓ taurine; inactivation of RAR and glucocorticoid receptor signalling, with HPA dysregulation. Celastrol activated Nrf2 pathway
Ali et al., 2020 [75]	8–10 wk ♂ C57BL/6J mice, divided in 5 groups of 6: normal, plac, LPS (1 mg/kg/day), LPS + MT (10 mg/kg/day), LPS + Fluoxetine (10 mg/kg/day), MT (10 mg/kg/day)	OS; LPS-induced DLB	Shenzhen, Guangdong, China	Open field test, Sucrose preference test, FST, TST, ROS-Measur; ELISA, IF, WB for *ATG* gene products and FOX03a	↑ NF-κB signalling in LPS-treated mice, associated with alterations of redox molecules (Akt, Nrf2, HO-1), which were reversed by MT treatment	MT ↓LPS-induced DLB and autophagy impairment in the brain (via FOX03a signalling), ↓LPS-induced OS and NInfl
Wang et al., 2020 [76]	6 wk ♂ C57BL/6J mice, divided in 5 groups of 8: Ctrl; (10 mg/kg) PB; CUMS; CUMS + (10 mg/kg) PB; CUMS + (10 mg/kg) IMI	CUMS-induced DLB mouse model; OS	Nanchang, Jiangxi, China	SPT, OFT, FST, TST, commercial kit for ROS, WB, TUNEL assay	PB treatment → Nrf2 and HO-1 expression, indicating that PB alleviated DLB in mice via activating Nrf2/HO-1 signal pathway	PB alleviated the ↓of sucrose preference and BW, ↓CUMS-induced DLB, ↓ ROS concentrations and inhibited cell apoptosis in Hippoc of CUMS-induced mice. PB ↓ DLB via inhibiting OS and NI, resulting in ↓cell apoptosis in CUMS-induced mice
Liao et al., 2020 [77]	24 ♂ Sprague Dawley rats, 3 groups of 8: Ctrl; CMS group (×4 wk); CMS + SalB (30 mg/kg/day)	DLB in CMS-treated rats	Changsha, Hunan, China	WB, PCR (biomarkers of NI); SPT, FST, NSFT	SalB reversed CMS-induced up-regulation of the gene expression of IL-6, IL-1β, and TNF-α in the Hippoc; SalB → anti-inflammatory effect by activating the Nrf2 signal	SalB could alleviate CMS-induced damage to Hippoc neurones. SalB normalised behav changes in CMS rats
Severo et al., 2020 [78]	46 ♂ 32-day-old Swiss mice in 4 groups: Ctrl; receiving each 4 cncs, 7 cncs; 10 cncs	Protocol of recurrent cncs (4, 7, or 10)	Santa Maria, Rio Grande do Sul, Brazil	TST, HRR, MCh respiration assays, estimation of ROS production and SOD activity, WB	Recurrent cncs did not alter SOD activity, but ↑ expression of NRF2 and SOD2	Cncs ↓MCh oxygen flux vs. Ctrl; cncs do not induce significant changes in TST
Ali et al., 2020 [79]	8 wk C57BL/6J ♂mice in 7groups of 6): normal plac, LPS (1 mg/kg/day), LPS + MT (10 mg/kg/day), LPS + Fluoxetine (10 mg/kg/day), MT (10 mg/kg/day), LPS + MT + luzindole (5 mg/kg/day), LPS + luzindole	LPS induced-DLB	Shenzhen, Guangdong, China	OFT, SPT, FST, TST, Serum ROS Measur, TBARs assay, ELISA (IL-6, IL-1B, TNFa), Immunofluorescence, WB (Nrf2, p-NFkB, NFkB, p-GSK-3β, GSK-3β, Sirt, Ho-1, GAPDH)	MT treatment significantly ↑ Nrf2 and anti-inflammatory protein HO-1 expression which was down-regulated in the presence of MT receptor (MT_1_/MT_2_) inhibitor, suggesting that MT regulates NF-kB/Nrf2/HO-1 expression in a receptor-dependent manner	MT suppressed LPS-induced DLB, ↓cytokines level, ↓oxidative stress, and normalised LPS-altered Sirt1, Nrf2, and HO-1 expression
Camargo et al., 2020 [80]	♂ Swiss mice (30–40 g, 45–60 days of age) divided into 8 groups: plac + plac; plac + ketamine (0.1 mg/kg); plac + guanosine (0.01 mg/kg); (4) plac + ketamine + guanosine; CORT + plac; CORT + ketamine; ketamine+ guanosine; CORT + ketamine + guanosine	CORT-induced animal model of depression	Florianópolis, Santa Catarina, Brazil	Behav tests (TST, OFT, SPlT); WB (GR, NF-κB, IDO-1, GLT-1, Nrf2, HO-1); biochemical analysis (Glutamine synthetase activity, determination of antioxidant enzyme activities and OS markers)	CORT administration ↓ Nrf2 (cytosolic fraction) and HO-1 immunocontent in the Hippoc; a single coadministration with KT + GN could not restore CORT-induced down-regulation on Hippoc Nrf2 and HO-1	Single administration of ketamine (0.1 mg/kg, i.p.) + guanosine (0.01 mg/kg, p.o.) ↓ DLB and Hippoc slice impairments induced by CORT. The behav response obtained by Ketamine + Guanosine was paralleled by the re-establishment of the CORT-induced molecular alterations on Hippoc GR, NF-κB, IDO-1, and GLT-1 immunocontent
Park et al., 2020 [81]	7-wk-old ♂ C57BL/6 mice	Reserpine-induced depression and in vitro LPS-stimulated BV2 microglia	Daejeon, South Korea	Behav tests (OFT, TST, FST); Electrospray Ionisation Mass Spectrometry; IF (for BDNF, cAMP, CREB); ELISA (IL-6, IL-1b, TNF-a, and IL-10); PCR (Il1b, Il6, TNFα, NOs2, Cox2, Hmox1); WB (iNOS, NF-kB p65, HO-1, Nrf2, p-CREB, CREB, p-p38, p38, p-Erk, Erk, p-JNK, JNK, p-Akt, Akt, and BDNF)	BTS ↑ nuclear translocation of Nrf2 and p-CREB, which act as upstream modulators of HO-1 expression in BV2 microglia	BTS has considerable potential as an anti-NI and AD agent, as it has clear effects on depressive behaviours and associated factors caused by reserpine-induced depression. BDNF and pCREB in the Hippoc ↑ in BTS-treated mice vs. reserpine-treated mice. Il1β, Il6, and TNFα mRNA levels in BTS mice were ↓ vs. reserpine-treated mice.
Zhu et al., 2020 [82]	Adult ♂ Sprague Dawley rats BW 200–220 g, 3 groups: Ctrl, low-dose Hsd (50 mg/kg, Hsd-L), high-dose Hsd (50 mg/kg, Hsd-L)	STZ model of type 1 diabetes	Xuzhou, northwestern Jiangsu province, China	Behav test (OFT, TST); ELISA (CORST); Immunohistochemistry (Nrf2)	Hsd caused significant ↑ in Nrf2 levels and up-regulated g-glutamylcysteine synthetase, target gene of Nrf2/ARE signalling	Hsd ameliorate DLB and anxiety-like behaviours of diabetic rats, which are mediated by the enhancement of Glo-1, possibly due to the activation of the Nrf2/ARE pathway
Liao et al., 2020 [83]	♂ Sprague Dawley rats (BW 180–220 g)	CUMS-induced depression model in rats	Changsha, Hunan, China	Rats randomised into 3 groups of 8: Ctrl, CUMS, CUMS + CUR. After 4 wk: behav tests (SPT, FST, OFT, NSFT); Determination of serum CORST; Hippoc: WB (NOX2, 4-HNE, Nrf2, pCREB, CREB, PSD-95, synaptophysin, PCNA), PCR (Nrf2, NQO-1, HO-1); Immunohistochemical staining	Nrf2 signal pathway was inhibited under CUMS, and chronic administration of CUR enhanced Nrf2 translocation from cytoplasm to nucleus and ↑ expression of antioxidant enzymes through Nrf2 signal pathway, thereby protecting the brain against CUMS-induced depression	CUR relieves depressive-like state through the mitigation of OS and the activation of Nrf2-ARE signalling pathway. DLB in CUMS-treated rats successfully corrected after CUR; CUR could effectively ↓ protein expression of OS markers (NOX2, 4-HNE, and MDA) and ↑ the activity of CAT; CUR also reversed CUMS-induced inhibition of Nrf2-ARE signalling pathway along with ↑ the mRNA expression of NQO-1 and HO-1; CUR also ↑ the ratio of pCREB/CREB and synaptic-related protein (BDNF, PSD-95, and synaptophysin); CUR could effectively reverse CUMS-induced reduction in spine density and total dendritic length.
Qu et al., 2021 [84]	♂ adult C57BL/6 mice and ♂ adult Nrf2 KO mice (Nrf2^−/−^) mice	Nrf2 KO mice depression-like phenotypes	Chiba, Japan	Behav tests (LMT, TST, FST, SPT); brain mPFC homogenates: WB for GluA1 and PSD-95	(R)-ketamine(10 mg/kg) could produce rapid-acting and long-lasting AD-like effects in Nrf2 KO mice via the BDNF-TrkB signalling pathway	(R)-KT can produce rapid and long-lasting AD-like actions in Nrf2 KO mice via TrkB signalling: (R)-KT significantly attenuated TST and FST ↑ immobility in Nrf2 KO mice; on the SPT, (R)-KT significantly ameliorated ↓ SPT preference. ↓ expression of GluA1 and PSD-95 in the mPFC of Nrf2 KO mice was significantly improved after a single (R)-KT injection and pretreatment with the TrkB antagonist ANA-12 (0.5 mg/kg) blocked the rapid and long-lasting AD-like effects of (R)-KT. ANA-12 significantly antagonised the beneficial effects of (R)-KT on ↓ expression of synaptic proteins in the mPFC
Li et al., 2021 [85]	Adult C57BL/6J ♂ mice BW 25–30 g	LPS-induced DLB	Shenzhen, Guangdong, China	Animals were divided into four groups of 10: Ctrl, LPS (2 mg/kg/day), LPS + Ibrutinib (50 mg/kg/day), and Ibrutinib (50 mg/kg/day). Behav tests (OFT, SPT, FST); ROS, NO, H_2_O_2_; TBAR Assay, ELISA, IF, Golgi staining, WB (BDNF, Nrf-2, NF-κB, HO-1)	Ibrutinib alleviated redox signalling changes, including altered LPS-induced Nrf2, HO-1, and SOD2 expression; ibrutinib, in the presence of LPS, ↑ the expression of Nrf2 and its target proteins, including HO-1 and SOD2	Ibrutinib ↓ LPS-induced DLB and NI by inhibiting NF-κB activation, ↓ pro-inflammatory cytokine levels, normalising redox signalling and its downstream components, including Nrf2, HO-1, and SOD2, and glial cell activation markers, such as IBA-1 and GFAP; ibrutinib ↓ LPS-activated inflammasome activation by targeting NLRP3/P38/Caspase-1 signalling. LPS ↓ the number of dendritic spines and expression of BDNF, and synaptic-related markers, including PSD95, SNAP25, and synaptophysin, were ↑ by ibrutinib in mouse Hippoc
Yan et al., 2021 [86]	♂ Kunming mice BW 18–22 g	D-GalN-induced animal model	Shenyang, Lioning, China	Four groups: Ctrl, D-GalN, NKT (5 mg/kg), NKT (10 mg/kg). Behav tests (SPT, FST, TST, NFT), WB (Ho-1, Nrf-2)	NKT can effectively ↓ OS in the model group, which may be caused by activating the Nrf2/HO-1/NQO1 signalling pathway, promoting the nuclear translocation of Nrf2, and ↑ the expression of downstream antioxidant protein HO-1 and NQO1 to weaken OS	NKT (5 mg/kg) co-treatment remarkably ameliorates D-GalN-induced anxiety- and depression-like behaviours. NKT could ↑ serum alanine transaminase and aspartate transaminase levels, alleviate hyperammonaemia-induced OS by activating Keap1/Nrf2/HO-1 antioxidant pathways, ↓ the expression of inducible NOs and NOX2 in Hippoc and prefrontal cortex, ↑ the vitality of SOD, ↑ catalase and GSH levels in serum, liver, and brain, and significantly ↓ the generation of MDA. NKT also ↓ the level of ammonia in serum and brain and ↑ the activity of glutamine synthase in the Hippoc and prefrontal cortex
Herbet et al., 2021 [87]	♂ adult Albino Swiss mice BW 25–35 g	Mouse CORT model of DLB	Lublin, Poland	Five groups of 8: (1) Ctrl; (2) stress Ctrl or positive Ctrl of depression: CORST (20 mg/kg) for 21 days; (3) fluoxetine (10 mg/kg) and CORST for 21 days; (4) Mito-TEMPO (1 mg/kg) and CORST; (5) fluoxetine, Mito-TEMPO and CORST. Behav tests (FST, TST). Evaluation of the level of mRNA expression of *Adora1, Ogg1, Msra, Nrf2* and *Tfam* in mouse Hippoc	↑ of *Ogg1, Adora1* and *Nrf2* in the Hippoc of mice receiving CORST and Mito-TEMPO as compared to the CORST Ctrl group	Behavioural research data showed the AD effect of fluoxetine and Mito-TEMPO administered to mice alone and in combination. Molecular findings indicate a significant impact of chronic stress on the oxidation-reduction balance and an antioxidant effect of Mito-TEMPO. The results obtained in the study suggest that Mito-TEMPO protects DNA against oxidative damage and may be beneficial in the way of cellular function improvement under the conditions of chronic stress. *Adora1, Msra, Nrf2* and *Tfam* genes may be involved in mediating the antioxidant effect of the combined fluoxetine–Mito-TEMPO treatment
Yao et al., 2021 [88]	♂ adult C57BL/6 mice (8-wk-old, 20–25 g BW), CD-1 mice (14-wk-old, 40–45 g BW), and ♂ adult Nrf2 homozygous KO mice (Nrf2^−/−^)	LPS-induced and CSDS models of depression; Nrf2 KO mice	Guangzhou, Guangdong, China	PCR for *Nrf2*, *Bdnf*, *Gapdh* genes, and BDNF; WB; Behav tests (locomotion, TST, FST)	Nrf2 activator SFN showed AD-like effects in the LPS-induced and CSDS models of depression by ↑ the expression of BDNF	Activation of Nrf2 by SFN showed fast-acting AD-like effects in mice by activating BDNF, ↓ expression of its transcriptional co-repressors (*HDAC2*, *mSin3A*, and *MeCP2*), and restoring normal synaptic transmission; in contrast, SFN did not affect the protein expression of BDNF and its transcriptional repressor proteins in mPFC and Hippoc, nor did it ↓ DLB and abnormal synaptic transmission in Nrf2 KO mice. In the CSDS mouse model, Nrf2 and BDNF protein levels in mPFC and Hippoc were ↓ compared to Ctrl and CSDS-resilient mice; in contrast, protein levels of BDNF transcriptional repressors in the CSDS-susceptible mice were ↑ than those of Ctrl and CSDS-resilient mice
Salama et al., 2021 [89]	Adult ♂ Wistar albino rats, BW 150 ± 20 g	Ciprofloxacin-induced depression	Cairo, Egypt	Tested camphor as AD. 5 groups. I (normal Ctrl): normal plac. II: camphor (10 mg/kg; i.p.) × 21 days. Group III (depression Ctrl): ciprofloxacin only. Groups IV and V: ciprofloxacin + camphor (5 and 10 mg/kg; i.p.) × 21 days. Behav tests (FST, activity cage, and Rotarod). Measur of OS and antioxidant biomarkers (MDA, NO, Nrf2), inflammatory biomarkers (TLR4, TNF-α), neurotransmitters; histopathology	Camphor ↑ catalase and Nrf-2 activities, ↓ NO, MDA, TNF-α, TLR4 serum levels, and ↑ brain contents of 5-HT, DA, GABA, and P190-RHO GTP protein, normalising fronto-cortical neuronal cell structure and function	The beneficial effect of camphor as AD could be mainly attributed to its antioxidant and anti-inflammatory abilities that ↑ catalase, Nrf-2 expression, and ↓ NO, MDA, TNF-α, and TLR4 production. In addition, it up-regulated P190-RHO GTP protein, an actin reorganiser, thus improving locomotor activity and restoring neurotransmitter function and structure, countering histopathological changes; hence, it may be beneficial in ↓ ciprofloxacin-induced depression
Naß et al., 2021 [90]	*C. elegans* strains (N2 WT, QV225 s*kn-1* deficient, and VC289 *prdx2* deficient)	*Skn-1* (which corresponds to the human Nrf2) and *prdx2-*deficient mutants of *C. elegans*	Mainz, Germany	Examination of the antioxidant activity of UA compared to fluoxetine in *C. elegans* WT and *skn-1-* and *prdx2-*deficient strains through H_2_DCF-DA and jugl., and osmotic and heat-stress assays. Analysis of the binding of UA to human PRDX2 and Skn-1 proteins by molecular docking and microscale thermophoresis	UA exerted stronger antioxidant activities than fluoxetine. Additionally, induction of stress resistance towards osmotic and heat stress was observed. qRT-PCR showed UA to up-regulate *skn-1* and *prdx2* expression	UA exerted antioxidant effects and induced stress resistance through *Prdx2* and *Skn-1*. Additionally, it ↑ the expression of antioxidant genes and prolonged lifespan. In many of these experiments, UA outperformed fluoxetine
Yang et al., 2021 [91]	Adult ♁ BALB/c (10-wk) mice, BW 20–22 g	Cancer-related fatigue model of depression	Changsha, Hunan, China	Test effect of Chinese herb couple Fuzi and Ganjiang (*Aconitum carmichaelii* Debx and *Zingiber officinale* Rosc) on NI (tested on cultured BV2 microglial cells; tested viability, LPS-induction), which in turn induces cancer-related fatigue (tumour inoculation). NO detected through NO_2_^-^, ROS, ELISA, IF, Nrf2 siRNA transfection of BV2 cells, WB, immunohistochemistry, Hippoc and cortex; 7 days post-inoculation, mice randomised into Ctrl, tumour-model, minocycline, low-, intermediate- and high-dose Fuzi and Ganjiang → FST, OFT, TST, and E + M → sacrifice	Fuzi and Ganjiang did not affect BV2 viability, ↓ TNF-α, ↓ IL-6, and ↓ ROS production, abolished iNOS-mediated NO, ↓ COX2-mediated prostaglandin E_2_ and ↓ NF-κB in LPS-induced BV2 microglia; in the same cells, Fuzi and Ganjiang ↑ Nrf2/HO-1 signalling pathway; low and high doses ↓ immobility in the TST, high dose ↑ open arm time in the E + M and ↓ immobility in the FST in tumour-model mice, ↓ iNOS and COX2 in PFC and Hippoc of tumour model mice with cancer-related fatigue–induced depression	Fuzi and Ganjiang counteracted NI and related depression by activating the Nrf2 pathway
Zhu et al., 2021 [92]	♂ Sprague Dawley rats (BW 200–220 g)	DM-associated DLB	Xuzhou, northwestern Jiangsu province, China	Rats received 60 mg/kg, ip STZ injection and were divided into 3 groups of 10 each: DM model group; low dose hesperetin-treated DM group (50 mg/kg), high-dose hesperetin-treated DM group (150 mg/kg) + normal Ctrl → behav tests, biochemical analysis	Hesperetin ↑ Nrf2 and its related genes and proteins (Glo-1 and γ-GCS); ↓ high glucose-induced neuronal damage through the activation of the Nrf2/ARE pathway in SH-SY5Y cells	Hesperetin ameliorated DM-associated anxiety and DLB in rats (↑OFT, SPT, and FST performance) and ↑ Nrf2/ARE pathway activation
Wang et al., 2021 [93]	♂ Sprague Dawley rats (BW 160–180 g)	CUMS-induced depression	Zhengzhou, Henan, China	Rats divided into 5 groups (10 rats each): Ctrl, catalpol; CUMS model; CUMS + catalpol; fluoxetine +CUMS→ behav tests (OFT, SPT, and FST) before and after stress/drug; hippoc for histological and biochemical analysis	CUMS caused ↓ mRNA and protein expression of Nrf2 and HO-1 in rat hippoc, whereas separate administration of both catalpol and fluoxetine reversed CUMS-induced Nrf2 and HO-1 abnormalities	Catalpol improved OFT, FST CUMS-induced abnormalities; ↑ Hippoc PI3K, Akt, Nrf2, HO-1, TrkB, and BDNF (↓ in CUMS-rats); ↑ the Hippoc SOD, catalase, GPX, GSTs; ↑ glutathione levels, ↓ in thiobarbituric acid reactive substances level in CUMS-induced depression
Wu et al., 2021 [94]	Adult ♂ Kunming mice (BW 18–22 g, 3–4-wk-old)	Hyperglycaemia-induced DLB	Zhengzhou, Henan, China	Ten mice selected as the Ctrl group. 80 mice received STZ (150 mg/kg once, ip) injection; 50 mice selected (blood glucose >200 mg/dL) and divided into 5 groups (10 mice each):the plac group; the catalpol (5–10–20 mg/kg)) group; the fluoxetine (20 mg/kg)+ metformin (100 mg/kg) group→ behav tests+ biochemical analysis on brain tissues	The levels of Nrf2 and HO-1 in hippoc and frontal cortex of STZ-induced hyperglycaemic mice significantly ↓, while 20 mg/kg catalpol reversed the abnormal Nrf2 and HO-1 protein levels	Catalpol reversed TST, FST, and OFT abnormalities and abnormal PI3K and Akt phosphorylation; ↑Nrf2-HO1, SOD, GPX and GSTs; ↓ GSH and MDA in hippoc and frontal cortex of STZ-induced hyperglycaemic mice with DLB
Rahman et al., 2021 [95]	Adult BALB/c ♂ mice BW 25–30 g (7–8-wk-old)	LPS-induced DLB	Zhengzhou, Henan, China	Mice divided into 5 groups of 10: (1) plac Ctrl; (2) Plac-LPS (LPS); (3) Xn-LPS (Xn = 10 mg/kg BW, *i.g.*) (4) Xn-LPS (Xn = 20 mg/kg BW, *i.g.*); (5) Fluoxetine-LPS→ behav tests; blood collected brain tissues collected for biochemical analysis	Xn significantly (*p* < 0.001) ↑ Nrf2 and HO-1 expression in the Hippoc, ↓ OS	Pretreatment with Xn (10 and 20 mg/kg, *i.g.*) reversed the behav impairments (FST and TST) with no effect on Locomotion; 20 mg improved anhedonic behavior (SPT). Xn dose-dependently prevented the LPS-induced NI, OS and nitrosative stress; ↓ activated gliosis via ↓ of Iba-1 and GFAP in hippoc; ↓ the expression of p-NF-κB and cleaved caspase-3
Tao et al., 2021 [96]	60 ♁ C57BL/6J mice BW 18–22 g	CUMS induced DLB	Nanjing 210023, China	Four groups: Ctrl, CUMS, CUMS + Magnolol (50 mg/kg, i.g. × 3 wk), and CUMS + Magnolol (100 mg/kg, MA-H i.g. × 3 wk). → behav tests (SPT,OFT,SFT,TST) → sacrificed → Hippoc tissue collected; ELISA for TNF-α, IL-1β, IL-6 IL-4, IL-10; PCR for *Arg1, Ym1, Fizz1* and *Klf4;* Flow cytometry for ROS. Immunofluorescence for Iba-1 + CD16/32+ and Iba-1 + CD206+; WB for Nrf2, HO-1, NLRP3, caspase-1 p20 and IL-1β	Magnolol ↑ Nrf2, HO-1; ↓ NLRP3, caspase-1 p20, IL-1β both *in vivo* and *in vitro*. Magnolol ↓ ROS concentration, promoted Nrf2 nucleus translocation, and prevented Nrf2 ubiquitination. Nrf2 knockdown abolished the Magnolol-mediated microglial polarisation	Magnolol attenuated CUMS-stimulated depression by inhibiting M1 polarization and inducing M2 polarisation via Nrf2/HO-1/NLRP3 signalling
Huang et al., 2021 [97]	♂ Sprague Dawley rats BW 180–220 g	CUMS induced depression model	Wenzhou, Zhejiang, China	Rats divided into 4 groups of 8: Ctrl, CUMS, CUMS+ NC (Hippoc injection LV-pCDH-Nrf2-NC) CUMS+ Nrf2 (Hippoc injection LV-pCDH-Nrf2) → behave tests (SPT, EMZ, OFT, FST, MWM) → sacrifice → Hippoc tissue collected →ELISA for TNF-α, IL-1β, IL-10; WB for Nrf2/β-actin/Wfs1; PCR for miR-17-5P/Nrf2/Wfs1	Nrf2 weakly expressed in CUMS-treated rats. Nrf2 ↓ cognitive dysfunction and inflammatory brain injury. Nrf2 ↓ in CUMS treated rats. Nrf2 up-regulation reversed the trends in behav tests and the changes inflammation-related cytokine levels in CUMS-treated rats. Nrf2 inhibited miR-17-5p → limit Wfs1 transcription. miR- 17–5p ↑ or Wfs1 ↓ reversed the role of Nrf2 in reliving inflammatory injury of murine Hippoc neurones	CUMS group ↓ performance in behave tests. CUMS treated group ↑ TNF-α, IL-1β and ↓ IL-10
Song et al., 2021 [98]	Adult ♂ Kunming mice BW 16–18 g	CORST-induced depression model	Zhengzhou, Henan, China	Four groups of 11: Ctrl group, CORST group, the CORST + catalpol (20 mg/kg) and CORST + fluoxetine (20 mg/kg i-gastr) 21 days → behav test (FST,OFT,TST) → sacrifice → Hippoc tissue, cortex and serum collected → WB for NF-κB/Nrf2/HO-1; ELISA for IL-1β, TNF-α, iNOS, and NO. Cortical tissue ELISA for IL-1β, TNF-α, iNOS, and NO. Detected in the serum level of CORST, ACH and CRH	Catalpol ↑ Nrf2 and antioxidant defence (GSH/GST) and ↓ oxidative damage	CORST → DLB in mice in behav tests, ↑ serum CORT/CRH/ACTH, ↑ NF-κB in the Hippoc and frontal cortex, and ↓ Nrf2. CORST ↑ IL-1β, TNF-α, iNOS NO and MDA while ↓ GSH and GST in Hippoc and frontal cortex. Catalpol administration suppressed the abnormalities of the above indicators
Guan et al., 2021 [99]	Adult ♂ Kunming mice BW 18–22 g	CUMS- induced depression model	Zhengzhou, Henan, China	Six group of 10: Ctrl group, CUMS group (CUMS + double distilled water i-gastr), CUMS + quercetin (10 mg/kg i-gastr), CUMS + quercetin (20 mg/kg i-gastr), CUMS + quercetin (40 mg/kg i-gastr), CUMS + FH (20 mg/kg i-gastr) for 21 days → behav test (SPT,OFT,FST) → sacrifice → Hippoc tissue collected → WB for PI3K/Akt/Nrf2/HO-1 detection kits for iNOS/NO/SOD/GST/GPx/GSH/MD	CUMS ↓ PI3K/Akt, Nrf2/HO-1in the Hippoc of mice. (all *p* < 0.01); Quercetin (40 mg/Kg) ↑ PI3K/Akt, Nrf2/HO-1	CUMS for 21 days ↓ performance in behav tests (SPT,OFT,FST) quercetin(40 mg/kg) ↑ (SPT,OFT,FST) performance; CUMS ↓ SOD GST (both *p* < 0.01),quercetin at 40 mg/kg ↑ SOD,GST. CUMS ↑MDA, NO, iNOS while quercetin 40 mg/kg ↓ them; quercetin (20 mg/kg) ↑ performance (SPT,OFT,FST) and Akt, SOD, HO-1 and ↓ iNOS/MDA; quercetin (10 mg/kg) no reversal effect on the above indicators
D.-c. Sun et al., 2022 [100]	5–6-wk-old ♂ ICR mice BW 18–22 g	CUMS-induced depression; literature-based prediction	Tianjin, China	Animals were divided into 6 groups (n = 10) including the “normal” (non-stressed mice), the CUMS (CUMS mice administered with distilled water), the venlafaxine (CUMS + venlafaxine), *SE* high-, medium-, and low-dose groups (CUMS + *SE* 1.8, 1.35 and 0.9 g/kg). → FST, TST, and OFT. DA in Hippoc and cerebral cortex, IL-2 and CORST in blood, Nrf2, Keap1, NADP, NQO1 and HO-1 in mice were Measur by ELISA and WB	In the *SE* group: up-regulation of Nrf2, Keap1, NQO1 and HO-1 vs. Ctrl. *SE* (1.8, 1.35 g/kg) ↓ immobility in FST and TST, while *SE* exhibited no significant effect in OFT. Compared with the CUMS group, the *SE* group (0.9 g/kg) showed significant differences in Hippoc and cortical DA levels. The *SE* (1.8 g/kg) group significantly ↓ the activities of CORST (*p* < 0.05) but not serum IL-2 (*p* > 0.05). WB showed ↑ Nrf2, Keap1, NQO1, and HO-1 in the *SE* group (1.8 g/kg)	Compared with the CUMS group, the protein contents of Nrf2, Keap1, NQO1, HO-1 ↑ in the *SE* group; *SE* may enhance antioxidant effects through regulating Nrf2-ARE signalling pathways. *SE* ↑ Nrf2 and downstream anti-OS targets
Cheng et al., 2022 [101]	♂ Sprague Dawley rats; BW 130–150 g	CUMS-induced depression model in rats	Xiamen, China	Rats divided into 4 groups of 10: CUMS group, acupuncture group (acupoints in the skull) fluoxetine group (2.1 mg/kg, i-gastr for 28 days) and Ctrl group. Behav test (OFT, SPT, FST) → sacrificed → Hippoc tissue collected → ELISA for ROS, WB for Nrf2/HO-1, Immunohistochemical for Bcl-2/caspase-3	CUMS↓ Nrf2/HO-1. Acupuncture pretreatment ↑ antioxidant enzymes in the Nrf2 pathway	Acupuncture/fluoxetine ↑ sugar preference SPT and ↓ immobility time in Behav test. Acupuncture ↓ Bax2/caspase-3 and ↑ Bcl-2. Acupuncture improved DLB of CUMS rats. Acupuncture showed AD effects in ↓oxidative stress products regulating the Nrf2/HO-1 pathway
Dang et al., 2021 [102]	♂ C57BL/6J mice (aged 7–8 wk) and retired ♂ CD-1 mice (aged 16–20 wk)	CSDS depression model	Yuzhong District, Chongqing, 400016, China	Behav tests (SI, SPT, OFT, EPM, NOR, TST, FST). Hippoc and mPFC tissues for Nissl staining, immunofluorescence, targeted energy metabolomics analysis, ELISA, Measur of MDA, SOD, GSH, GSH-PX, T-AOC. WB and PCR for Sirt1/Nrf2/HO-1/Gpx4 signalling pathway. EX527, a Sirt1 inhibitor and ML385, an Nrf2 inhibitor 30 min before EDA injection daily	EDA (in CSDS model) ↑ expressions of Sirt1, Nrf2, HO-1 and Gpx4 in the Hip. EX527 and ML385 reversed the effect of EDA	EDA ameliorated CSDS-induced depressive and anxiety-like behaviours (↓ of neuronal loss, microglial activation, astrocyte dysfunction, OS damage, energy metabolism and pro-inflammatory cytokines activation in the Hip and mPFC)
Xia et al., 2022 [103]	♂ C57BL/6 mice (8-wk-old)	DSS-induced IBF-associated depression and anxiety	Yangling, China	Test the effect of 6-wk 100 mg/kg BW/day sesamol for DSS-induced mice who developed IBF after DSS induction; Behav E + M (time spent and % entries in open arms), MBT (total and % travelled distance); TST (immobility time); Tissue Claudin 1, TNF-α, IL-1β, Iba1, and GFAP; Cortical mRNA expression of TLR-4, iNOS, COX-2, TNF-α, IL-1β, and IL-6; MDA, SOD, and GSH content in serum and cortex; Nrf2–HO-1, NQO1, HO-1 and their mRNA expression in cortex; BDNF in Hippoc CA3, mRNA expression of BDNF in cortex and Hippoc, p-TrkB/TrkB, p-CREB/CREB, PSD length and width, PSD-95 in cortex and Hippoc CA3; cortical NA and 5-HT content and mRNA expression of Htr1a and Htr2a, and PSD-95 mRNA expression in Hippoc and cortex; WB for 5-HT1AR F and 5-HT2AR F, Hippoc PSD-95, TLR-4, p-NF-κB/NF-κB, iNOS, COX-2, and p-IKBα/IKBα	Sesamol ↓ TLR-4/NF-κB, ↓ OS, ↑ Nrf2-HO-1 pathway, ↑ BDNF, ↑ BDNF/TrkB/CREB signalling pathway, and ↑ NA and 5-HT levels; ↑ Claudin 1 levels, thus reducing epithelial junction dysfunction, and improved behav test performance. It ↓ TNF-α, IL-1β, ↑ NQO1, ↑ Hippoc, cortical BDNF	Sesamol ↓ inflam, epithelial barrier dysfunction, and DLB and anxiety-like behaviours via the gut–brain axis; it restored synaptic impairment in a DSS-induced IBF mouse model of depression and anxiety and ↓ DLB and anxiety
Li J. et al., 2022 [104]	♂ Sprague Dawley rats; BW 200–250 g	CRS-induced DLB	Beijing, China	Experiment 1: rats divided into 4 groups: CRS, Ctrl, CRS + Escitalopram 1 mg/kg/day i.g. × 28 days, CRS+ Rg1 20 mg/kg/day i.g. × 28 days. → behav tests (SPT, OFT, FST) → sacrifice → Hippoc tissue and serum collected. Experiment 2: rats divided into 4 groups: Ctrl, CRS, CRS + lenti + shNC (injection *in vivo* with lenti-shRNA into lateral ventricle), CRS + lenti + shGAS5 (injection *in vivo* with lenti-shGAS5 into lateral ventricle) → 48h CRS × 28 days → behav tests (SPT, OFT, FST) → sacrifice → Hippoc tissue and serum collected → qPCR for GAS5. Experiment 3: rats divided into 4 group: Ctrl, CRS, CRS + Rg1 (20 mg/Kg/day i.g. 1 h after CRS stimulation) +lenti vector (*in vivo* injection with lenti-shRNA into lateral ventricle), CRS+ Rg1(20/mg/Kg/day i-gastr 1 h after CRS stimulation) + lenti-GAS5 (*in vivo* injection with lenti-shGAS5 into lateral ventricle) → 48 h CRS × 28 days → behav tests (SPT, OFT, FST). → sacrificed → Hippoc tissue collected → qPCR for GAS-5; IF for IBA-1. Experiment 4: Rat microglia cell line HAPI → with LPS, (1μM) or solution × 2 h → treated with Rg1 (5 μM, 10 μM, 20 μM) for 24 h → HAPI WB for COX-2/iNOS/SOCS3. PC-12cell → with CORST (400 μM) or solution for 2 h → treated with Rg1 (5 μM/10 μM/20 μM) for 24 h. Experiment 5: HAPI → GAS5 shRNA after 48 h → LPS (1 μM) × 24 h. HAPI qPCR GAS5, ELISA for TNF-α, IL-1β, IL-6, WB for COX-2/iNOS/SOCS3. PC-12 → GAS5 shRNA after 48 h → CORST (400 μM) × 24 h. PC-12 qPCR for GAS5. Experiment 6: GAS5 KO Nrf2/SOCS3. HAPI/PC-12 → GAS5 shRNA. 48 after → HAPI qPCR, WB for SOCS3. PC-12 qPCR, WB for Nrf2. RIP and RNA pull-down assay → validate binding between GAS5/EZH2. ChIP-qPCR for EZH2/H3K27me3 in the promoter region of SOCS3 in HAPI. **Experiment 7**: SOCS3/NRF2 KO ↓ protective effects of Rg1 in HAPI7 PC-12 *in vitro* model. HAPI → SOCS3 shRNA. 48 h after → LPS (1 μM) for 2 h, → Rg1 (5 μM, 10 μM, 20 μM) × 24 h. ELISA for TNF-α, IL-1β, IL-6 WB for COX2/iNOS/SOCS3. PC-12 → NRF2 shRNA 48 h after → CORST (400 μM) × 2 h →Rg1 (5 μM, 10 μM, 20 μM) × 24 h. IF for IBA-1, WB for Nrf2/SOCS3/EZH2; ELISA for TNF-α/IL-1β/IL-6, TEM for MCh morphology, mitoSOX kit for MCh ROS detection, JC-1 assay to assess MCh membrane potential; ATP assay kit for ATP; q PCR for GAS5. ChIP-qPCR for EZH2/H3K27me3 in Nrf2 promoter region in PC-12	SOCS3/Nrf2 ↓ in CRS group compared to CRS+ Rg1, CRS + Escitalopram and Ctrl. CRS rats showed ↑ EZH2 in Hippoc, ↓ SOCS3/Nrf2, ↑ EZH2 than other groups. GAS5 → ↑ EZH2, ↑ GAS5 in CRS rats compared to other groups. CRS + lenti + shGAS5 → ↑ Nrf2 compared to CRS + lenti + shNC and CRS; Rg1 ↑ ATP, SOCS3/Nrf2 ↓ GAS5 overexpression-related ATP, SOCS3, and Nrf2 reduction. CORST ↓ Nrf2/HO-1; Rg1 treatment ↑ Nrf2/HO-1. CORST stimulation ↓ Nrf2 and HO-1, GAS5 knockdown-related Nrf2 and HO-1 increase. GAS5 knockdown ↓ EZH2 and H3K27me3 enrichment in Nrf2 promoter region in PC-12 cells. GAS5 knockdown ↑ Nrf2 in PC12 and HAPI. In PC-12, Nrf2 knockdown reversed the protective effect of Rg1 on ROS production and on MCh membrane potential	CRS ↓ behav test performance, ↑ Hippoc IBA-1, ↑ Hippoc TNF-α/IL-1β/IL-6, ↓ Hippoc ATP and ↑ MCh injury compared to CRS + Rg1, CRS + Escitalopram and Ctrl. CRS, CRS + lenti + shNC ↑ Hippoc GAS5 compared to CRS + lenti + shGAS5 and Ctrl. ↑ performance on OFT and FST in CRS + lenti + shGAS5 and Ctrl compared to other groups. SFT ↑ performance in CRS + lenti + shGAS5 and Ctrl > than in other groups. GAS5 knockdown ↓ CRS-induced DLB. CRS + lenti + shGAS5 ↓ Hippoc IBA-1 compared to other groups. ↓ serum and Hippoc TNF-α, IL-1β, and IL-6 levels in Ctrl and CRS + lenti + shGAS5 groups compared to other groups. ↓ Hippoc ATP in CRS than in CRS + lenti + shNC, CRS + lenti + shGAS5, and Ctrl. ↑ MCh injury in CRS compared to CRS + lenti + shGAS5; GAS5 knockdown did not affect EZH2 expression. ↑ Hippoc GAS5 5 in CRS than CRS + Rg1 + lenti; Rg1 ↓ DLB, ↓ IBA-1, ↓ microglial activation, and ↓ MCh dysfunction; ↑ GAS5 overexpression → partly reversed the effect of Rg1 on DLB, on microglial activation, on IBA-1, and on MCh dysfunction. GAS5 overexpression → ↑ IBA-1, LPS ↑ TNF-α, IL-1β, and IL-6; Rg1 treatment ↓ TNF-α, IL-1β, no significant effect on IL-6. LPS ↑ COX-2/iNOS and ↓ SOCS3 in HAPI. Rg1 treatment ↓ MCh dysfunction. GAS5 inhibition exhibited a protective effect, similar to that of Rg1 treatment
Muhammad et al., 2022 [105]	♂ Sprague Dawley rats; BW 180–200 g	LPS-induced DLB	Islamabad, Pakistan	Rats divided into 2 groups of 10, one treated with fluoxetine (5 mg/kg) + LPS, CAR20 or CAR50 + LPS → single dose of each × 5 days, ip + LPS → or single dose or after CAR20/50 (3rd, 4th day 1 mg/kg), and another, divided into 3 subgroupings (10 animals/group): ATRA + LPS, ATRA + LPS + CAR20/50, ATRA + LPS+ fluoxetine. ATRA ip injection 30 min before LPS. At Day 2, → behav tests (FST, LDB, E + M, sucrose SPIT) → sacrificed → Hippoc and cortical tissue collected and processed to RT-PCR and ELISA for Nrf2/HO-1. Immunohistochemical analysis for OS-related molecules	LPS ↓ Nrf2/HO-1 expression in cortex and Hippoc compared to Ctrl (*p* < 0.05). CAR20/50 + LPS ↑ Nrf2 and HO-1 compared to the LPS group (*p* < 0.01)	LPS ↑ ROS and DLB. CAR20/50 ↓ DLB and↑ Nrf2/HO-1; no differences between CAR20/50 + ATRA + LPS and ATRA + LPS
Shen et al., 2022 [106]	♂ C57BL/6J mice (2 months old, BW 20–25 g)	LPS-induced CUMS and DLB	Hefei, Anhui, China	Experiment 1, mice randomised into 5 groups of 6: Ctrl, 2 h post-LPS, 6 h post-LPS, 12 h post-LPS, and 24 h post-LPS. After LPS injection → mice sacrificed → Hippoc tissue collected. Experiment 2, mice randomised into 6 groups of 6: Ctrl, LPS, LPS + PSP, LPS + fluoxetine, (30 mg/kg ip), LPS + calpeptin (2 mg/kg calpeptin ip), and LPS+ MCC (50 mg/kg ip). 24 h after LPS injection → sacrificed → Hippoc tissue collected. Experiment 3, mice randomised into 3 groups of 6: Ctrl, LPS, and LPS + NAC. 24 h after LPS injection → sacrificed → Hippoc tissue collected. Mice further randomised into 4 groups: Ctrl, CUMS, CUMS + PSP (i-gastr × 21 days 400 mg/kg) and CUMS + MCC (5 mg/kg ip × 7 days) After 21 day → behav tests (TST, SPT, FST, OFT) → sacrificed → Hippoc tissue collected. Hippoc tissue WB for OS-related molecules and IF for Nrf2	LPS ↓ Nrf2 expression compared to Ctrl in Hippoc CA1 region (*p* < 0.05). Treatment with PSP improved LPS-mediated ↓ in Nrf2 expression in CA1 (*p* < 0.05). LPS+ PSP ↑ Nrf2 compared to Ctrl (*p* < 0.05)	LPS treatment ↑ calpain-1 expression and substrate degradation, activated the NLRP3 inflammasome signalling pathway and glial cells, and Nrf2 and calpastatin expression. Treatment with PSP prevented these LPS-induced changes. Nrf2 expression was ↓ in the hippocampi of animals treated with LPS or subjected to CUMS, and this decrease was prevented by PSP
Jiang et al., 2022 [107]	6–8–wk-old ♂ C57BL/6J mice (*n* = 60, BW 20–22 g); and 12-month-old retired breeders ♂ CD-1 mice (*n* = 70, BW 20–22 g)	CSDS-induced DLB	Beijing, Changsha, Luzhou, China	After a 1-wk adaptation, mice randomised into Ctrl, model, and treatment groups IMI (15 mg/kg ip), and Rb1 (35 and 70 mg/kg ip). Except for Ctrl, all animals subjected to CSDS treatment → behav testing (SIT, OFT, TST, FST) over the next 4 wk. IMI p.o. × 32 days. Hippoc protein concentration, CAT, SOD, and LPO Measur with specific kits. Pro-inflammatory mediator (TNF-α, IL-18, IL-1β) serum levels Measur with ELISA. WB for SIRT1, NLRP3, cleaved Caspase-1, ASC, IL-1β, HO-1, Nrf2, and β-actin	Nrf2 and HO-1 expression substantially ↓ in rat CSDS Hippoc compared to Ctrl. 70 mg/kg Rb1 ↑ Nrf2 expression	Rb1 treatment can rescue DLB-like social avoidance and behav despair of CSDS-induced mice. Rb1 attenuates pro-inflammatory cytokine production and inhibits the activation of NLRP3 inflammasome. Furthermore, Rb1 normalised OS following Nrf2/HO-1 and SIRT1 activation. Findings show that Rb1 attenuates NI
de Souza et al., 2022 [108]	♂ adult Swiss mice BW 25–30 g	CUMS-induced depression	Fortaleza, Ceará, Brazil	Mice were exposed to a variety of stressful events (restraint, tilted cage, intermittent circle between lights on and off, constant light, water deprivation, wet cage, electric shock) × 28 days; from the 14th day they received DMF 50 and 100 mg/kg or fluoxetine 10 mg/kg or plac. On the 29th day → behav tests (OFT, FST, SPT, NOR). Mice divided into two groups, Ctrl and CUMS, which were subdivided into plac (carboxymethyl cellulose 0.5%), DMF50, DMF100 or fluoxetine 10 mg/kg (FLU) groups. 14 days after the beginning of the procedure, treatments were started in all groups, 30 min before daily stress application in CUMS groups. Simultaneously, Ctrl received the same treatments. Immunoenzymatic assay for TNF-α and IL-1β concentrations in Hippoc. IF to analyse subfields CA1, CA3, and DG	MMF (active metabolite of DMF) showed an efficient binding interaction with the Keap1 protein, leading to Nrf2 activation	DMF is a promising prodrug for the treatment of MDD and for comorbidities such as memory deficit. DMF modulated the neuroinflammatory pathway, ↑ astrocyte expression, ↓ microglia expression and the production of the pro-inflammatory cytokines IL-1β and TNF-α. MMF bound the Keap1 protein → activated Nrf2; it also bound HCAR2 protein, leading to a complex signalling cascade cross-talking with the Nrf2 pathway. It is suggested that one of the mechanisms involved in the AD effect of DMF is NI suppression, triggered by the binding of its metabolite MMF to the HCAR2 protein
Fasakin et al., 2022 [109]	Adult ♂ Wistar Albino rats, BW 181 ± 13 g	Depression and anxiety linked to NI and OS.	Akure, Nigeria	Six rats randomly housed in each cage: Group 1 = normal Ctrl. Groups 2 to 5 = CSAE once daily (doses of 5, 50, 500, and 2000 mg/kg BW, respectively). Groups 6 to 9 = DSAE once daily (doses of 5, 50, 500, and 2000 mg/kg BW, respectively). Groups 10 to 13 = NTAE once daily (doses of 5, 50, 500, and 2000 mg/kg BW, respectively). Groups 14 to 17 = CMAE once daily (doses 5, 50, 500, and 2000 mg/kg BW, respectively). Behav Tests: E + M, FST, TST. 91 days after alkaloid administration, rt-qPCR on Hippoc tissue. Biochemical analyses were performed to assess MAO activity, dopamine concentration, ACE activity, AChE activity, GDH activity, ROS concentrations. Concentrations of TNF-α, IL-1β, and IL-10 were evaluated using ELISA. Gas chromatograph–mass spectrometry analysis of alkaloid-rich extracts was performed	CSAE, NTAE, and CMAE ↑ 5-HT, BDNF, CREB and Nrf2 while ↓ NF-ĸB, GSK3β JNK3 and nesfatin-1 at 5, 50 and 500 mg/kg BW sub-chronic exposure. However, DSAE sub-chronic administration ↓ serotonin, BDNF, CREB and Nrf2 while ↑ NF-ĸB, GSK3β and JNK3	This study established the AD and anxiolytic potentials of CSAE, CMAE, and NTAE via improving monoaminergic bioavailability, activation of neurotrophic signalling cascades and de-hyper-activation of hypothalamic–pituitary–adrenal axis. The difference between the effects of CSAE, CMAE, and NTAE at low and high doses may be the underlying factor in the discrepancies between the results of studies that have evaluated depression and psychoactive substance use
Tan et al., 2022 [110]	Adult ♂ C57BL/6 mice (BW 20 ± 2 g)	Behav despair mouse model *in vivo* and H_2_O_2_-induced PC12 cell model *in vitro*	Shenyang, 110016, China	Mice randomised into 4 groups of 10: plac, Fluoxetine 15 mg/kg, i.g.), SEO 250 group (250 mg/kg, i.g.) and SEO 750 group (750 mg/kg, i.g.). Flu and SEO suspended in 0.5% CMC-Na, plac 0.5% CMC-Na. Treatment once daily × 9 consecutive days. Behav tests (FST, TST). Serum, liver, brain, and cell supernatant MDA, SOD, CAT, and GSH levels detected through ELISA. Hippoc, cortex, and cell supernatant processed through WB (Nrf2, HO-1,PI3K,p-PI3K,p-Akt, GSK3β, p-GSK3β, LaminB, β-actin). Hippoc and cortical histopathology. PC12 cell culture. Groups: Ctrl, plac (300 μmol/L H_2_O_2_), SEO (0.78–25 μg/mL + 300 μmol/L H_2_O_2_). IF for PC12 cells with anti-Nrf2. 18 batches of SEO analysed by GC-MS. DPPH and ABTS assay	After FST and TST, expressions of nuclear Nrf2 in Hippoc and cortex significantly ↓. SEO treatment could reverse these changes, which indicates that SEO might promote Nrf2 translocation to reduce OS	SEO showed a promising AD-like effect in mice; the mechanisms of this effect might contribute to the antioxidant activities of SEO. SEO could ↑ Nrf2/HO-1 pathway to improve OS status and exert AD-like effects. PI3K/Akt/GSK3β signalling pathway might also be involved
Wu et al., 2022 [111]	Adult ♂ Sprague Dawley rats (BW 230–250 g, 6–7-wk-old)	CUMS-induced depression	Zhengzhou, Henan, China	After 7 days of adaptive life, all rats were evaluated by behav tests (OFT, FST and SPT. On the next day, rats were randomly divided into five groups: (1) Ctrl group (n = 10); (2) CUMS group (n = 10); (3) CUMS plus Catalpol group (n = 10); (4) CUMS plus ZnPP group (n = 6); (5) CUMS plus ZnPP + Catalpol group (n = 6). CUMS lasted for 3 wk. Catalpol was administered orally (dose of 10 mg/kg and volume of 20 mL/kg) for 3 wk. ZnPP injected into the intracerebroventricular (dose of 10 μg/rat and volume of 5 μL/rat) for 3 wk. 24 h after the last drug intervention, behav. tests were performed. Biochemical analyses were performed to determine Hippoc total protein and the levels of iNOS, GPX and GST in the Hippoc (using catalytic reaction plus chemical colourimetry method and the levels of NO, MDA, SOD, and GSH (nitrate reductase method, barbituric acid sulfate method, hydroxylamine method, and dithiodinitrobenzoic acid method, respectively)	CUMS exposure ↓ the phosphorylation level of ERK1/2, the nuclear expression level of Nrf2, and the total intracellular levels of HO-1, SOD, GPX, GST, and GSH and ↑ the level of peroxide MDA. Before intervention by HO-1′s antagonist ZnPP, catalpol significantly reversed these abnormalities. However, after intervention by HO-1′s antagonist ZnPP, the reversal effects of oral administration catalpol on the phosphorylation level of ERK1/2, the nuclear expression level of Nrf2, and the total intracellular levels of HO-1, SOD, GPX, GST, GSH, and MDA were completely abolished, which implied that HO-1′s antagonist ZnPP may abolish the regulation of catalpol on antioxidant and peroxide related factors in the Hippoc of CUMS-induced DLB rats	The AD-like effects of catalpol might be due to HO-1 that up-regulates the ERK1/2/Nrf2/HO-1 pathway-related factors to enhance the antioxidant defence, triggering the down-regulation of the COX-2/iNOS/NO pathway-related factors to inhibit NI, and the up-regulation of the BDNF/TrkB pathway to enhance neurotrophic effects
Zhao et al., 2022 [112]	♂ C57BL/6 mice (8-wk-old)	CP-induced anxiety and DBL	Zhanjiang, Guangdong, China	Mice received CP ip × 2 wk and TSP × 4 wk → behav, biochemical, and molecular tests conducted	Protein expression of Keap1 ↑, Nrf2 and HO-1 ↓ in CP compared with Ctrl. TSP ↑ Nrf2 and HO-1	TSP ↓ behav test abnormalities, ↓ SOD, MDA, and GSP abnormalities; ↓ expression of IBA-1, TNF-α and IL-1β, ↓ Hippoc apoptosis in CA1 and CA3 subfields; ↑ Nrf2/HO-1 signalling pathway; ↑ BDNF/TrkB/CREB signalling pathway, and ↓ the Bcl-2/BAX/caspase-3 apoptosis pathway
Bansal et al., 2022 [113]	3-month-old ♂ BALB/c mice (BW 25–30 g)	OBX-induced depression	Chandigarh, Panjab, India	Thirty mice randomised in 3 groups of 10. I group = sham; II = OBX + plac plac; III and IV groups = OBX + Ro61-8048 (0.2 and 0.4 mg/kg) × 14 days → behav tests (OFT, SFT, SPT), biochemical analysis, ELISA (Nrf2, BDNF, NF-κB, TNF-α and IFN-γ), HPLC (5-HT, 5-HIAA, TRP, and KYN levels), SF(QA) and qPCR (Hippoc and cortex based gene expression analysis) were performed	KMO inhibition with higher dose of Ro61-8048 (0.4 mg/kg) in OBX mice showed: ↓ CORST levels; ↑ Nrf2 and GCLC with no significant effect on HO-1, ↑ PI3K and Akt, ↓ GSK3β gene expression and ↑ GFAP expression, ↓ MDA and nitrite, ↓ NF-κB and IFN-γ levels with no effect on TNF-α, ↑ BDNF, ↑ TRP, 5-HT and 5-HIAA, ↓ QA. The lower dose of Ro61-8048 (0.2 mg/kg) showed no significant effect	OBX ↑ OS in the brain; Ro61-8048 ↓ the effect of KMO and ↓ OS. KMO inhibition and QA-associated down-regulation ↓ Keap-1 and ↑ Nrf2 expression
Yu et al., 2022 [114]	♂ C57BL/6 mice (6–8 wk, BW 20 ± 2 g)	LPS-induced DLB	Fuzhou, Fujian, China	Mice were divided into 4 groups (9 each): CON, the Ctrl group; CON + RA (80 mg/kg); LPS (0.1 mg/kg); LPS + RA (80 mg/kg). Behav tests were carried out 24 h later → mice sacrificed, and brains used for IF, histopathological analysis, WB, and rt-qPCR	Nrf2 ↓ after LPS injection compared to Ctrl, RA administration attenuated the ↓ of Nrf2	RA improved behav test performances and BW; ↓ histologic brain damage in LPS-exposed mice; blocked the ↓ of BDNF (rt-qPCR and WB) and of Nrf2 (WB). LPS ↓ LC3 (IF), ↓ p21 and p62, the levels of which were restored by RA. ME1, IDH1, and 6-PGDH mRNA were ↓ in the LPS group and ↑ after RA. RA inhibited the LPS-induced ↑of CD44, iNOS, TNFα, and IL-1β mRNA expression
Kang et al., 2022 [115]	♂ C57BL/6J mice (8-wk-old, BW 21–23 g, n = 36)	CRS-induced DLB	Daejeon, South Korea	Mice divided into 4 groups of 9: normal; CRS (stress and distilled water), PSE (stress and 100 mg/kg PSE), and positive Ctrl (stress and 100 mg/kg NAC). Behav tests (OFT, FST, TST); murine microglial cells (BV2) and Hippoc neuronal cells (HT22) were cultured for cytotoxicity, cytokines, and phagocytosis assays. IF and WB were also performed	p-Nrf2 ↑ with PSE (*p* < 0.05 for 40 μg/mL). PSE pretreatment increased the level of HO-1 protein expression (*p* < 0.05 for the 10 and 20 μg/mL doses). NAC exerted similar effects as PSE	PSE (10, 20, and 40 μg/mL) attenuated the ↑ in inflammatory factors (NO and TNF-α), translocation of NF-κB, and phenotypic transformations in a dose-dependent manner. These inhibitory effects of PSE on microglia were supported by its regulatory effects on the CX_3_CR1/Nrf2 pathway. PSE (100 mg/kg) ↓ sickness, anxiety, and DLB in mice subjected to CRS and improved behav test performance
Wang et al., 2022 [116]	40 ♂ ICR 7-wk-old mice	PTZ-induced seizures and depression	Liaocheng, western Shandong, China	Mice divided into 4 groups of 10: Ctrl, PTZ, PTZ + EPA, and PTZ + DHA groups. Behav tests (OFT; TST; FST). Hippoc DHA, EPA and neurotransmitter levels were detected by GC. Histopathology of Hippoc was conducted through H&E-staining, FJB-staining and IF. Hippoc proteins were analysed by WB	The protein expressions of p-Nrf2 and HO-1 were significantly ↓ in the PTZ group compared with Ctrl. EPA and DHA significantly ↑ p-Nrf2 and HO-1 (higher levels in EPA) group	↓ GSH, ↓ SOD activity, ↑ MDA, ↓ GPx4, xCT, HO-1, and p-Nrf2 in the PTZ group, reversed by EPA and DHA. EPA and DHA improved behav test results; ↑ GABA and GABARA1A levels without changing Glu levels; improved the morphology of cell damage (EPA repaired > than DHA); ↓ Hippoc total Fe (which was ↑ in the PTZ group) and ameliorated iron-homoeostasis-related proteins; ↓ pro-IL-1*β*, IL-1𝛽, and TNF𝛼 (which were ↑ in the PTZ group); EPA inhibited NLRP3 and ↓ Caspase-1 levels; DHA ↓ ASC, pro-Caspase-1 and Caspase-1
Nasehi et al., 2022 [117]	Adult ♂ mice, sons of pregnant mothers from Pasteur Institute, Tehran, Iran (Balb/c?), fed ad libitum and kept in 12 h/12 h dark/light cycles	Maternal separation-induced depression associated with CVD	Zanjan, Iran	Depression was induced with 3 h daily separation from mothers while weaning; mice with maternal separation-induced depression and CV comorbidities treated with fluoxetine or SAHA + fluoxetine ip →behav tests (OFT [horizontal and vertical locomotion], E + M (Y-maze), TST, FST, SPlT [grooming]) + gene analysis with RT- qPCR; content and gene expression of PPAR-𝛼, NOX-2, -4, PGC-1α, and Nrf2	SAHA ↓the *NOX-4* gene expression level in mice treated with SAHA + fluoxetine without significantly changing *NOX-2* expression. SAHA ↑ PGC-1α and Nrf2 in heart tissues of maternally separated mice, but had no effect on *PPAR-𝛼* gene expression. Maternal separation induced depressive behaviour on all behav tests. SAHA and fluoxetine had no effect on OFT locomotion, but ↑ time spent in open arm of the Y-maze, and ↓ immobility time on FST and TST	SAHA reversed DLB and restored mitochondrial function in the heart via an effect on NOX-4 and mitochondrial biogenesis gene (PGC-1α and Nrf-2) expression levels. The authors speculated that when fluoxetine lacks effectiveness on cardiac mitochondrial biogenesis and cardiac *NOX-4* gene expression, patients with depression may develop a recurrence of their depression
Pei et al., 2022 [118]	50 ♂ Balb/c mice (BW = 25 g)	LPS-induced depression	Changchun, Jilin, China	Mice were divided into 5 groups (n = 10 each): Ctrl, LPS-induced, palmatine (50 and 100 mg/kg) group, and fluoxetine groups. Each group except Ctrl received LPS (5 g/L, 3 μL/mice). OFT and EPM were performed; SOD, TNFα, IL1β, and IL6 detected with ELISA; apoptosis and ROS with flow cytometry; total Hippoc proteins analysed with WB (BAX, Bcl-2, Nrf2, HO-1, β-actin)	Nrf2 and HO-1 in the LPS-induced group were ↓ compared with the Ctrl group. After palmatine treatment, their protein expression levels were significantly ↑	Palmitine ameliorated mitochondrial damage, improved behav tests, ↓ levels of SOD, TNF-α, IL-1β, and IL-6. It also ↓ neuronal apoptosis in the Hippoc, and depression through BAX/Bcl-2 and Nrf2/HO-1 signalling pathways
J.Y. Sun et al., 2022 [119]	60 ICR ♂ mice (6–8-wk, BW 18–22 g)	CORT-induced depression	Chengdu, Sichuan, China	All mice were randomly divided into 6 groups (n = 6): Ctrl group, CORT group, Fluoxetine (3 mg/kg), EOP low-dose group (1 mg/kg), EOP medium-dose group (2 mg/kg) and EOP high-dose group (3 mg/kg). Except for the Ctrl group, mice in other groups were injected with CORT 20 mg/kg ip daily for 4 consecutive wk→behav tests (TST, FST, SPT, OFT)+ blood samples and brain tissues for analysis	EOP ↓ CORT-induced DLB in mice (by improving behav tests), ↓ CRH, ACTH and cortisol in the brain tissues, ↑the phosphorylation of PI3K and Akt in Hippoc neurons and PC12 cells, ↑ Nrf2 and ↓ the CORT-induced OS. It also ↓ CORT-induced Hippoc neuron injury and apoptosis and ↑ the proliferation ability and cell viability of PC12 cells	EOP had a significant AD effect on the symptoms of CORT-induced depression in mice. EOP exerted anti-apoptotic effects on Hippoc neurons through PI3K/Akt/Nrf2 signalling pathway
He et al., 2022 [120]	♂ adult C57BL/6 mice (8-wk-old, BW 20–25 g), CD-1 mice (14-wk-old, BW 40–45 g), ♂ adult YFP-Nrf2 WT mice, and YFP-Nrf2 KO mice	CSDS-induced DLB	Guangzhou, Guangdong, China	For the CSDS procedure, C57BL/6 mice or YFP mice were defeated by CD1 mice (10 min × 10 days). SIT performed to select susceptible mice (70%) → locomotion test, FST, and SPT. TREM2-HDO (icv) was injected on day 0, SFN (ip) 30 min before the CSDS and LPS. CSDS-susceptible mice were used for luciferase assay (lateral habenular BV2 cultured microglia cells treated with SFN or siRNA-Nrf2), ChIP assay (with Nrf2 antibody), qPCR (levels of TREM2 promoter mRNA and Trem2 mRNA); ImBl (TREM2, Nrf2, BDNF, arginase, phospho-TrkB, PSD-95, TrkB, β-actin antibody, β-tubulin antibody, GAPDH), IF (anti-TREM2, anti-IBA-1, anti-arginase1 [microglial anti-inflammatory phenotype marker]), dendritic spine analysis	SFN → ↑ Nrf2 → ↑ microglial arginase 1+ phenotype by initiating TREM2 transcription in mPFC, improving DLB in CSDS mice. Nrf2 KO ↓ TREM2 and microglial arginase 1+; both → DBL. Nrf2 KO and ↓ TREM2 expression associated with ↓ BDNF/TrkB signalling pathway	Luciferase assay showed that Nrf2 regulates TREM2 transcription; qPCR showed that ↑ SFN and ↑ Nrf2 → ↑ TREM2 promoter activity and ↑ TREM2 mRNA (which were ↓ in CSDS-mouse mPFC). ImBl and qPCR → TREM2-HDO dose-dependently decreased mRNA and protein expression of both TREM2 and Nrf2. IF showed TREM2 to colocalise with IBA1 and arginase 1+ in microglial cells. LPS ↓ TREM2 and arginase 1 and ↑ IBA1; these effects were reversed by SFN. qPCR → reversal by SFN of the reduction in the anti-inflammatory cytokines IL-4 and IL-10 in the mPFC of CSDS mice. ImBl → reversal by SFN of the reduction in Nrf2, TREM2, and arginase 1 expression in the mPFC of CSDS mice. Nrf2 KO ↓ TREM2 and arginase 1+ microglial phenotypes in mPFC
Samy et al., 2022 [121]	10 wk-old ♁ Balb/c mice (BW = 25 g)	Post-OVX depression	Alexandria, Egypt	Eighty mice assigned to 5 groups (n = 16 each) receiving treatments or plac × 3 wk: Ctrl (sham-operated + plac); OVX mice + CarA (20 mg/kg/day, p.o.); OVX+ SnPP-IX (50 μmol/kg/day i.p); combined-treatment OVX + CarA + SnPP-IX received CarA; Behav tests. HO-1 activity and brain oxidative and antioxidant markers were determined. Isolation of Hippoc and cortex RNA for RT-qPCR; protein extraction for WB; determination of brain 5-HT levels through HPLC	↓ Nrf2, Trx-1, and BDNF in OVX mouse brains compared with Ctrl. ↑ Nrf2, Trx-1, and BDNF with CarA either solely or combined with SnPP-IX (except for BDNF, which ↓ in the treatment combination group). Mice treated with SnPP-IX alone did not show any significant impact on Hippoc or cortical Nrf2, Trx-1, or BDNF	Improvement of FST, TST, SPT with CarA and partial improvement with CarA + SnPP-IX. ↓ Nrf2, Trx-1, and BDNF in OVX mouse brain, ↑ by CarA. ↑ in MDA and ↓ in GSH and SOD in OVX mice, ↑ GSH, SOD activity and ↓ MDA in CarA group (antioxidant effect attenuated in CarA + SnPP-IX). ↑ OS, ↑ MDA, ↓ GSH and SOD with SnPPIX alone. CarA ↑ 5-HT level in mouse Hippoc and PFC. Significant thinning of the pyramidal and granule cell layers of the HippP and DG of OVX mice, which was restored to the original thickness by CarA
Si et al., 2023 [122]	♂ 6–8-wk-old Sprague Dawley rats (BW 160–180 g, n = 70)	CUMS-induced DLB	Wuhan, Hubei, China	Rats sorted into 2 groups of 35, Ctrl and CUMS. Behav tests performed to select CUMS-susceptible rats (SUS). SUS randomly divided into 2 groups: CUMS-SS (1 SUS + 3 Ctrl) and CUMS-SI (1 SUS). After 4 wk of SI or SS, the rats in different groups underwent behav tests. Total RNA was isolated for RT-PCR and total protein for WB. IF performed to observe Hippoc astrocyte changes	mRNA expression of Nrf2, HO-1 and NQO1 ↓in CUMS-SI and significantly lower in the CUMS-SI group compared to CUMS-SS	SUS exhibited DLB, memory deficits and social withdrawal. qRT-PCR and WB analysis showed ↓ Nrf2, HO-1, NQO1 and p-ERK in SUS and ↑ Keap1. IF showed ↓GFAP+ astrocytes in SUS. SI after CUMS perpetuated DLB, memory deficits and social withdrawal (overall worsening in behav tests and no BW ↑); ERK/Keap1/Nrf2 signalling was suppressed in CUMS-SI (↓ Nrf2, ERK, and ↑ Keap1 compared to CUMS-SS)
Wang et al., 2023 [123]	♂ 8–10-wk-old adult C57BL6 mice	CUMS-induced DLB	Cangzhou, Hebei, China	13 mice per group: Ctrl, exposed to CUMS + plac, CUMS + PD 100 mg/kg/day delivered by oral gavage, CUMS + PD 200 mg/kg/day oral gavage. → SPT, FST, TST, SIT for depression and MBI, E + M for anxiety → killing → Hippoc neuronal culture and staining, IF for Iba-1 and GFAP in Hippoc CA1, CA3, and DG, rt-qPCR for *Iba-1*, *Gfap*, *Gapdh*, *Hmox1*, *Nqo1*, and *Nrf2* forward and reverse primers, WB for synaptophysin and PSD-95, ELISA for DA, 5-HT, IL-1β, IL-6, and TNF-α	CUMS → ↓ BW, in SPT ↓ sucrose preference, in FST and TST ↑ immobility time, in SIT ↓ affiliation, sociability, memory, and novelty; all these effects were counteracted by oral PD 200 mg/kg/day; CUMS ↑ marble burying in the MBI and time on closed arms in E + M, both counteracted by PD 200 mg/kg/day. PD ↑ PSD-95 and synaptophysin content in Hippoc, dendrite length and number. CUMS ↑ NI markers Iba-1 and GFAP, IL-1β, IL-6, and TNFα in Hippoc, ↑ release and nuclear translocation of NFκB, ↑ ROS, SOD, GPx, ↓ Hmox1, Nqo1, and Nrf2 levels; all these CUMS effects were counteracted by PD	PD reverts changes induced by CUMS on depressive and anxiety-related behaviour; PD restored all changes induced by CUMS and hippocampal neuronal function
Smaniotto et al., 2023 [124]	*Swiss* ♂ mice (BW 25–35 g)	CUMS-induced DLB	Pelotas, Rio Grande do Sul, Brazil	CUMS × 28 days → intranasal IL-4 vs. plac; 8 mice per group, 4 groups (Ctrl, Ctrl–IL-4, CUMS, CUMS–IL-4) subjected to water/food/movement restriction, tail pressure, and inclined box with wet bedding; → OFT, TST, SPlT → killing, tissue extraction and analyses (PFC and Hippoc, adrenals, lymph nodes, thymus, spleen [organs], blood to determine ROS, IDO, CAT, SOD, LPO, MDA, NO metabolites, gene expression, CORST levels)	In OFT no differences in crossings and rearings among groups; in TST, CUMS ↑ immobility and IL-4 reversed it; in the SPlT, CUMS ↓ grooming and IL-4 reversed it. CUMS → ↑ CORST and lymph organs, ↑ NF-*κ*B expression, IL-4 → ↓ CORST and organ weight; ↓ NF-*κ*B expression; in both PFC and Hippoc, CUMS → ↑ IDO, IL-1β, NF-*κ*B, Nrf2, ROS, SOD, and CAT and ↓ BDNF (no effect on LPO in Hippoc and ↑ in PFC; similar effects for MDA, but no effect on NO metabolites in both PFC and Hippoc); all CUMS effects were reversed by IL-4	IL-4 counteracts CUMS-induced DLB; the effects of IL-4 are most evident in the PFC. IL-4 protects against chronic stress-induced depressive behaviour through ↓ of NI and OS
Zhang et al., 2023 [125]	♂ 7–8-wk-old adult C57BL6 mice	LPS-induced depression and anxiety	Guiyang, Guizhou, China	Mice randomised into Plac (Ctrl), to the phenolic glycoside gastrodin (major component of *Gastrodia elata*) 25, 50, or 100 mg/kg/day) or minocycline (microglial inhibitor) 50 mg/kg/day i.p. × 3 days → Plac (Ctrl), gastrodin 25, 50, or 100 mg/kg/day + Plac or LPS 0.25 mg/day, or minocycline 50 mg/kg/day + Plac or LPS 0.25 mg/day i.p. × 10 days; gastrodin-receiving animals were also treated with Nrf2 antagonist ML385 30 mg/kg/day i.p. × 10 days. Network pharmacology analysis, molecular docking on PPAR-γ, STAT6, and Nrf2; → OFT, SPT, E + M, FST → killing, cultured neonatal microglia, Hippoc microglia, rt-qPCR for *Iba-1*, *β-actin*, *Nlrp3*, *iNOS*, *IL-1β*, *IL-10*, *TNF-α*, *Cd11b*, *Cd68*, and *Arg-1*^+^; ELISA for IL-1β, IL-6, IL-10, TNF-α, and NO; WB for Nrf2, pNrf2, and GAPDH; IF for Hippoc Iba-1, iNOS, Arg-1, Nrf2	LPS ↓ sucrose preference in SPT, ↑ immobility time in FST, ↓ number of entries or time of stay in open arms in E + M, ↓ time of stay in central area in OFT; all DLB and anxiety behav reversed by higher doses of gastrodin and minocycline; LPS ↑ Iba-1 staining in Hippoc microglia, thickened cell-body and shortened cell processes; LPS ↑ CD11b, CD86, NLRP3, iNOS; gastrodin 100 mg/kg/day and minocycline reversed NI-induced changes. Gastrodin 100 mg/kg/day or minocycline ↑ the microglial anti-inflammatory marker Arg-1 and Hippoc Arg-1^+^ microglia in LPS-treated, while they ↓ IL-1β and IL-6 and ↑ IL-10. ML385 blocked the effect of gastrodin on ↑ Arg-1^+^ cell proportion in Hippoc microglia and the subsequent ↑ of Nrf2, pNrf2 and nuclear Nrf2 translocation	Gastrodin through Nrf2 prevents depressive and anxiety behaviours through counteracting NI actions. Gastrodin could represent a useful addition to the treatment of depressive and anxiety disorders, as it promptly crosses the BBB

***Note.*** *Abbreviations:* Ab(s), antibody(ies); ABTS, 2,2′-azino-bis-3-ethylbenzothiazoline-6-sulfonic acid; ACE Angiotensin-converting enzyme; AChE, Acetylcholinesterase; ACMS, adipose-derived mesenchymal stem cell; AD, antidepressant; ADSC, adipose-derived mesenchymal stem cells; AI, Artificial Intelligence, Akt, Protein kinase B; AOEs, antioxidant enzymes; ARE, antioxidant response elements; ARS, acute restraint stress ASC, apoptosis-associated speck-like; AST, astrocytes; ATP, adenosine triphosphate; ATRA, all-trans retinoic acids; BAFF, B-cell-activating factor of the tumour-necrosis-factor family, B-cell activating factor; BAX, Bcl-2-associated X protein, bcl-2-like protein 4; BBB, blood–brain barrier; Bcl-2, Bcl-2, B-cell lymphoma 2; BDNF, brain-derived neurotrophic factor; behav, behavioural; bEnd.3, brain endothelial cell line 3; Bilat, bilateral;BL, baseline; BTS, Bangpungtongsung-san; BW, body weight; *C. elegans*, *Caenorhabditis elegans*, a common worm (nematode); CA, concentrated PM2.5 air; cAMP, cyclic adenosine monophosphate; CarA, carnosic acid; CAR20, carveol 20 mg/kg; CAR50, carveol 50 mg/kg; CAT, catalase; ChIP, Chromatin immunoprecipitation; CLA, conjugated linoleic acid; CMAE, *Carica papaya*; CMC-Na, Sodium carboxymethylcellulose; CMI, 3-[(4-chlorophenyl) selanyl]-1-methyl-1H-indole; CMS, Chronic mild stress; cncs, concussions; CORT model, elevated corticosteroid anxiety/depression mouse model; CORST, corticosterone; CP, cyclophosphamide; CRS, chronic restraint stress; CREB, cyclic AMP responsive element binding protein; CRS, chronic restraint stress; CSAE, Cannabis sativa; CSDS, chronic social defeat stress; Ctrl, control(s); CUMS, chronic unpredictable mild stress; CUR, Curcumine; CuZnSOD, copper zinc superoxide dismutase; CVD, cardiovascular disease; CX_3_CR1, CX_3_C chemokine receptor 1; DA, dopamine; DG, dentate gyrus; D-GalN, D-galactosamine; DHA, docosaheaxaenoic acid; 7,8-DHF, 7,8-Dihydroxyflavone; DLB, depressive-like behaviour; DM, diabetes mellitus; DMF, Dimethyl fumarate; DOPAC, 3,4-dihydroxyphenylacetic acid; DPPH, 2,2-diphenyl-1-picrylhydrazyl; DSAE, Datura stramonium; DSS, dextran sulfate sodium; EGR1–4, early growth response proteins 1–4; ELISA, enzyme-linked immune sorbent test; EPA, eicosapentaenoic acid; ERK, extracellular signal-regulated protein kinase; EZH2, Enhancer of zeste homolog 2; E + M, elevated plus (or X or Y) maze; EZM, elevated zero maze; Fen, fenretinide; FiA, filtered air; FJB, Fluoro-Jade B; FJC+, Fluoro-Jade C-positive cells; FO, fish oil; FST, forced swimming test (Porsolt); GABA, γ-hydroxybutyric acid; GABARA1A, the 𝛼1 subunit of the GABA_A_ receptor; GAPDH, glyceraldehyde-3-dehydrogenase; GAS5, long non-coding RNA growth arresting-specifc 5; GC, gas chromatography; GC-MS, gas chromatography–mass spectrometry; GCLC, glutamate-cysteine ligase catalytic subunit; GCLM, glutamate-cysteine ligase modifier subunit; GDH, Glutamate dehydrogenase; GFAP, glial fibrillary acid protein; Glo-1, glyoxalase 1; GLR, glutathione reductase; Glu, glutamate; GPx, glutathione peroxidase; GR, glucocorticoid receptor; GSH, glutathione; GSK-3β, glycogen synthase kinase 3-beta; GST, Glutathione S transferase; GSTP1, Glutathione S-transferase Pi; GSTω1, Glutathione S-transferase omega-1; GSSG, glutathione disulphide; G6PD, glucose-6-phosphodehydrogenase; h, hours; Ham-D, Hamilton Depression Rating Scale; HAPI, microglial cell line Highly Aggressively Proliferating Immortalised; HCAR2, Hydroxycarboxylic Acid Receptor 2; HC, healthy control(s); HDAC2, Histone Deacetylase 2 gene; HDOs, heteroduplex oligonucleotides; HFD, high fat diet; Hippoc, hippocampus, hippocampal; HippP, hippocampus proper; HO-1, haemoxygenase; HPLC, high-performance liquid chromatography; HRR, High resolution respirometry; Hsd, hesperidin; hsp-60, -70, 60 and 70 kDa heat shock proteins; Hst, hesperetin, i.e., the aglycone of the flavonoid hesperidin; H&E, Haematoxylin-Eosin; H_2_DCF-DA, 2′,7′ -dichlorodihydrofluorescein diacetate; H_2_O, water; H3K27me3, Methylation of histone 3 on lysine 27; IBA-1, ionised calcium-binding adapter molecular 1; IBF, inflammatory bowel disease; ICP-MS, inductively coupled plasma mass spectrometry; ICR, imprinting control region (mice); icv, intracerebroventricular; IDO-1, indoleamine-2,3-dioxygenase 1; IF, immunofluorescence; i.g., intragastric gavage; IKK-β, inhibitor of nuclear factor kappa-B kinase subunit beta; IL-1…10, Interleukin 1, 2, 3, …-10; IL-1β, Interleukin 1-beta; inflam, inflammation, inflammatory; ImBl, immunoblotting; IMI, Imipramine Hydrocloride; iNOS, inducible nitric oxide synthase; ip, intraperitoneal; Ipt, iptakalim IκBα, nuclear factor of kappa light polypeptide gene enhancer in B-cells inhibitor, alpha; i-gastr, intragastrically; JNK, c-Jun N-terminal kinase; jugl., juglone, a naphthoquinone from walnut; Keap1, kelch-like ECH-associated protein 1; KMO, kynurenine monooxygenase; KO, knock-out; KT, ketamine; L-dopa, levodopa, L-3,4-dihydroxyphenylalanine; LDB, light-dark box; lenti, lentivirus; Lenti-GAS5, lentivirus-packaged GAS5 overexpressing plasmid; LPO, lipid peroxidase; LPS, lipopolysaccharide; LW, long day and warm temperature; M1, Macrophage M1 phenotype, MAO, Monoamine oxidase; MAPK, mitogen-activated protein kinase; MBT, marble burying test; MCE-1, (±)-3-ethynyl-3-methyl-6-oxocyclohexa-1,4-dienecarbonitrile, Nrf2 activator; MCC, MCC950; MCh, mitochondrium, mitochondrial; MDA, malondialdehyde; MDD, major depressive disorder; Measur, measurement, measured; *MeCP2*, methyl CpG binding protein 2; MEK1/2, mitogen-activated protein kinase kinases 1 and 2; Mito–TEMPO, 2-(2,2,6,6-Tetramethylpiperidin-1-oxyl-4-ylamino)-2-oxoethyl) triphenylphosphonium chloride; MMF, Monomethyl fumarate; MnSOD, manganese superoxide dismutase; MT, melatonin; NA, noradrenaline; NAC, *N*-acetyl-Lcysteine; NADP, Nicotinamide adenine dinucleotide phosphate; NBP, Dl-3-n-Butylphthalide; NBT, nesting building test; NDEVs, Enrichment of plasma neuron-derived extracellular vesicles; NF-κB, nuclear factor kappa B; NI, neuroinflammation; NKT, Nootkatone; NLRP3, Nucleotide-binding oligomerization domain containing 3 inflammasome; NO, nitric oxide; NORT, Novel Object Recognition Test; NOs, nitric oxide synthase; NOX, NADP oxidase; NQO-1, NAD(P)H Quinone Dehydrogenase (oxidoreductase) 1; NResp, non-responder(s); Nrf2, redox-sensing nuclear factor (erythroid-derived 2)-like 2; NS, non-silencing; NSFT, Novelty-suppressed feeding test; NTAE, Nicotiana tabacum; OBX, olfactory bulbectomy; OFT, open-field test; OS, oxidative stress; OVX, ovariectomised, ovariectomy; p, phospho, phosphorylated; *p,* statistical significance probability; PB, Pinocembrin; PBMC, peripheral blood mononuclear cells; PBS, phosphate-buffered saline; (p-ClPhSe)2, *p*-chlorodiphenyl diselenide; PCMS, predictable, chronic mild stress; PD, Polydatin, a resveratrol derivative of the herbal medicine *Polygonum cuspidatum*; PFC, prefrontal cortex, dl, dorsolateral, vm, ventromedial; PI3K, phosphatidylinositol 3-kinase; plac, placebo or vehicle or saline; p.o., *per os*, orally; PG, prostaglandin; PGC-1α, peroxisome proliferator-activated receptor-gamma coactivator; PPAR-α, -γ, Peroxisome Proliferator Receptor-alpha, -gamma; PRC2, Polycomb repressive complex 2; PRMT1, protein arginine methyltransferase; PSD-95, 95 kD post-synaptic density protein; pts, patients; PSE, pinus spp. succinum extract;. PSP, Polysaccharides from *Polygonatum cyrtonema*; PTZ, pentylenetetrazol; QA quinolinic acid; qPCR, quantitative polymerase chain reaction; RA, rosmarinic acid; Rb1, Retinoblastoma protein-1; Resp, responder(s); Rg1, Ginsenoside Rg1, the main active compound of ginseng; RI, recombinant inbred; RIP, RNA-binding protein immunoprecipitation; ROS, reactive oxygen species; Ro61-8048, 3,4-dimethoxy-N-[4-(3-nitrophenyl)thiazol-2-yl]benzenesulfonamide, high-affinity kynurenine 3-hydroxylase (KMO) inhibitor; rt-qPCR, real-time quantitative polymerase chain reaction; SAHA, suberoyanilide hydroxamic acid, vorinostat, a histone deacetylase inhibitor; SalB, Salvianolic Acid; SC, short day and cool temperature; SDS, social defeat stress; *SE*, *Salicornia europaea*; SEO, *Schisandra Chinensis* oil; SF spectrofluorometer; SFN, sulforaphane; shGAS5, lentivirus-packaged GAS5 shRNA; shNC, shRNA-containing lentivirus, negative-control; shRNA, short (small) hairpin RNA (to silence specific genes, e.g., lentivirus-packaged GAS5 shRNA); SI, social isolation; siRNA, small interfering ribonucleic acid; SIRT1, sirtuin-1, silent information regulator 2 homolog 1; SIT, social interaction test; SNAP25, 25kD synaptosomal associated protein; SnPP-IX, tin protoporphyrin IX; SOCS3, Suppressor of cytokine signalling 3; SODs, superoxide dismutases; SPlT, splash test; SPT, sucrose preference test; SS, social support; STAT6, signal transducer and activator of transcription 6; STZ, streptozotocin; SUS, CUMS-susceptible; TBAR, Thiobarbituric acid reactive substance; TBE-31, (±)-(4bS,8aR,10aS)-10a-ethynyl-4b,8,8-trimethyl-3,7-dioxo-3,4b,7,8,8a,9,10,10a-octahydrophenanthrene-2,6-dicarbonitrile, Nrf2 activator; *t*-BHQ, *tert*-Butylhydroquinone, Nrf2 nuclear translocator; TEM, transmission electron microscopy; Tempol, 4-hydroxy-2,2,6,6-tetramethylpiperidine 1-oxyl; 288 μmol/kg-1‧day-1, an antioxidant; TLR4, Toll-like receptor 4; TMS, transcranial magnetic stimulation, d, deep, r, repetitive; TNF-α, tumour necrosis factor-alpha; TREM2, Triggering receptor expressed on myeloid cell-2; TrkB, tropomyosin receptor kinase B; Trx, thioredoxin; TrxR, thioredoxin reductase; TSP, Tilapia Skin Peptides; TST, tail suspension test; TUNEL, Terminal-deoxynucleoitidyl Transferase mediated Nick end labelling; TXNIP, thioredoxin-interacting protein; UA, ursolic acid; UnA, unfiltered air; WB, Western blot(ting); Wfs1, Wolfram syndrome 1; wk, week(s); WT, wild type; $\bar{x}$, mean; xCT, solute carrier family 7 member 11, SLC7A11; YFP, Thy1-yellow fluorescent protein; Xn, Xanthohumol; yr(s), year(s); ZnPP, zinc protoporphyrin IX; ZT, Zileuton; γ-GCS, γ-glutamylcysteine synthetase; 4-HNE, 4-hydroxy-2-nonenal; 5-HIAA, 5-hydroxyindoleacetic acid; 5-HT, 5-hydroxytryptamine, serotonin; 8-OHdG, 8-hydroxy-2′-deoxyguanosine; ±, SD, standard deviation; ×, for, per; ≈, about equal, not different; ♁, females; ♂, males; ↓, decreased, lower; ↑, increased, higher, →, induced, followed.

## 4. Discussion

In this review, we collected data on animal and human studies on depression and the role of Nrf2 as a probe of the antioxidant system function and found an antidepressant effect to be the consequence of the activation of the Nrf2-HO-1 pathway, with no studies pointing to the opposite direction. In this context, the role of neuroinflammation in being associated with depressive-like states clearly emerges, as well as the role of antioxidant systems in countering neuroinflammation and its markers, with Nrf2 standing at its crossroad. We may thus suppose that depression involves multiple systems and the antioxidant system, and Nrf2, in particular, counteract many actions of the failing multi-system state associated with depressive behaviour. Many of the substances that help overcome the alterations caused in the organism by depression are of natural origin and constitute long-used remedies for many other conditions. The antioxidant research line in depression that focuses on the actions of Nrf2 is relatively recent, dating back to 2013 [43], although the substance was isolated in 1994 [126], almost three decades ago.

The rate of publications on this issue has considerably increased across the years, with papers focusing on psychiatric disorders increasing almost exponentially from 26 in 2020 to 37 in 2021 and to 58 in 2022, witnessing increased interest and awareness of its importance in the pathophysiology of psychiatric disorders. Studies on depression reflect a similar trend. From eight studies in 2019, which increased to 16 in 2020, 18 in 2021, and 23 in 2022 to the beginning of 2023.

Most of the studies were conducted on rodents, mainly mice and, most importantly, male animals. This limits the extension of results to female animals, but it should be noted that findings in females were not dissimilar from those in males.

The studies included here were based on different animal depression paradigms, with many studies using stress-induced DLB, mostly elicited by LPS [40,43,51,53,58,59,63,65,67,73,75,79,85,88,95,105,106,125], or through corticosterone or cortisol [44,46,80,87,98], while other studies used chronic unpredictable mild stress [61,72,76,83,93,96,97,99,100,101,106,108,111,122,124] or social defeat stress [50,52,88,102,107,120]. Two studies used tumour-related depression models [71,91], while just one study each used post-ovariectomised depression [121], pentylenetetrazol-induced seizures [116], maternal separation [117], and olfactory bulbectomy [113] models (Table 1). Various studies used Nrf2 knock-out animals [49,62,84,88,120] or knock-out of the inflammation and histone methylation modulator protein arginine methyltransferase [67]. Nrf2 knock-out or knock-down apparently impairs neurochemical indexes and behavioural test performance, while protein arginine methyltransferase knock-out decreases depressive indexes [67]. Despite heterogeneity, findings all pointed to the Nrf2-antioxidant pathway restoring impaired neurochemistry and behavioural test performance.

Anti-inflammatory or anti-neuroinflammatory effects of employed antidepressants and putative antidepressant substances, such as the many plant extracts used in the studies we considered here, were shown to be related to various improvements in neurochemical indexes and behavioural tests [43,48,59,71,76,81,85,91,95,108,111,123,125].

**Figure 2:**
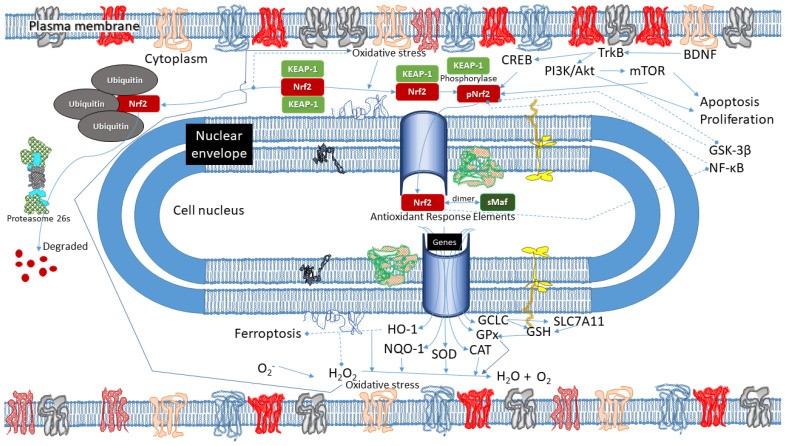
Simplified depiction of the mechanism of action of Nrf2 and the pathways it interacts with. Arrows, activation; broken lines with rhomboid ending, inhibition. *Abbreviations*. Akt, Protein kinase B; BDNF, brain-derived neurotrophic factor; CREB, cyclic AMP responsive element binding protein; GCLC, Glutamate-cysteine ligase; GPx, glutathione peroxidase; GSH, glutathione; GSK-3β, glycogen synthase kinase 3-beta; HO-1, haem oxygenase 1; mTOR, mammalian target of rapamycin; NF-κB, nuclear factor kappa B; NQO-1, NAD(P)H quinone dehydrogenase 1; PI3K, phosphatidylinositol-3-kinase; SLC7A11, Solute Carrier Family 7 Member 11 (cystine/glutamate transporter); sMAF, small musculoaponeurotic fibrosarcoma proteins; SOD, superoxide dismutase; TrkB, tropomyosin receptor kinase B.

Studies were consistent in measuring cyclic AMP responsive element binding protein (CREB) activity, in that increased phospho-CREB or CREB levels were associated with an antidepressant response [38,46,81,83,103,109,112]; remarkably, studies on peripheral tissues in man [38] and hippocampal content in mice [81] pointed to the same direction. Additionally, studies of BDNF showed that increased BDNF activity was associated with antidepressant effects and that animals displaying depressive features have low BDNF contents in the periphery and the brain [44,52,57,57,58,66,67,69,81,83,85,88,93,111,112,114,121]. It has recently been shown that most, if not all, antidepressant drugs bring about their antidepressant effects by binding the tyrosine kinase receptor 2 (TrkB), a molecule tied to the action of BDNF [127,128]. The findings of the studies reviewed here quite match this concept; increased TrkB/BDNF signalling was related to antidepressant effects, and low levels were associated with depressive behaviour [11,50,66,69,84,93,103,111,120]. Increased activity also of protein kinase B (Akt-1), which is an antioxidant in the wingless-GSK pathway and carries on some of the biological [128,129,130] and clinical [131] actions of lithium, which also inhibits GSK-3β [132,133,134], was found to be associated with antidepressant effects in the studies included in this review [42,45,47,74,75,93,94,110,119]. On the opposite side are found nuclear factor kappa B (NF-κB) and glycogen synthase kinase (GSK) pathways; the higher the NF-κB [38,39,67,69,73,75,80,85,91,98,100,103,113,115] and GSK3β levels [45,79,109], the worse the depressive indexes. Therefore, it appears that on one side, there are antioxidant systems and growth factors promoting plasticity, such as Nrf2–HO-1, BDNF, Akt, TrkB, and CREB, which are protective from depression and, on the other side, GSK-3β and NF-κB, which promote depressive behaviour (Figure 2). Therefore, we may suppose that new antidepressant drug discovery could involve promoting drugs that increase Nrf2–HO-1, BDNF, Akt, TrkB, and CREB activity, while downplaying GSK-3β and NF-κB. However, it is not so simple to identify the characteristics of drugs that act only where they will wishfully carry on the desired effect because drugs acting within cells must cross plasma membranes selectively, reach their intracellular target in those cells needing their actions, and not everywhere. Depression is a multi-system and multi-organ condition but not an all-system derangement, so the presence of an extraordinarily penetrating drug in some cells could be related to undesirable or adverse effects. Furthermore, not all actions of Nrf2 are good for the body. Nrf2 may promote tumours [135,136] and atherogenesis [137,138], so any drug acting on Nrf2 must avoid these two and maybe other unknown potentially harmful actions.

## 5. Limitations

This review has several limitations in that the animal studies focused mostly on rodents (mice and rats), and few focused on other animals (fish and nematodes) or cell lines; therefore, the results cannot extend to other animal species. Human studies were only four (one *post mortem*), and their methodologies were too different from animal protocols; hence the results from the latter are not translatable to the former. Furthermore, a sex bias exists in animal studies (mostly males) and not in the few human studies. Depression is overrepresented in women in humans, so it might be that testing it in male animals is not a good idea, but female rodents pose significant problems in conducting scientific experiments using the paradigms of the studies included in this review. The animal models of depression are not easy to translate to humans and into clinical practice.

## 6. Conclusions

Nrf2 is at the crossroad of many cellular actions. It directs antioxidant pathways and receives many intracellular signals to which it attempts to respond in a balanced way. Its function appears to be impaired in depression, and it is also possible that its manipulation could prove to be beneficial in human depression; however, there is much to discover. There is hope to discover drugs that cross the blood–brain barrier, or use some phytopharmaca or their derivatives, such as gastrodin, or use drugs stimulating anti-inflammatory cytokines that could counteract neuroinflammation. From the studies included in this review, depression appears to be strictly tied to inflammatory mechanisms. Provided that new drugs acting on the Nrf2 antioxidant pathway can avoid tumorigenesis and atherogenesis, Nrf2 can be a useful target for novel drug development.

## Data Availability

The data presented in this study are available in the article and Supplementary Materials.

## Supplementary Materials

The following supporting information can be downloaded at: https://www.mdpi.com/article/10.3390/antiox12040817/s1, Online Supplement.
