# Supplementary material for: The Nrf2 Pathway in Depressive Disorders: A Systematic Review of Animal and Human Studies
Source: Antioxidants (Basel). 2023 Mar 27;12(4):817. doi: 10.3390/antiox12040817 (PMC10135298; doi:10.3390/antiox12040817)
Supplement: Supplementary file 1 [file antioxidants-12-00817-s001.zip › antioxidants-2248926-supplementary.pdf]

Section and Topic	Item #	Checklist item	Location where item is reported
**TITLE**			
Title	1	Identify the report as a systematic review.	1
**ABSTRACT**			
Abstract	2	See the PRISMA 2020 for Abstracts checklist.	2
**INTRODUCTION**			
Rationale	3	Describe the rationale for the review in the context of existing knowledge.	3-4
Objectives	4	Provide an explicit statement of the objective(s) or question(s) the review addresses.	4
**METHODS**			
Eligibility criteria	5	Specify the inclusion and exclusion criteria for the review and how studies were grouped for the syntheses.	4
Information sources	6	Specify all databases, registers, websites, organisations, reference lists and other sources searched or consulted to identify studies. Specify the date when each source was last searched or consulted.	4
Search strategy	7	Present the full search strategies for all databases, registers and websites, including any filters and limits used.	4
Selection process	8	Specify the methods used to decide whether a study met the inclusion criteria of the review, including how many reviewers screened each record and each report retrieved, whether they worked independently, and if applicable, details of automation tools used in the process.	4
Data collection process	9	Specify the methods used to collect data from reports, including how many reviewers collected data from each report, whether they worked independently, any processes for obtaining or confirming data from study investigators, and if applicable, details of automation tools used in the process.	4
Data items	10a	List and define all outcomes for which data were sought. Specify whether all results that were compatible with each outcome domain in each study were sought (e.g. for all measures, time points, analyses), and if not, the methods used to decide which results to collect.	4
	10b	List and define all other variables for which data were sought (e.g. participant and intervention characteristics, funding sources). Describe any assumptions made about any missing or unclear information.	4
Study risk of bias assessment	11	Specify the methods used to assess risk of bias in the included studies, including details of the tool(s) used, how many reviewers assessed each study and whether they worked independently, and if applicable, details of automation tools used in the process.	4
Effect measures	12	Specify for each outcome the effect measure(s) (e.g. risk ratio, mean difference) used in the synthesis or presentation of results.	4
Synthesis methods	13a	Describe the processes used to decide which studies were eligible for each synthesis (e.g. tabulating the study intervention characteristics and comparing against the planned groups for each synthesis (item #5)).	N/A
	13b	Describe any methods required to prepare the data for presentation or synthesis, such as handling of missing summary statistics, or data conversions.	N/A
	13c	Describe any methods used to tabulate or visually display results of individual studies and syntheses.	4,6,7-33
	13d	Describe any methods used to synthesize results and provide a rationale for the choice(s). If meta-analysis was performed, describe the model(s), method(s) to identify the presence and extent of statistical heterogeneity, and software package(s) used.	N/A
	13e	Describe any methods used to explore possible causes of heterogeneity among study results (e.g. subgroup analysis, meta-regression).	N/A
	13f	Describe any sensitivity analyses conducted to assess robustness of the synthesized results.	N/A
Reporting bias	14	Describe any methods used to assess risk of bias due to missing results in a synthesis (arising from reporting biases).	4, Suppl

Section and Topic	Item #	Checklist item	Location where item is reported
assessment			
Certainty assessment	15	Describe any methods used to assess certainty (or confidence) in the body of evidence for an outcome.	N/A
**RESULTS**			
Study selection	16a	Describe the results of the search and selection process, from the number of records identified in the search to the number of studies included in the review, ideally using a flow diagram.	4-33
	16b	Cite studies that might appear to meet the inclusion criteria, but which were excluded, and explain why they were excluded.	6, Suppl.
Study characteristics	17	Cite each included study and present its characteristics.	7-33
Risk of bias in studies	18	Present assessments of risk of bias for each included study.	Suppl
Results of individual studies	19	For all outcomes, present, for each study: (a) summary statistics for each group (where appropriate) and (b) an effect estimate and its precision (e.g. confidence/credible interval), ideally using structured tables or plots.	N/A
Results of syntheses	20a	For each synthesis, briefly summarise the characteristics and risk of bias among contributing studies.	Suppl
	20b	Present results of all statistical syntheses conducted. If meta-analysis was done, present for each the summary estimate and its precision (e.g. confidence/credible interval) and measures of statistical heterogeneity. If comparing groups, describe the direction of the effect.	4-5, N/A
	20c	Present results of all investigations of possible causes of heterogeneity among study results.	4,34
	20d	Present results of all sensitivity analyses conducted to assess the robustness of the synthesized results.	4,34
Reporting biases	21	Present assessments of risk of bias due to missing results (arising from reporting biases) for each synthesis assessed.	N/A
Certainty of evidence	22	Present assessments of certainty (or confidence) in the body of evidence for each outcome assessed.	N/A
**DISCUSSION**			
Discussion	23a	Provide a general interpretation of the results in the context of other evidence.	34
	23b	Discuss any limitations of the evidence included in the review.	34
	23c	Discuss any limitations of the review processes used.	34-35
	23d	Discuss implications of the results for practice, policy, and future research.	34-35
**OTHER INFORMATION**			
Registration and protocol	24a	Provide registration information for the review, including register name and registration number, or state that the review was not registered.	N/A
	24b	Indicate where the review protocol can be accessed, or state that a protocol was not prepared.	N/A
	24c	Describe and explain any amendments to information provided at registration or in the protocol.	N/A
Support	25	Describe sources of financial or non-financial support for the review, and the role of the funders or sponsors in the review.	35
Competing interests	26	Declare any competing interests of review authors.	35

Section and Topic	Item #	Checklist item	Location where item is reported
Availability of data, code and other materials	27	Report which of the following are publicly available and where they can be found: template data collection forms; data extracted from included studies; data used for all analyses; analytic code; any other materials used in the review.	N/A

From: Page MJ, McKenzie JE, Bossuyt PM, Boutron I, Hoffmann TC, Mulrow CD, et al. The PRISMA 2020 statement: an updated guideline for reporting systematic reviews. BMJ 2021;372:n71. doi: 10.1136/bmj.n71

For more information, visit: http://www.prisma-statement.org/

1	Li YJ, He XL, Zhang JY, Liu XJ, Liang JL, Zhou Q, Zhou GH. 8-O-acetyl shanzhiside methylester protects against sleep deprivation-induced cognitive deficits and anxiety-like behaviors by regulating NLRP3 and Nrf2 pathways in mice. Metab Brain Dis. 2022 Dec 2. doi: 10.1007/s11011-022-01132-z. Epub ahead of print. PMID: 36456714.	Insomnia
2	Yang G, Yang L, Liu Q, Zhu Z, Yang Q, Liu J, Beta T. Protective effects of cyanidin-3-*O*-glucoside on BPA-induced neurodevelopmental toxicity in zebrafish embryo model. Comp Biochem Physiol C Toxicol Pharmacol. 2022 Nov 19;264:109525. doi: 10.1016/j.cbpc.2022.109525. Epub ahead of print. PMID: 36410639.	*Off-target*
3	Natural Dietary Supplement, Carvacrol, Alleviates LPS-Induced Oxidative Stress, Neurodegeneration, and Depressive-Like Behaviors via the Nrf2/HO-1 Pathway [Retraction]. J Inflamm Res. 2022 Nov 7;15:6071-6072. doi: 10.2147/JIR.S395890. PMID: 36386579; PMCID: PMC9651034. Naeem K, Tariq Al Kury L, Nasar F, et al. J Inflamm Res. 2021;14:1313–1329.	*Duplicate= 79*
4	Ran Q, Zhang C, Wan W, Ye T, Zou Y, Liu Z, Yu Y, Zhang J, Shen B, Yang B. Pinocembrin ameliorates atrial fibrillation susceptibility in rats with anxiety disorder induced by empty bottle stimulation. Front Pharmacol. 2022 Oct 20;13:1004888. doi: 10.3389/fphar.2022.1004888. PMID: 36339600; PMCID: PMC9631028.	Anxiety
5	Zuo C, Cao H, Song Y, Gu Z, Huang Y, Yang Y, Miao J, Zhu L, Chen J, Jiang Y, Wang F. Nrf2: An all-rounder in depression. Redox Biol. 2022 Oct 31;58:102522. doi: 10.1016/j.redox.2022.102522. Epub ahead of print. PMID: 36335763; PMCID: PMC9641011.	Review
6	**Samy DM, Mostafa DK, Saleh SR, Hassaan PS, Zeitoun TM, Ammar GAG, Elsokkary NH. Carnosic Acid Mitigates Depression-Like Behavior in Ovariectomized Mice via Activation of Nrf2/HO-1 Pathway. Mol Neurobiol. 2022 Nov 4. doi: 10.1007/s12035-022-03093-x. Epub ahead of print. PMID: 36331794.**	**Depression**
7	Kholghi A, Hatami H, Khajehnasiri N, Sadeghian R. Intraperitoneal injection of buprenorphine on anxiety-like behavior and alteration in expression of *Gfap* and *Nrf2* in methamphetamine treated rats. Vet Res Forum. 2022;13(3):417-422. doi: 10.30466/vrf.2021.140211.3111. Epub 2022 Sep 15. PMID: 36320298; PMCID: PMC9548217.	Anxiety
8	**He L, Zheng Y, Huang L, Ye J, Ye Y, Luo H, Chen X, Yao W, Chen J, Zhang JC. Nrf2 regulates the arginase 1^{+} microglia phenotype through the initiation of TREM2 transcription, ameliorating depression-like behavior in mice. Transl Psychiatry. 2022 Oct 31;12(1):459. doi: 10.1038/s41398-022-02227-y. PMID: 36316319; PMCID: PMC9622811.**	**Depression**
9	Abu-Elfotuh K, Abdel-Sattar SA, Abbas AN, Mahran YF, Alshanwani AR, Hamdan AME, Atwa AM, Reda E, Ahmed YM, Zaghlool SS, El-Din MN. The protective effect of thymoquinone or/and thymol against monosodium glutamate-induced attention- deficit/hyperactivity disorder (ADHD)-like behavior in rats: Modulation of Nrf2/HO-1, TLR4/NF-κB/NLRP3/caspase-1 and Wnt/β-Catenin signaling pathways in rat model. Biomed Pharmacother. 2022;155:113799. doi: 10.1016/j.biopha.2022.113799. Epub 2022 Oct 6. PMID: 36271575.	ADHD
10	Liu Y, Azad MAK, Kong X, Zhu Q, Yu Z. Dietary bile acids supplementation modulates immune response, antioxidant capacity, glucose, and lipid metabolism in normal and intrauterine growth retardation piglets. Front Nutr. 2022 Sep 21;9:991812. doi: 10.3389/fnut.2022.991812. PMID: 36211492; PMCID: PMC9534482.	*Off-target*
11	**Sun JY, Liu YT, Jiang SN, Guo PM, Wu XY, Yu J. Essential oil from the roots of *Paeonia lactiflora pall.* has protective effect against corticosterone-induced depression in mice via modulation of PI3K/Akt signaling pathway. Front Pharmacol. 2022;13:999712. doi: 10.3389/fphar.2022.999712. PMID: 36188568; PMCID: PMC9523509.**	**Depression**
12	**Pei, H.; Zeng, J.; He, Z.; Zong, Y.; Zhao, Y.; Li, J.; Chen, W.; Du, R. Palmatine ameliorates LPS-induced HT-22 cells and mouse models of depression by regulating apoptosis and oxidative stress. J. Biochem. Mol. Toxicol., 2022 Sep 28, e23225. doi: 10.1002/jbt.23225. Epub ahead of print. PMID: 36169195.**	**Depression**
13	**Nasehi, L.; Morassaei, B.; Ghaffari, M.; Sharafi, A.; Dehpour, A.R.; Hosseini, M.J. The impacts of vorinostat on NADPH oxidase and mitochondrial biogenesis gene expression in the heart of mice model of depression. Can. J. Physiol. Pharmacol., 2022 Sep 27. doi: 10.1139/cjpp-2022-0098. Epub ahead of print. PMID: 36166834.**	**Depression**

14	**Wang X, Xiao A, Yang Y, Zhao Y, Wang CC, Wang Y, Han J, Wang Z, Wen M. DHA and EPA prevent seizure and depression-like behavior by inhibiting ferroptosis and neuroinflammation via different mode-of-actions in a pentylenetetrazole-induced kindling model in mice. Mol Nutr Food Res. 2022 Nov;66(22):e2200275. doi: 10.1002/mnfr.202200275. Epub 2022 Sep 26. PMID: 36099650.**	**Depression**
15	Shi J, Hou J, Sun Y, Jia Z, Zhou Y, Wang C, Zhao H. Chaihujialonggumulitang shows psycho-cardiology therapeutic effect on acute myocardial infarction with comorbid anxiety by the activation of Nrf2/HO-1 pathway and suppression of oxidative stress and apoptosis. Biomed Pharmacother. 2022;153:113437. doi: 10.1016/j.biopha.2022.113437. Epub 2022 Jul 21. PMID: 36076489.	Anxiety
16	**Kang JY, Baek DC, Son CG, Lee JS. Succinum extracts inhibit microglial- derived neuroinflammation and depressive-like behaviors. Front Pharmacol. 2022;13:991243. doi: 10.3389/fphar.2022.991243. PMID: 36052132; PMCID: PMC9425083.**	**Depression**
17	Salem HA, Elsherbiny N, Alzahrani S, Alshareef HM, Abd Elmageed ZY, Ajwah SM, Hamdan AME, Abdou YS, Galal OO, El Azazy MKA, Abu-Elfotuh K. Neuroprotective Effect of Morin Hydrate against Attention-Deficit/Hyperactivity Disorder (ADHD) Induced by MSG and/or Protein Malnutrition in Rat Pups: Effect on Oxidative/Monoamines/Inflammatory Balance and Apoptosis. Pharmaceuticals (Basel). 2022 Aug 17;15(8):1012. doi: 10.3390/ph15081012. PMID: 36015160; PMCID: PMC9415807.	ADHD
18	Sağlam C, Turan İ, Özaçmak HS. The effect of glucagon like peptide-1 receptor agonist on behavioral despair and anxiety-like behavior in ovariectomized rats: Modulation of BDNF/CREB, Nrf2 and lipocalin 2. Behav Brain Res. 2022 Oct 28;435:114053. doi: 10.1016/j.bbr.2022.114053. Epub 2022 Aug 9. PMID: 35961539.	Anxiety
19	Garcia-Partida JA, Torres-Sanchez S, MacDowell K, Fernández-Ponce MT, Casas L, Mantell C, Soto-Montenegro ML, Romero-Miguel D, Lamanna-Rama N, Leza JC, Desco M, Berrocoso E. The effects of mango leaf extract during adolescence and adulthood in a rat model of schizophrenia. Front Pharmacol. 2022;13:886514. doi: 10.3389/fphar.2022.886514. PMID: 35959428; PMCID: PMC9360613.	SZ
20	He MC, Feng R, Wang J, Xia SH, Wang YJ, Zhang Y. Prevention and treatment of natural products from Traditional Chinese Medicine in depression: Potential targets and mechanisms of action. Front Aging Neurosci. 2022 Jul 18;14:950143. doi: 10.3389/fnagi.2022.950143. PMID: 35923544; PMCID: PMC9339961.	Review
21	**Li X, Wang YB, Wang CC, Jing R, Mu LH, Liu P, Hu Y. Antidepressant mechanism of kaixinsan and its active compounds based on upregulation of antioxidant thioredoxin. Evid Based Complement Alternat Med. 2022;2022:7302442. doi: 10.1155/2022/7302442. PMID: 35911169; PMCID: PMC9325646.**	**Depression**
22	**Yu Y, Li Y, Qi K, Xu W, Wei Y. Rosmarinic acid relieves LPS-induced sickness and depressive-like behaviors in mice by activating the BDNF/Nrf2 signaling and autophagy pathway. Behav Brain Res. 2022;433:114006. doi: 10.1016/j.bbr.2022.114006. Epub 2022 Jul 16. PMID: 35843463.**	**Depression**
23	Fernandes BS, Dai Y, Jia P, Zhao Z. Charting the proteome landscape in major psychiatric disorders: From biomarkers to biological pathways towards drug discovery. Eur Neuropsychopharmacol. 2022;61:43-59. doi: 10.1016/j.euroneuro.2022.06.001. Epub 2022 Jun 25. PMID: 35763977; PMCID: PMC9378550.	Review
24	**Bansal Y, Singh R, Sodhi RK, Khare P, Dhingra R, Dhingra N, Bishnoi M, Kondepudi KK, Kuhad A. Kynurenine monooxygenase inhibition and associated reduced quinolinic acid reverses depression-like behaviour by upregulating Nrf2/ARE pathway in mouse model of depression: In-vivo and In-silico studies. Neuropharmacology. 2022;215:109169. doi: 10.1016/j.neuropharm.2022.109169. Epub 2022 Jun 23. PMID: 35753430.**	**Depression**
25	Zhou B, Zhang J, Liu H, Chen S, Wang T, Wang C. Zinc oxide nanoparticle improves the intestinal function of intrauterine growth retardation finishing pigs *via* regulating intestinal morphology, inflammation, antioxidant status and autophagy. Front Vet Sci. 2022;9:884945. doi: 10.3389/fvets.2022.884945. PMID: 35733639; PMCID: PMC9207390.	*Off-target*
26	**Zhao YT, Yin H, Hu C, Zeng J, Zhang S, Chen S, Zheng W, Li M, Jin L, Liu Y, Wu W, Liu S.Tilapia skin peptides ameliorate cyclophosphamide-induced anxiety- and depression-like behavior *via* improving oxidative stress, neuroinflammation, neuron apoptosis, and neurogenesis in mice. Front Nutr. 2022 Jun 2;9:882175. doi: 10.3389/fnut.2022.882175. PMID: 35719151; PMCID: PMC9201437.**	**Depression**, Anxiety
27	Zhang Y, Zhang X, Yan Q, Xu C, Liu Q, Shen Y, Xu J, Wang G, Zhao P. Melatonin attenuates polystyrene microplastics induced motor neurodevelopmental defect in zebrafish (Danio rerio) by activating *nrf2 - isl2a* Axis. Ecotoxicol Environ Saf. 2022;241:113754. doi: 10.1016/j.ecoenv.2022.113754. Epub 2022 Jun 13. PMID: 35709674.	*Off-target*
28	Ershova ES, Shmarina GV, Martynov AV, Zakharova NV, Veiko RV, Umriukhin PE, Kostyuk GP, Kutsev SI, Veiko NN, Kostyuk SV. NADPH-oxidase 4 gene over- expression in peripheral blood lymphocytes of the schizophrenia patients. PLoS One. 2022;17(6):e0269130. doi: 10.1371/journal.pone.0269130. PMID: 35696356; PMCID: PMC9191697.	SZ
29	**Wu X, Liu C, Wang J, Guan Y, Song L, Chen R, Gong M. Catalpol exerts antidepressant-like effects by enhancing anti-oxidation and neurotrophy and inhibiting neuroinflammation via activation of HO-1. Neurochem Res. 2022;47(10):2975-2991. doi: 10.1007/s11064-022-03641-w. Epub 2022 Jun 6. PMID: 35668334.**	**Depression**
30	**Tan L, Yang Y, Peng J, Zhang Y, Wu B, He B, Jia Y, Yan T. *Schisandra chinensis* (Turcz.) Baill. essential oil exhibits antidepressant-like effects and against brain oxidative stress through Nrf2/HO-1 pathway activation. Metab Brain Dis. 2022;37(7):2261-2275. doi: 10.1007/s11011-022-01019-z. Epub 2022 Jun 6. PMID: 35666395; PMCID: PMC9168360.**	**Depression**
31	**Fasakin OW, Oboh G, Ademosun AO, Lawal AO. The modulatory effects of alkaloid extracts of *Cannabis sativa*, *Datura stramonium*, *Nicotiana tabacum* and male *Carica papaya* on neurotransmitter, neurotrophic and neuroinflammatory systems linked to anxiety and depression. Inflammopharmacology. 2022;30(6):2447-2476. doi: 10.1007/s10787-022-01006-x. Epub 2022 Jun 5. PMID: 35665872.**	**Depression**, Anxiety

32	**de Souza AG, Lopes IS, Filho AJMC, Cavalcante TMB, Oliveira JVS, de Carvalho MAJ, de Lima KA, Jucá PM, Mendonça SS, Mottin M, Andrade CH, de Sousa FCF, Macedo DS, de França Fonteles MM. Neuroprotective effects of dimethyl fumarate against depression-like behaviors *via* astrocytes and microglia modulation in mice: possible involvement of the HCAR2/Nrf2 signaling pathway. Naunyn Schmiedebergs Arch Pharmacol. 2022;395(9):1029-1045. doi: 10.1007/s00210-022-02247-x. Epub 2022 Jun 4. PMID: 35665831.**	**Depression**
33	**Jiang N, Zhang Y, Yao C, Huang H, Wang Q, Huang S, He Q, Liu X. Ginsenosides Rb1 attenuates chronic social defeat stress-induced depressive behavior *via* regulation of SIRT1-NLRP3/Nrf2 pathways. Front Nutr. 2022;9:868833. doi: 10.3389/fnut.2022.868833. PMID: 35634375; PMCID: PMC9133844.**	**Depression**
34	Kenkhuis B, van Eekeren M, Parfitt DA, Ariyurek Y, Banerjee P, Priller J, van der Weerd L, van Roon-Mom WMC. Iron accumulation induces oxidative stress, while depressing inflammatory polarization in human iPSC-derived microglia. Stem Cell Reports. 2022;17(6):1351-1365. doi: 10.1016/j.stemcr.2022.04.006. Epub 2022 May 5. PMID: 35523178; PMCID: PMC9213827.	*Off-target*
35	Subba R, Ahmad MH, Ghosh B, Mondal AC. Targeting NRF2 in Type 2 diabetes mellitus and depression: Efficacy of natural and synthetic compounds. Eur J Pharmacol. 2022;925:174993. doi: 10.1016/j.ejphar.2022.174993. Epub 2022 May 2. PMID: 35513015.	Review
36	**Shen F, Xie P, Li C, Bian Z, Wang X, Peng D, Zhu G. Polysaccharides from *Polygonatum cyrtonema* Hua reduce depression-like behavior in mice by inhibiting oxidative stress-calpain-1-NLRP3 signaling axis. Oxid Med Cell Longev. 2022;2022:2566917. doi: 10.1155/2022/2566917. PMID: 35498131; PMCID: PMC9045988.**	**Depression**
37	Sever IH, Ozkul B, Bozkurt MF, Erbas O. Therapeutic effect of finasteride through its antiandrogenic and antioxidant role in a propionic acid-induced autism model: Demonstrated by behavioral tests, histological findings and MR spectroscopy. Neurosci Lett. 2022;779:136622. doi: 10.1016/j.neulet.2022.136622. Epub 2022 Apr 7. PMID: 35398534.	Autism
38	Deng Y, Liu H, Huang Q, Tu L, Hu L, Zheng B, Sun H, Lu D, Guo C, Zhou L. Mechanism of longevity extension of *Caenorhabditis elegans* induced by *Schizophyllum commune* fermented supernatant with added Radix Puerariae. Front Nutr. 2022;9:847064. doi: 10.3389/fnut.2022.847064. PMID: 35360681; PMCID: PMC8963188.	*Off-target*
39	Morris G, Sominsky L, Walder KR, Berk M, Marx W, Carvalho AF, Bortolasci CC, Maes M, Puri BK. Inflammation and Nitro-oxidative Stress as Drivers of Endocannabinoid System Aberrations in Mood Disorders and Schizophrenia. Mol Neurobiol. 2022 Jun;59(6):3485-3503. doi: 10.1007/s12035-022-02800-y. Epub 2022 Mar 26. PMID: 35347586.	Review
40	Zhao YH, Fu HG, Cheng H, Zheng RJ, Wang G, Li S, Li EY, Li LG. Electroacupuncture at Zusanli ameliorates the autistic-like behaviors of rats through activating the Nrf2-mediated antioxidant responses. Gene. 2022 Jun 20;828:146440. doi: 10.1016/j.gene.2022.146440. Epub 2022 Mar 23. PMID: 35339642.	Autism
41	Oh S, Rho NK, Byun KA, Yang JY, Sun HJ, Jang M, Kang D, Son KH, Byun K. Combined treatment of monopolar and bipolar radiofrequency increases skin elasticity by decreasing the accumulation of advanced glycated end products in aged animal skin. Int J Mol Sci. 2022;23(6):2993. doi: 10.3390/ijms23062993. PMID: 35328415; PMCID: PMC8950306.	*Off-target*
42	Abbasalipour H, Hajizadeh Moghaddam A, Ranjbar M. Sumac and gallic acid- loaded nanophytosomes ameliorate hippocampal oxidative stress via regulation of Nrf2/Keap1 pathway in autistic rats. J Biochem Mol Toxicol. 2022;36(6):e23035. doi: 10.1002/jbt.23035. Epub 2022 Mar 21. PMID: 35307911.	Autism
43	**Muhammad AJ, Hao L, Al Kury LT, Rehman NU, Alvi AM, Badshah H, Ullah I, Shah FA, Li S. Carveol promotes Nrf2 contribution in depressive disorders through an anti-inflammatory mechanism. Oxid Med Cell Longev. 2022;2022:4509204. doi: 10.1155/2022/4509204. PMID: 35295720; PMCID: PMC8920705.**	**Depression**
44	**Li J, Gao W, Zhao Z, Li Y, Yang L, Wei W, Ren F, Li Y, Yu Y, Duan W, Li J, Dai B, Guo R. Ginsenoside Rg1 reduced microglial activation and mitochondrial dysfunction to alleviate depression-like behaviour via the GAS5/EZH2/SOCS3/NRF2 axis. Mol Neurobiol. 2022;59(5):2855-2873. doi: 10.1007/s12035-022-02740-7. Epub 2022 Mar 1. PMID: 35230663; PMCID: PMC9016007.**	**Depression**
45	Wu H, Luan Y, Wang H, Zhang P, Liu S, Wang P, Cao Y, Sun H, Wu L. Selenium inhibits ferroptosis and ameliorates autistic-like behaviors of BTBR mice by regulating the Nrf2/GPx4 pathway. Brain Res Bull. 2022;183:38-48. doi: 10.1016/j.brainresbull.2022.02.018. Epub 2022 Feb 25. PMID: 35227767.	Autism
46	Nadeem A, Ahmad SF, Al-Harbi NO, Al-Ayadhi LY, Alanazi MM, Alfardan AS, Attia SM, Algahtani M, Bakheet SA. Dysregulated Nrf2 signaling in response to di(2-ethylhexyl) phthalate in neutrophils of children with autism. Int Immunopharmacol. 2022;106:108619. doi: 10.1016/j.intimp.2022.108619. Epub 2022 Feb 16. PMID: 35183033.	Autism
47	**Xia B, Liu X, Li X, Wang Y, Wang D, Kou R, Zhang L, Shi R, Ye J, Bo X, Liu Q, Zhao B, Liu X. Sesamol ameliorates dextran sulfate sodium-induced depression- like and anxiety-like behaviors in colitis mice: the potential involvement of the gut-brain axis. Food Funct. 2022;13(5):2865-2883. doi: 10.1039/d1fo03888e. PMID: 35179534.**	**Depression**, Anxiety
48	**Dang R, Wang M, Li X, Wang H, Liu L, Wu Q, Zhao J, Ji P, Zhong L, Licinio J, Xie P. Edaravone ameliorates depressive and anxiety-like behaviors via Sirt1/Nrf2/HO-1/Gpx4 pathway. J Neuroinflammation. 2022;19(1):41. doi: 10.1186/s12974-022-02400-6. PMID: 35130906; PMCID: PMC8822843.**	**Depression**, Anxiety
49	Beeraka NM, Avila-Rodriguez MF, Aliev G. Recent reports on redox stress- induced mitochondrial DNA variations, neuroglial interactions, and NMDA receptor system in pathophysiology of schizophrenia. Mol Neurobiol. 2022;59(4):2472-2496. doi: 10.1007/s12035-021-02703-4. Epub 2022 Jan 27. PMID: 35083660.	Review
50	Qi L, Jiang J, Zhang J, Zhang L, Wang T. Effect of maternal curcumin supplementation on intestinal damage and the gut microbiota in male mice offspring with intra-uterine growth retardation. Eur J Nutr. 2022;61(4):1875-1892. doi: 10.1007/s00394-021-02783-x. Epub 2022 Jan 21. PMID: 35059786.	*Off-target*

51	**Cheng WJ, Li P, Huang WY, Huang Y, Chen WJ, Chen YP, Shen JL, Chen JK, Long NS, Meng XJ. Acupuncture Relieves Stress-Induced Depressive Behavior by Reducing Oxidative Stress and Neuroapoptosis in Rats. Front Behav Neurosci. 2022;15:783056. doi: 10.3389/fnbeh.2021.783056. PMID: 35058758; PMCID: PMC8763975.**	**Depression**
52	Ma N, Wei G, Zhang H, Dai H, Roy AC, Shi X, Chang G, Shen X. Cis-9, trans-11 CLA alleviates lipopolysaccharide-induced depression of fatty acid synthesis by inhibiting oxidative stress and autophagy in bovine mammary epithelial cells. Antioxidants (Basel). 2021;11(1):55. doi: 10.3390/antiox11010055. PMID: 35052560; PMCID: PMC8773093.	*Off-target*
53	Abu-Elfotuh K, Al-Najjar AH, Mohammed AA, Aboutaleb AS, Badawi GA. Fluoxetine ameliorates Alzheimer's disease progression and prevents the exacerbation of cardiovascular dysfunction of socially isolated depressed rats through activation of Nrf2/HO^{-1} and hindering TLR4/NLRP3 inflammasome signaling pathway. Int Immunopharmacol. 2022;104:108488. doi: 10.1016/j.intimp.2021.108488. Epub 2022 Jan 15. PMID: 35042170.	*Off-target*
54	**Sun D-c, Wang R-r, Xu H, Zhu X-h, Sun Y, Qiao S-h, Qiao W. A network pharmacology-based study on antidepressant effect of *Salicornia europaea* L. extract with experimental support in chronic unpredictable mild stress model mice. Chin J Integr Med. 2022;28(4):339-348. doi: 10.1007/s11655-022-2879-2. Epub 2022 Jan 13. PMID: 35023063.**	**Depression**
55	Palaniyappan L, Sabesan P, Li X, Luo Q. Schizophrenia increases variability of the central antioxidant system: A meta-analysis of variance from MRS studies of glutathione. Front Psychiatry. 2021;12:796466. doi: 10.3389/fpsyt.2021.796466. PMID: 34916980; PMCID: PMC8669304.	Review
56	Bovilla VR, Kuruburu MG, Bettada VG, Krishnamurthy J, Sukocheva OA, Thimmulappa RK, Shivananju NS, Balakrishna JP, Madhunapantula SV. Targeted inhibition of anti-inflammatory regulator Nrf2 results in breast cancer retardation *in vitro* and *in vivo*. Biomedicines. 2021;9(9):1119. doi: 10.3390/biomedicines9091119. PMID: 34572304; PMCID: PMC8471069.	*Off-target*
57	**Guan Y, Wang J, Wu X, Song L, Wang Y, Gong M, Li B. Quercetin reverses chronic unpredictable mild stress-induced depression-like behavior *in vivo* by involving nuclear factor-E2-related factor 2. Brain Res. 2021;1772:147661. doi: 10.1016/j.brainres.2021.147661. Epub 2021 Sep 13. PMID: 34529966.**	**Depression**
58	Schrier MS, Zhang Y, Trivedi MS, Deth RC. Decreased cortical Nrf2 gene expression in autism and its relationship to thiol and cobalamin status. Biochimie. 2022;192:1-12. doi: 10.1016/j.biochi.2021.09.006. Epub 2021 Sep 10. PMID: 34517051.	Autism
59	**Song L, Wu X, Wang J, Guan Y, Zhang Y, Gong M, Wang Y, Li B. Antidepressant effect of catalpol on corticosterone-induced depressive-like behavior involves the inhibition of HPA axis hyperactivity, central inflammation and oxidative damage probably via dual regulation of NF-κB and Nrf2. Brain Res Bull. 2021;177:81-91. doi: 10.1016/j.brainresbull.2021.09.002. Epub 2021 Sep 6. PMID: 34500039.**	**Depression**
60	**Huang X, Qu Q, Ren D. Nrf2 alleviates cognitive dysfunction and brain inflammatory injury via mediating Wfs1 in rats with depression-like behaviors. Inflammation. 2022;45(1):399-413. doi: 10.1007/s10753-021-01554-4. Epub 2021 Sep 8. PMID: 34495404.**	**Depression**
61	Erten F. Lycopene ameliorates propionic acid-induced autism spectrum disorders by inhibiting inflammation and oxidative stress in rats. J Food Biochem. 2021;45(10):e13922. doi: 10.1111/jfbc.13922. Epub 2021 Sep 2. PMID: 34476820.	Autism
62	**Goetzl EJ, Wolkowitz OM, Srihari VH, Reus VI, Goetzl L, Kapogiannis D, Heninger GR, Mellon SH. Abnormal levels of mitochondrial proteins in plasma neuronal extracellular vesicles in major depressive disorder. Mol Psychiatry. 2021;26(12):7355-7362. doi: 10.1038/s41380-021-01268-x. Epub 2021 Sep 1. PMID: 34471251; PMCID: PMC8872999.**	**Depression**
63	**Tao W, Hu Y, Chen Z, Dai Y, Hu Y, Qi M. Magnolol attenuates depressive-like behaviors by polarizing microglia towards the M2 phenotype through the regulation of Nrf2/HO-1/NLRP3 signaling pathway. Phytomedicine. 2021;91:153692. doi: 10.1016/j.phymed.2021.153692. Epub 2021 Jul 30. PMID: 34411834.**	**Depression**
64	**Rahman SU, Ali T, Hao Q, He K, Li W, Ullah N, Zhang Z, Jiang Y, Li S. Xanthohumol attenuates lipopolysaccharide-induced depressive like behavior in mice: Involvement of NF-κB/Nrf2 signaling pathways. Neurochem Res. 2021;46(12):3135-3148. doi: 10.1007/s11064-021-03396-w. Epub 2021 Aug 16. PMID: 34398408.**	**Depression**
65	**Wu X, Wang J, Song L, Guan Y, Cao C, Cui Y, Zhang Y, Liu C. Catalpol Weakens Depressive-like Behavior in Mice with Streptozotocin-induced Hyperglycemia via PI3K/AKT/Nrf2/HO-1 Signaling Pathway. Neuroscience. 2021;473:102-118. doi: 10.1016/j.neuroscience.2021.07.029. Epub 2021 Aug 3. PMID: 34358633.**	**Depression**
66	**Wang J, Chen R, Liu C, Wu X, Zhang Y. Antidepressant mechanism of catalpol: Involvement of the PI3K/Akt/Nrf2/HO-1 signaling pathway in rat hippocampus. Eur J Pharmacol. 2021;909:174396. doi: 10.1016/j.ejphar.2021.174396. Epub 2021 Jul 29. PMID: 34332921.**	**Depression**
67	Tendilla-Beltrán H, Coatl-Cuaya H, Meneses-Prado S, Vázquez-Roque RA, Brambila E, Tapia-Rodríguez M, Martín-Hernández D, Garcés-Ramírez L, Madrigal JLM, Leza JC, Flores G. Neuroplasticity and inflammatory alterations in the nucleus accumbens are corrected after risperidone treatment in a schizophrenia- related developmental model in rats. Schizophr Res. 2021;235:17-28. doi: 10.1016/j.schres.2021.07.014. Epub 2021 Jul 20. PMID: 34298239.	SZ
68	Wang X, Liu J, Dai Z, Sui Y. Andrographolide improves PCP-induced schizophrenia-like behaviors through blocking interaction between NRF2 and KEAP1. J Pharmacol Sci. 2021;147(1):9-17. doi: 10.1016/j.jphs.2021.05.007. Epub 2021 May 24. PMID: 34294378.	SZ
69	**Zhu X, Zhang YM, Zhang MY, Chen YJ, Liu YW. Hesperetin ameliorates diabetes- associated anxiety and depression-like behaviors in rats via activating Nrf2/ARE pathway. Metab Brain Dis. 2021;36(7):1969-1983. doi: 10.1007/s11011-021-00785-6. Epub 2021 Jul 17. PMID: 34273043.**	**Depression**, Anxiety

70	Ren H, Han R, Liu X, Wang L, Koehler RC, Wang J. Nrf2-BDNF-TrkB pathway contributes to cortical hemorrhage-induced depression, but not sex differences. J Cereb Blood Flow Metab. 2021;41(12):3288-3301. doi: 10.1177/0271678X211029060. Epub 2021 Jul 8. PMID: 34238051; PMCID: PMC8669278.	*Off-target*
71	Romero-Miguel D, Casquero-Veiga M, MacDowell KS, Torres-Sanchez S, Garcia- Partida JA, Lamanna-Rama N, Romero-Miranda A, Berrocoso E, Leza JC, Desco M, Soto-Montenegro ML. A Characterization of the effects of minocycline treatment during adolescence on structural, metabolic, and oxidative stress parameters in a maternal immune stimulation model of neurodevelopmental brain disorders. Int J Neuropsychopharmacol. 2021;24(9):734-748. doi: 10.1093/ijnp/pyab036. PMID: 34165516; PMCID: PMC8453277.	*Off-target*
72	**Yang S, Yang Y, Chen C, Wang H, Ai Q, Lin M, Zeng Q, Zhang Y, Gao Y, Li X, Chen N. The anti-neuroinflammatory effect of fuzi and ganjiang extraction on LPS-induced BV2 microglia and its intervention function on depression-like behavior of cancer-related fatigue model mice. Front Pharmacol. 2021;12:670586. doi: 10.3389/fphar.2021.670586. PMID: 34122094; PMCID: PMC8193093.**	**Depression**
73	**Naß J, Abdelfatah S, Efferth T. The triterpenoid ursolic acid ameliorates stress in *Caenorhabditis elegans* by affecting the depression-associated genes skn-1 and prdx2. Phytomedicine. 2021;88:153598. doi: 10.1016/j.phymed.2021.153598. Epub 2021 May 23. PMID: 34111615.**	**Depression**
74	Tendilla-Beltrán H, Flores G. Due to their anti-inflammatory, antioxidant and neurotrophic properties, second-generation antipsychotics are suitable in patients with schizophrenia and COVID-19. Gen Hosp Psychiatry. 2021;71:137-139. doi: 10.1016/j.genhosppsych.2021.05.005. Epub 2021 May 23. PMID: 34045093; PMCID: PMC8141345.	Opinion
75	**Salama A, Mahmoud HA, Kandeil MA, Khalaf MM. Neuroprotective role of camphor against ciprofloxacin induced depression in rats: modulation of Nrf-2 and TLR4. Immunopharmacol Immunotoxicol. 2021;43(3):309-318. doi: 10.1080/08923973.2021.1905658. Epub 2021 May 25. PMID: 34032546.**	**Depression**
76	Hou X, Liu H, Ping Y, Zhang F, Zhi L, Jiang X, Zhang F, Song C, Zhang Z, Song J. CDDO-Im exerts antidepressant-like effects via the Nrf2/ARE pathway in a rat model of post-stroke depression. Brain Res Bull. 2021;173:74-81. doi: 10.1016/j.brainresbull.2021.05.008. Epub 2021 May 12. PMID: 33991607.	*Off-target*
77	Li X, Pan J, Wei Y, Ni L, Xu B, Deng Y, Yang T, Liu W. Mechanisms of oxidative stress in methylmercury-induced neurodevelopmental toxicity. Neurotoxicology. 2021;85:33-46. doi: 10.1016/j.neuro.2021.05.002. Epub 2021 May 6. PMID: 33964343.	*Off-target*
78	Hu X, Li R, Sun M, Kong Y, Zhu H, Wang F, Wan Q. Isovitexin depresses osteoarthritis progression *via* the Nrf2/NF-κB pathway: An *in vitro* study. J Inflamm Res. 2021;14:1403-1414. doi: 10.2147/JIR.S299557. PMID: 33883918; PMCID: PMC8053716.	*Off-target*
79	Naeem K, Tariq Al Kury L, Nasar F, Alattar A, Alshaman R, Shah FA, Khan AU, Li S. Natural dietary supplement, carvacrol, alleviates LPS-induced oxidative stress, neurodegeneration, and depressive-like behaviors *via* the Nrf2/HO-1 pathway. J Inflamm Res. 2021;14:1313-1329. doi: 10.2147/JIR.S294413. Retraction in: J Inflamm Res. 2022;15:6071-6072. PMID: 33854358; PMCID: PMC8041651.	Retracted
80	Hammad AM, Swiss GMS, Hall FS, Hikmat S, Sari Y, Al-Qirim TM, Amawi HA. Ceftriaxone reduces waterpipe tobacco smoke withdrawal-induced anxiety in rats via modulating the expression of TNF-α/NFκB, Nrf2, and GLT-1. Neuroscience. 2021;463:128-142. doi: 10.1016/j.neuroscience.2021.03.030. Epub 2021 Apr 6. PMID: 33836247.	Anxiety
81	Casquero-Veiga M, Romero-Miguel D, MacDowell KS, Torres-Sanchez S, Garcia- Partida JA, Lamanna-Rama N, Gómez-Rangel V, Romero-Miranda A, Berrocoso E, Leza JC, Arango C, Desco M, Soto-Montenegro ML. Omega-3 fatty acids during adolescence prevent schizophrenia-related behavioural deficits: Neurophysiological evidences from the prenatal viral infection with PolyI:C. Eur Neuropsychopharmacol. 2021;46:14-27. doi: 10.1016/j.euroneuro.2021.02.001. Epub 2021 Mar 15. PMID: 33735708.	SZ
82	Proskurnina EV, Sokolova SV, Ershova ES, Martynov AV, Portnova GV, Kostyuk SV, Zakharova NV, Kostyuk GP. Антиоксидантный статус плазмы крови пациентов с острым психозом и его связь с активацией транскрипционного фактора Nrf2 [Antioxidant status of blood plasma of acutely psychotic patients and its correlation with Nrf2 activation]. Zh Nevrol Psikhiatr Im S S Korsakova. 2021;121(2):60-66. Russian. doi: 10.17116/jnevro202112102160. PMID: 33728852.	SZ
83	**Yao W, Lin S, Su J, Cao Q, Chen Y, Chen J, Zhang Z, Hashimoto K, Qi Q, Zhang JC. Activation of BDNF by transcription factor Nrf2 contributes to antidepressant-like actions in rodents. Transl Psychiatry. 2021;11(1):140. doi: 10.1038/s41398-021-01261-6. PMID: 33627628; PMCID: PMC7904924.**	**Depression**
84	**Herbet M, Szumełda I, Piątkowska-Chmiel I, Gawrońska-Grzywacz M, Dudka J. Beneficial effects of combined administration of fluoxetine and mitochondria- targeted antioxidant at in behavioural and molecular studies in mice model of depression. Behav Brain Res. 2021;405:113185. doi: 10.1016/j.bbr.2021.113185. Epub 2021 Feb 19. PMID: 33617903.**	**Depression**
85	Bhandari R, Kaur J, Kaur S, Kuhad A. The Nrf2 pathway in psychiatric disorders: pathophysiological role and potential targeting. Expert Opin Ther Targets. 2021;25(2):115-139. doi: 10.1080/14728222.2021.1887141. Epub 2021 Mar 14. PMID: 33557652.	Review
86	Ermakov EA, Dmitrieva EM, Parshukova DA, Kazantseva DV, Vasilieva AR, Smirnova LP. Oxidative stress-related mechanisms in schizophrenia pathogenesis and new treatment perspectives. Oxid Med Cell Longev. 2021;2021:8881770. doi: 10.1155/2021/8881770. PMID: 33552387; PMCID: PMC7847339.	Review
87	Wang J, Wang S, Guo H, Li Y, Jiang Z, Gu T, Su B, Hou W, Zhong H, Cheng D, Zhang X, Fang Z. Rosmarinic acid protects rats against post-stroke depression after transient focal cerebral ischemic injury through enhancing antioxidant response. Brain Res. 2021;1757:147336. doi: 10.1016/j.brainres.2021.147336. Epub 2021 Feb 4. PMID: 33548269.	*Off-target*
88	Nadeem A, Ahmad SF, Al-Harbi NO, Attia SM, Bakheet SA, Alsanea S, Ali N, Albekairi TH, Alsaleh NB. Aggravation of autism-like behavior in BTBR T+tf/J mice by environmental pollutant, di-(2-ethylhexyl) phthalate: Role of nuclear factor erythroid 2-related factor 2 and oxidative enzymes in innate immune cells and cerebellum. Int Immunopharmacol. 2021;91:107323. doi: 10.1016/j.intimp.2020.107323. Epub 2020 Dec 29. PMID: 33385713.	Autism

89	Ayaydin H, Akaltun İ, Koyuncu İ, ÇelİK H, KİrmİT A, Takatak H. High KEAP1, NRF2 and low HO-1 serum levels in children with autism. Nörö Psikiyatr Arş. 2020;57(4):274-279. doi: 10.29399/npa.24862. PMID: 33354117; PMCID: PMC7735139.	Autism
90	Yang J, Fu X, Liao X, Li Y. Nrf2 activators as dietary phytochemicals against oxidative stress, inflammation, and mitochondrial dysfunction in autism spectrum disorders: A systematic review. Front Psychiatry. 2020;11:561998. doi: 10.3389/fpsyt.2020.561998. PMID: 33329102; PMCID: PMC7714765.	Review
91	Liang Y, Yu H, Ke X, Eyles D, Sun R, Wang Z, Huang S, Lin L, McGrath JJ, Lu J, Guo X, Yao P. Vitamin D deficiency worsens maternal diabetes induced neurodevelopmental disorder by potentiating hyperglycemia-mediated epigenetic changes. Ann N Y Acad Sci. 2021;1491(1):74-88. doi: 10.1111/nyas.14535. Epub 2020 Dec 10. PMID: 33305416.	*Off-target*
92	Cheng J, Zhang M, Cheng S, Li F, Zhang B, Sun X, Hu H, Chen L, Zhao Z, Hu H, Zhang Z. Low-dose alcohol ameliorated high fat diet-induced anxiety-related behavior *via* enhancing adiponectin expression and activating the Nrf2 pathway. Food Funct. 2021;12(1):241-251. doi: 10.1039/d0fo02704a. Epub 2020 Dec 9. PMID: 33295905.	Anxiety
93	Pereira AC, Oliveira J, Silva S, Madeira N, Pereira CMF, Cruz MT. Inflammation in Bipolar Disorder (BD): Identification of new therapeutic targets. Pharmacol Res. 2021;163:105325. doi: 10.1016/j.phrs.2020.105325. Epub 2020 Dec 2. PMID: 33278569.	BD
94	Tendilla-Beltrán H, Sanchez-Islas NDC, Marina-Ramos M, Leza JC, Flores G. The prefrontal cortex as a target for atypical antipsychotics in schizophrenia, lessons of neurodevelopmental animal models. Prog Neurobiol. 2021;199:101967. doi: 10.1016/j.pneurobio.2020.101967. Epub 2020 Nov 30. PMID: 33271238.	SZ
95	**Yan T, Li F, Xiong W, Wu B, Xiao F, He B, Jia Y. Nootkatone improves anxiety- and depression-like behavior by targeting hyperammonemia-induced oxidative stress in D-galactosamine model of liver injury. Environ Toxicol. 2021;36(4):694-706. doi: 10.1002/tox.23073. Epub 2020 Dec 3. PMID: 33270352.**	**Depression**, Anxiety
96	**Li W, Ali T, He K, Liu Z, Shah FA, Ren Q, Liu Y, Jiang A, Li S. Ibrutinib alleviates LPS-induced neuroinflammation and synaptic defects in a mouse model of depression. Brain Behav Immun. 2021;92:10-24. doi: 10.1016/j.bbi.2020.11.008. Epub 2020 Nov 10. PMID: 33181270.**	**Depression**
97	**Qu Y, Shan J, Wang S, Chang L, Pu Y, Wang X, Tan Y, Yamamoto M, Hashimoto K. Rapid-acting and long-lasting antidepressant-like action of (R)-ketamine in Nrf2 knock-out mice: a role of TrkB signaling. Eur Arch Psychiatry Clin Neurosci. 2021;271(3):439-446. doi: 10.1007/s00406-020-01208-w. Epub 2020 Nov 12. PMID: 33180200.**	**Depression**
98	**Liao D, Lv C, Cao L, Yao D, Wu Y, Long M, Liu N, Jiang P. Curcumin attenuates chronic unpredictable mild stress-induced depressive-like behaviors via restoring changes in oxidative stress and the activation of Nrf2 signaling pathway in rats. Oxid Med Cell Longev. 2020 Sep 17;2020:9268083. doi: 10.1155/2020/9268083. PMID: 33014280; PMCID: PMC7520007.**	**Depression**
99	**Zhu X, Liu H, Liu Y, Chen Y, Liu Y, Yin X. The Antidepressant-Like Effects of Hesperidin in Streptozotocin-Induced Diabetic Rats by Activating Nrf2/ARE/Glyoxalase 1 Pathway. Front Pharmacol. 2020 Aug 28;11:1325. doi: 10.3389/fphar.2020.01325. PMID: 32982741; PMCID: PMC7485173.**	**Depression**
100	Ma S, Guo Y, Sun L, Fan W, Liu Y, Liu D, Huang D, Li X, Zhang W, Mai K. Over high or low dietary protein levels depressed the growth, TOR signaling, apoptosis, immune and anti-stress of abalone Haliotis discus hannai. Fish Shellfish Immunol. 2020;106:241-251. doi: 10.1016/j.fsi.2020.08.004. Epub 2020 Aug 8. PMID: 32781210.	*Off-target*
101	**Park BK, Kim NS, Kim YR, Yang C, Jung IC, Jang IS, Seo CS, Choi JJ, Lee MY. Antidepressant and Anti-Neuroinflammatory Effects of Bangpungtongsung-San. Front Pharmacol. 2020 Jul 10;11:958. doi: 10.3389/fphar.2020.00958. PMID: 32754030; PMCID: PMC7366903.**	**Depression**
102	**Camargo A, Dalmagro AP, M Rosa J, B Zeni AL, P Kaster M, Tasca CI, S Rodrigues AL. Subthreshold doses of guanosine plus ketamine elicit antidepressant-like effect in a mouse model of depression induced by corticosterone: Role of GR/NF-κB/IDO-1 signaling. Neurochem Int. 2020;139:104797. doi: 10.1016/j.neuint.2020.104797. Epub 2020 Jul 9. PMID: 32652267.**	**Depression**
103	**Ali T, Hao Q, Ullah N, Rahman SU, Shah FA, He K, Zheng C, Li W, Murtaza I, Li Y, Jiang Y, Tan Z, Li S. Melatonin act as an antidepressant *via* attenuation of neuroinflammation by targeting Sirt1/Nrf2/HO-1 signaling. Front Mol Neurosci. 2020;13:96. doi: 10.3389/fnmol.2020.00096. PMID: 32595452; PMCID: PMC7304371.**	**Depression**
104	**Severo L, Godinho D, Machado F, Hartmann D, Fighera MR, Soares FA, Furian AF, Oliveira MS, Royes LF. The role of mitochondrial bioenergetics and oxidative stress in depressive behavior in recurrent concussion model in mice. Life Sci. 2020;257:117991. doi: 10.1016/j.lfs.2020.117991. Epub 2020 Jun 20. PMID: 32569782.**	**Depression**
105	Perkins DO, Jeffries CD, Do KQ. Potential roles of redox dysregulation in the development of schizophrenia. Biol Psychiatry. 2020;88(4):326-336. doi: 10.1016/j.biopsych.2020.03.016. Epub 2020 Apr 2. PMID: 32560962; PMCID: PMC7395886.	Review
106	**Liao D, Chen Y, Guo Y, Wang C, Liu N, Gong Q, Fu Y, Fu Y, Cao L, Yao D, Jiang P. Salvianolic acid B improves chronic mild stress-induced depressive behaviors in rats: Involvement of AMPK/SIRT1 signaling pathway. J Inflamm Res. 2020;13:195-206. doi: 10.2147/JIR.S249363. PMID: 32494183; PMCID: PMC7231775.**	**Depression**
107	**Wang W, Zheng L, Xu L, Tu J, Gu X. Pinocembrin mitigates depressive-like behaviors induced by chronic unpredictable mild stress through ameliorating neuroinflammation and apoptosis. Mol Med. 2020;26(1):53. doi: 10.1186/s10020-020-00179-x. PMID: 32460706; PMCID: PMC7251698.**	**Depression**
108	Wang W, Yang L, Liu T, Wang J, Wen A, Ding Y. Ellagic acid protects mice against sleep deprivation-induced memory impairment and anxiety by inhibiting TLR4 and activating Nrf2. Aging (Albany NY). 2020;12(11):10457-10472. doi: 10.18632/aging.103270. Epub 2020 May 20. PMID: 32433038; PMCID: PMC7346043.	Insomnia
109	**Kubick N, Pajares M, Enache I, Manda G, Mickael ME. Repurposing zileuton as a depression drug using an AI and *in vitro* approach. Molecules. 2020;25(9):2155. doi: 10.3390/molecules25092155. PMID: 32380663; PMCID: PMC7249014.**	**Depression**

110	**Ali T, Rahman SU, Hao Q, Li W, Liu Z, Ali Shah F, Murtaza I, Zhang Z, Yang X, Liu G, Li S. Melatonin prevents neuroinflammation and relieves depression by attenuating autophagy impairment through FOXO3a regulation. J Pineal Res. 2020;69(2):e12667. doi: 10.1111/jpi.12667. Epub 2020 Jul 17. PMID: 32375205.**	**Depression**
111	**Nakayama T, Okimura K, Shen J, Guh YJ, Tamai TK, Shimada A, Minou S, Okushi Y, Shimmura T, Furukawa Y, Kadofusa N, Sato A, Nishimura T, Tanaka M, Nakayama K, Shiina N, Yamamoto N, Loudon AS, Nishiwaki-Ohkawa T, Shinomiya A, Nabeshima T, Nakane Y, Yoshimura T. Seasonal changes in NRF2 antioxidant pathway regulates winter depression-like behavior. Proc Natl Acad Sci U S A. 2020;117(17):9594-9603. doi: 10.1073/pnas.2000278117. Epub 2020 Apr 10. PMID: 32277035; PMCID: PMC7196813.**	**Depression**
112	Zhang L, Zhang J, Yan E, He J, Zhong X, Zhang L, Wang C, Wang T. Dietary Supplemented Curcumin Improves Meat Quality and Antioxidant Status of Intrauterine Growth Retardation Growing Pigs via Nrf2 Signal Pathway. Animals (Basel). 2020;10(3):539. doi: 10.3390/ani10030539. PMID: 32213933; PMCID: PMC7143559.	*Off-target*
113	**Li T, Zheng LN, Han XH. Fenretinide attenuates lipopolysaccharide (LPS)-induced blood-brain barrier (BBB) and depressive-like behavior in mice by targeting Nrf-2 signaling. Biomed Pharmacother. 2020;125:109680. doi: 10.1016/j.biopha.2019.109680. Epub 2020 Feb 25. PMID: 32106372.**	**Depression**
114	Alvarez-Arellano L, González-García N, Salazar-García M, Corona JC. Antioxidants as a Potential Target against Inflammation and Oxidative Stress in Attention-Deficit/Hyperactivity Disorder. Antioxidants (Basel). 2020;9(2):176. doi: 10.3390/antiox9020176. PMID: 32098021; PMCID: PMC7070894.	ADHD
115	Cai M, Hu JY, Liu BB, Li JJ, Li F, Lou S. The molecular mechanisms of excessive hippocampal endoplasmic reticulum stress depressing cognition-related proteins expression and the regulatory effects of Nrf2. Neuroscience. 2020;431:152-165. doi: 10.1016/j.neuroscience.2020.02.001. Epub 2020 Feb 14. PMID: 32062019.	*Off-target*
116	Bhandari R, Paliwal JK, Kuhad A. Dietary Phytochemicals as Neurotherapeutics for Autism Spectrum Disorder: Plausible Mechanism and Evidence. Adv Neurobiol. 2020;24:615-646. doi: 10.1007/978-3-030-30402-7_23. PMID: 32006377.	Autism
117	**Tian L, Sun SS, Cui LB, Wang SQ, Peng ZW, Tan QR, Hou WG, Cai M. Repetitive transcranial magnetic stimulation elicits antidepressant- and anxiolytic-like effect via nuclear factor-E2-related factor 2-mediated anti-inflammation mechanism in rats. Neuroscience. 2020;429:119-133. doi: 10.1016/j.neuroscience.2019.12.025. Epub 2020 Jan 7. PMID: 31918011.**	**Depression**, Anxiety
118	Nadeem A, Ahmad SF, Al-Ayadhi LY, Attia SM, Al-Harbi NO, Alzahrani KS, Bakheet SA. Differential regulation of Nrf2 is linked to elevated inflammation and nitrative stress in monocytes of children with autism. Psychoneuroendocrinology. 2020;113:104554. doi: 10.1016/j.psyneuen.2019.104554. Epub 2019 Dec 23. PMID: 31884317.	Autism
119	Zhao Y, Niu Y, He J, Zhang L, Wang C, Wang T. Dietary dihydroartemisinin supplementation attenuates hepatic oxidative damage of weaned piglets with intrauterine growth retardation through the Nrf2/ARE signaling pathway. Animals (Basel). 2019;9(12):1144. doi: 10.3390/ani9121144. PMID: 31847280; PMCID: PMC6941019.	*Off-target*
120	**Casaril AM, Domingues M, Bampi SR, Lourenço DA, Smaniotto TÂ, Segatto N, Vieira B, Seixas FK, Collares T, Lenardão EJ, Savegnago L. The antioxidant and immunomodulatory compound 3-[(4-chlorophenyl)selanyl]-1-methyl-1H-indole attenuates depression-like behavior and cognitive impairment developed in a mouse model of breast tumor. Brain Behav Immun. 2020;84:229-241. doi: 10.1016/j.bbi.2019.12.005. Epub 2019 Dec 16. PMID: 31837417.**	**Depression**
121	**Zborowski VA, Heck SO, Vencato M, Pinton S, Marques LS, Nogueira CW. Keap1/Nrf2/HO-1 signaling pathway contributes to p-chlorodiphenyl diselenide antidepressant-like action in diabetic mice. Psychopharmacology (Berl). 2020;237(2):363-374. doi: 10.1007/s00213-019-05372-3. Epub 2019 Dec 11. PMID: 31828396.**	**Depression**
122	Niu Y, He J, Ahmad H, Shen M, Zhao Y, Gan Z, Zhang L, Zhong X, Wang C, Wang T. Dietary curcumin supplementation increases antioxidant capacity, upregulates Nrf2 and Hmox1 levels in the liver of piglet model with intrauterine growth retardation. Nutrients. 2019;11(12):2978. doi: 10.3390/nu11122978. PMID: 31817533; PMCID: PMC6950043.	*Off-target*
123	**Huang X, Fei GQ, Liu WJ, Ding J, Wang Y, Wang H, Ji JL, Wang X. Adipose- derived mesenchymal stem cells protect against CMS-induced depression-like behaviors in mice via regulating the Nrf2/HO-1 and TLR4/NF-κB signaling pathways. Acta Pharmacol Sin. 2020;41(5):612-619. doi: 10.1038/s41401-019-0317-6. Epub 2019 Dec 3. PMID: 31796867; PMCID: PMC7468309.**	**Depression**
124	**Rosa PB, Bettio LEB, Neis VB, Moretti M, Werle I, Leal RB, Rodrigues ALS. The antidepressant-like effect of guanosine is dependent on GSK-3β inhibition and activation of MAPK/ERK and Nrf2/heme oxygenase-1 signaling pathways. Purinergic Signal. 2019;15(4):491-504. doi: 10.1007/s11302-019-09681-2. Epub 2019 Nov 25. PMID: 31768875; PMCID: PMC6923309.**	**Depression**
125	Mammana S, Cavalli E, Gugliandolo A, Silvestro S, Pollastro F, Bramanti P, Mazzon E. Could the Combination of Two Non-Psychotropic Cannabinoids Counteract Neuroinflammation? Effectiveness of Cannabidiol Associated with Cannabigerol. Medicina (Kaunas). 2019;55(11):747. doi: 10.3390/medicina55110747. PMID: 31752240; PMCID: PMC6915685.	*Off-target*
126	Xie J, Fang H, Liao S, Guo T, Yin P, Liu Y, Tian L, Niu J. Study on *Schizochytrium sp.* improving the growth performance and non-specific immunity of golden pompano *(Trachinotus ovatus)* while not affecting the antioxidant capacity. Fish Shellfish Immunol. 2019;95:617-623. doi: 10.1016/j.fsi.2019.10.028. Epub 2019 Oct 14. PMID: 31622676.	*Off-target*
127	**Liu H, Jiang J, Zhao L. Protein arginine methyltransferase-1 deficiency restrains depression-like behavior of mice by inhibiting inflammation and oxidative stress via Nrf-2. Biochem Biophys Res Commun. 2019;518(3):430-437. doi: 10.1016/j.bbrc.2019.08.032. Epub 2019 Sep 3. PMID: 31492498.**	**Depression**

128	**Cigliano L, Spagnuolo MS, Boscaino F, Ferrandino I, Monaco A, Capriello T, Cocca E, Iannotta L, Treppiccione L, Luongo D, Maurano F, Rossi M, Bergamo P. Dietary supplementation with fish oil or conjugated linoleic acid relieves depression markers in mice by modulation of the Nrf2 pathway. Mol Nutr Food Res. 2019;63(21):e1900243. doi: 10.1002/mnfr.201900243. Epub 2019 Sep 2. PMID: 31398773.**	**Depression**
129	**Arioz BI, Tastan B, Tarakcioglu E, Tufekci KU, Olcum M, Ersoy N, Bagriyanik A, Genc K, Genc S. Melatonin attenuates LPS-induced acute depressive-like behaviors and microglial NLRP3 inflammasome activation through the SIRT1/Nrf2 pathway. Front Immunol. 2019;10:1511. doi: 10.3389/fimmu.2019.01511. PMID: 31327964; PMCID: PMC6615259.**	**Depression**
130	Hashimoto K. Recent advances in the early intervention in schizophrenia: Future direction from preclinical findings. Curr Psychiatry Rep. 2019;21(8):75. doi: 10.1007/s11920-019-1063-7. PMID: 31278495.	Review
131	Díaz AF, Polo S, Gallardo N, Leánez S, Pol O. Analgesic and antidepressant effects of oltipraz on neuropathic pain in mice by modulating microglial activation. J Clin Med. 2019;8(6):890. doi: 10.3390/jcm8060890. PMID: 31234342; PMCID: PMC6616658.	*Off-target*
132	Horigome Y, Ida-Yonemochi H, Waguri S, Shibata S, Endo N, Komatsu M. Loss of autophagy in chondrocytes causes severe growth retardation. Autophagy. 2020;16(3):501-511. doi: 10.1080/15548627.2019.1628541. Epub 2019 Jun 16. PMID: 31203752; PMCID: PMC6999621.	*Off-target*
133	**Gao W, Wang W, Zhang J, Deng P, Hu J, Yang J, Deng Z. Allicin ameliorates obesity comorbid depressive-like behaviors: involvement of the oxidative stress, mitochondrial function, autophagy, insulin resistance and NOX/Nrf2 imbalance in mice. Metab Brain Dis. 2019;34(5):1267-1280. doi: 10.1007/s11011-019-00443-y. Epub 2019 Jun 14. PMID: 31201726.**	**Depression**
134	Bansal Y, Singh R, Parhar I, Kuhad A, Soga T. Quinolinic acid and nuclear factor erythroid 2-related factor 2 in depression: Role in neuroprogression. Front Pharmacol. 2019;10:452. doi: 10.3389/fphar.2019.00452. PMID: 31164818; PMCID: PMC6536572.	Review
135	**Dang R, Guo YY, Zhang K, Jiang P, Zhao MG. Predictable chronic mild stress promotes recovery from LPS-induced depression. Mol Brain. 2019;12(1):42. doi: 10.1186/s13041-019-0463-2. PMID: 31053149; PMCID: PMC6500057.**	**Depression**
136	Wang Z, Liang M, Li H, Cai L, He H, Wu Q, Yang L. l-Methionine activates Nrf2-ARE pathway to induce endogenous antioxidant activity for depressing ROS-derived oxidative stress in growing rats. J Sci Food Agric. 2019;99(10):4849-4862. doi: 10.1002/jsfa.9757. Epub 2019 May 13. PMID: 31001831.	*Off-target*
137	Li XM, Rong H, Qian JY, Dong MX, Niu YC. 孕期母亲暴露于姜黄对后代神经发育和凋亡机制的影响 [Effect of maternal exposure to *Curcumae Rhizoma* during pregnancy on neurodevelopment and apoptosis mechanism in offspring]. Zhongguo Zhong Yao Za Zhi. 2019;44(3):541-545. Chinese. doi: 10.19540/j.cnki.cjcmm.20181012.004. PMID: 30989920.	*Off-target*
138	Sharma N, Shin EJ, Kim NH, Cho EH, Nguyen BT, Jeong JH, Jang CG, Nah SY, Kim HC. Far-infrared ray-mediated antioxidant potentials are important for attenuating psychotoxic disorders. Curr Neuropharmacol. 2019;17(10):990-1002. doi: 10.2174/1570159X17666190228114318. PMID: 30819085; PMCID: PMC7052827.	*Off-target*
139	Nadeem A, Ahmad SF, Al-Harbi NO, Attia SM, Bakheet SA, Ibrahim KE, Alqahtani F, Alqinyah M. Nrf2 activator, sulforaphane ameliorates autism-like symptoms through suppression of Th17 related signaling and rectification of oxidant-antioxidant imbalance in periphery and brain of BTBR T+tf/J mice. Behav Brain Res. 2019;364:213-224. doi: 10.1016/j.bbr.2019.02.031. Epub 2019 Feb 19. PMID: 30790585.	Autism
140	**Chu C, Zhang H, Cui S, Han B, Zhou L, Zhang N, Su X, Niu Y, Chen W, Chen R, Zhang R, Zheng Y. Ambient PM2.5 caused depressive-like responses through Nrf2/NLRP3 signaling pathway modulating inflammation. J Hazard Mater. 2019;369:180-190. doi: 10.1016/j.jhazmat.2019.02.026. Epub 2019 Feb 10. PMID: 30776601.**	**Depression**
141	Wu Y, Wang L, Hu K, Yu C, Zhu Y, Zhang S, Shao A. Mechanisms and therapeutic targets of depression after intracerebral hemorrhage. Front Psychiatry. 2018;9:682. doi: 10.3389/fpsyt.2018.00682. PMID: 30618863; PMCID: PMC6304443.	*Off-target*
142	**Fan C, Song Q, Wang P, Li Y, Yang M, Yu SY. Neuroprotective effects of ginsenoside-Rg1 against depression-like behaviors via suppressing glial activation, synaptic deficits, and neuronal apoptosis in rats. Front Immunol. 2018;9:2889. doi: 10.3389/fimmu.2018.02889. PMID: 30581440; PMCID: PMC6292928.**	**Depression**
143	Ferreira-Chamorro P, Redondo A, Riego G, Leánez S, Pol O. Sulforaphane inhibited the nociceptive responses, anxiety- and depressive-like behaviors associated with neuropathic pain and improved the anti-allodynic effects of morphine in mice. Front Pharmacol. 2018;9:1332. doi: 10.3389/fphar.2018.01332. PMID: 30542282; PMCID: PMC6277937.	*Off-target*
144	**Gao W, Wang W, Liu G, Zhang J, Yang J, Deng Z. Allicin attenuated chronic social defeat stress induced depressive-like behaviors through suppression of NLRP3 inflammasome. Metab Brain Dis. 2019;34(1):319-329. doi: 10.1007/s11011-018-0342-z. Epub 2018 Dec 4. PMID: 30515710.**	**Depression**
145	Hashimoto K. Essential role of Keap1-Nrf2 signaling in mood disorders: Overview and future perspective. Front Pharmacol. 2018;9:1182. doi: 10.3389/fphar.2018.01182. PMID: 30386243; PMCID: PMC6198170.	Review
146	**Martín-Hernández D, Caso JR, Javier Meana J, Callado LF, Madrigal JLM, García-Bueno B, Leza JC. Intracellular inflammatory and antioxidant pathways in postmortem frontal cortex of subjects with major depression: effect of antidepressants. J Neuroinflammation. 2018;15(1):251. doi: 10.1186/s12974-018-1294-2. PMID: 30180869; PMCID: PMC6122627.**	**Depression**
147	Bolotta A, Visconti P, Fedrizzi G, Ghezzo A, Marini M, Manunta P, Messaggio E, Posar A, Vignini A, Abruzzo PM. Na^+, K^+-ATPase activity in children with autism spectrum disorder: Searching for the reason(s) of its decrease in blood cells. Autism Res. 2018;11(10):1388-1403. doi: 10.1002/aur.2002. Epub 2018 Aug 18. PMID: 30120881; PMCID: PMC6221099.	Autism

148	Tian H, Sun W, Zhang Q, Li X, Sang Y, Li J, Niu Y, Ding H. Procyanidin B2 mitigates behavioral impairment and protects myelin integrity in cuprizone- induced schizophrenia in mice. RSC Adv. 2018;8(42):23835-23846. doi: 10.1039/c8ra03854f. PMID: 35540280; PMCID: PMC9081829.	SZ
149	**Yang M, Dang R, Xu P, Guo Y, Han W, Liao D, Jiang P. Dl-3-n-Butylphthalide improves lipopolysaccharide-induced depressive-like behavior in rats: involvement of Nrf2 and NF-κB pathways. Psychopharmacology (Berl). 2018;235(9):2573-2585. doi: 10.1007/s00213-018-4949-x. Epub 2018 Jun 25. PMID: 29943092.**	**Depression**
150	Gargouri B, Bhatia HS, Bouchard M, Fiebich BL, Fetoui H. Inflammatory and oxidative mechanisms potentiate bifenthrin-induced neurological alterations and anxiety-like behavior in adult rats. Toxicol Lett. 2018;294:73-86. doi: 10.1016/j.toxlet.2018.05.020. Epub 2018 May 26. PMID: 29775722.	Anxiety
151	Rosa JM, Pazini FL, Cunha MP, Colla ARS, Manosso LM, Mancini G, Souza ACG, de Bem AF, Prediger RD, Rodrigues ALS. Antidepressant effects of creatine on amyloid β_{1-40}-treated mice: The role of GSK-3β/Nrf_2 pathway. Prog Neuropsychopharmacol Biol Psychiatry. 2018;86:270-278. doi: 10.1016/j.pnpbp.2018.05.001. Epub 2018 May 9. PMID: 29753049.	*Off-target*
152	Fedoce AdG, Ferreira F, Bota RG, Bonet-Costa V, Sun PY, Davies KJA. The role of oxidative stress in anxiety disorder: cause or consequence? Free Radic Res. 2018;52(7):737-750. doi: 10.1080/10715762.2018.1475733. Epub 2018 Jun 4. PMID: 29742940; PMCID: PMC6218334.	Review
153	Ishtiaq SM, Khan JA, Arshad MI. Psychosocial-stress, liver regeneration and weight gain: a conspicuous pathophysiological triad. Cell Physiol Biochem. 2018;46(1):1-8. doi: 10.1159/000488378. Epub 2018 Mar 20. PMID: 29566366.	Review
154	Dong YW, Feng L, Jiang WD, Liu Y, Wu P, Jiang J, Kuang SY, Tang L, Tang WN, Zhang YA, Zhou XQ. Dietary threonine deficiency depressed the disease resistance, immune and physical barriers in the gills of juvenile grass carp *(Ctenopharyngodon idella)* under infection of *Flavobacterium columnare*. Fish Shellfish Immunol. 2018;72:161-173. doi: 10.1016/j.fsi.2017.10.048. Epub 2017 Oct 31. PMID: 29100986.	*Off-target*
155	**Li M, Li C, Yu H, Cai X, Shen X, Sun X, Wang J, Zhang Y, Wang C. Lentivirus-mediated interleukin-1β (IL-1β) knock-down in the hippocampus alleviates lipopolysaccharide (LPS)-induced memory deficits and anxiety- and depression-like behaviors in mice. J Neuroinflammation. 2017;14(1):190. doi: 10.1186/s12974-017-0964-9. PMID: 28931410; PMCID: PMC5607621.**	**Depression**, Anxiety
156	**Omar NN, Tash RF. Fluoxetine coupled with zinc in a chronic mild stress model of depression: Providing a reservoir for optimum zinc signaling and neuronal remodeling. Pharmacol Biochem Behav. 2017;160:30-38. doi: 10.1016/j.pbb.2017.08.003. Epub 2017 Aug 9. PMID: 28801265.**	**Depression**
157	**Abuelezz SA, Hendawy N. Insights into the potential antidepressant mechanisms of cilostazol in chronically restraint rats: impact on the Nrf2 pathway. Behav Pharmacol. 2018;29(1):28-40. doi: 10.1097/FBP.0000000000000335. PMID: 28763303.**	**Depression**
158	**López-Granero C, Antunes Dos Santos A, Ferrer B, Culbreth M, Chakraborty S, Barrasa A, Gulinello M, Bowman AB, Aschner M. BXD recombinant inbred strains participate in social preference, anxiety and depression behaviors along sex- differences in cytokines and tactile allodynia. Psychoneuroendocrinology. 2017;80:92-98. doi: 10.1016/j.psyneuen.2017.03.006. Epub 2017 Mar 6. PMID: 28324704; PMCID: PMC5985819.**	**Depression**, Anxiety
159	Liu B, Teschemacher AG, Kasparov S. Astroglia as a cellular target for neuroprotection and treatment of neuro-psychiatric disorders. Glia. 2017;65(8):1205-1226. doi: 10.1002/glia.23136. Epub 2017 Mar 16. PMID: 28300322; PMCID: PMC5669250.	Review
160	**Zhao X-J, Zhao Z, Yang D-D, Cao L-L, Zhang L, Ji J, Gu J, Huang J-Y, Sun X-L. Activation of ATP-sensitive potassium channel by iptakalim normalizes stress- induced HPA axis disorder and depressive behaviour by alleviating inflammation and oxidative stress in mouse hypothalamus. Brain Res Bull. 2017;130:146-155. doi: 10.1016/j.brainresbull.2017.01.026. Epub 2017 Feb 1. PMID: 28161195.**	**Depression**
161	MacDowell KS, Munarriz-Cuezva E, Caso JR, Madrigal JL, Zabala A, Meana JJ, García-Bueno B, Leza JC. Paliperidone reverts Toll-like receptor 3 signaling pathway activation and cognitive deficits in a maternal immune activation mouse model of schizophrenia. Neuropharmacology. 2017;116:196-207. doi: 10.1016/j.neuropharm.2016.12.025. Epub 2016 Dec 28. PMID: 28039001.	SZ
162	Pan JH, Feng L, Jiang WD, Wu P, Kuang SY, Tang L, Zhang YA, Zhou XQ, Liu Y. Vitamin E deficiency depressed fish growth, disease resistance, and the immunity and structural integrity of immune organs in grass carp *(Ctenopharyngodon idella)*: Referring to NF-κB, TOR and Nrf2 signaling. Fish Shellfish Immunol. 2017;60:219-236. doi: 10.1016/j.fsi.2016.11.044. Epub 2016 Nov 22. PMID: 27888132.	*Off-target*
163	**Zhang J-c, Yao W, Dong C, Yang C, Ren Q, Ma M, Han M, Wu J, Ushida Y, Suganuma H, Hashimoto K. Prophylactic effects of sulforaphane on depression-like behavior and dendritic changes in mice after inflammation. J Nutr Biochem. 2017;39:134-144. doi: 10.1016/j.jnutbio.2016.10.004. Epub 2016 Oct 11. Erratum in: J Nutr Biochem. 2021;88:108550; J Nutr Biochem. 2021;89:108562. PMID: 27833054.**	**Depression**
164	**Yao W, Zhang J-C, Ishima T, Ren Q, Yang C, Dong C, Ma M, Saito A, Honda T, Hashimoto K. Antidepressant effects of TBE-31 and MCE-1, the novel Nrf2 activators, in an inflammation model of depression. Eur J Pharmacol. 2016;793:21-27. doi: 10.1016/j.ejphar.2016.10.037. Epub 2016 Nov 1. PMID: 27815170.**	**Depression**
165	Bouvier E, Brouillard F, Molet J, Claverie D, Cabungcal JH, Cresto N, Doligez N, Rivat C, Do KQ, Bernard C, Benoliel JJ, Becker C. Nrf2-dependent persistent oxidative stress results in stress-induced vulnerability to depression. Mol Psychiatry. 2017;22(12):1795. doi: 10.1038/mp.2016.211. Epub 2016 Nov 1. Erratum for: Mol Psychiatry. 2017;22(12):1701-1713. PMID: 27801891; PMCID: PMC8127815.	Duplicate=168
166	Oliveira CES, Marcondes Sari MHM, Zborowski VA, Prado VC, Nogueira CW, Zeni G. Pain-depression dyad induced by reserpine is relieved by p,p'-methoxyl-diphenyl diselenide in rats. Eur J Pharmacol. 2016;791:794-802. doi: 10.1016/j.ejphar.2016.10.021. Epub 2016 Oct 18. PMID: 27769701.	*Off-target*
167	Xu H-J, Jiang W-D, Feng L, Liu Y, Wu P, Jiang J, Kuang S-Y, Tang L, Tang W-N, Zhang Y-A, Zhou X-Q. Dietary vitamin C deficiency depressed the gill physical barriers and immune barriers referring to Nrf2, apoptosis, MLCK, NF-κB and TOR signaling in grass carp *(Ctenopharyngodon idella)* under infection of *Flavobacterium columnare*. Fish Shellfish Immunol. 2016;58:177-192. doi: 10.1016/j.fsi.2016.09.029. Epub 2016 Sep 15. PMID: 27640333.	*Off-target*

168	**Bouvier E, Brouillard F, Molet J, Claverie D, Cabungcal JH, Cresto N, Doligez N, Rivat C, Do KQ, Bernard C, Benoliel JJ, Becker C. Nrf2-dependent persistent oxidative stress results in stress-induced vulnerability to depression. Mol Psychiatry. 2017;22(12):1701-1713. doi: 10.1038/mp.2016.144. Epub 2016 Sep 20. Erratum in: Mol Psychiatry. 2017 Dec;22(12):1795. PMID: 27646262.**	**Depression**
169	Gureev AP, Syromyatnikov MY, Gorbacheva TM, Starkov AA, Popov VN. Methylene blue improves sensorimotor phenotype and decreases anxiety in parallel with activating brain mitochondria biogenesis in mid-age mice. Neurosci Res. 2016;113:19-27. doi: 10.1016/j.neures.2016.07.006. Epub 2016 Aug 8. PMID: 27515402.	Anxiety
170	**Yao W, Zhang J-c, Ishima T, Dong C, Yang C, Ren Q, Ma M, Han M, Wu J, Suganuma H, Ushida Y, Yamamoto M, Hashimoto K. Role of Keap1-Nrf2 signaling in depression and dietary intake of glucoraphanin confers stress resilience in mice. Sci Rep. 2016;6:30659. doi: 10.1038/srep30659. PMID: 27470577; PMCID: PMC4965765.**	**Depression**
171	Jiang W-D, Feng L, Qu B, Wu P, Kuang S-Y, Jiang J, Tang L, Tang W-N, Zhang Y-A, Zhou X-Q, Liu Y. Changes in integrity of the gill during histidine deficiency or excess due to depression of cellular anti-oxidative ability, induction of apoptosis, inflammation and impair of cell-cell tight junctions related to Nrf2, TOR and NF-κB signaling in fish. Fish Shellfish Immunol. 2016;56:111-122. doi: 10.1016/j.fsi.2016.07.002. Epub 2016 Jul 6. PMID: 27394967.	*Off-target*
172	Wells PG, Bhatia S, Drake DM, Miller-Pinsler L. Fetal oxidative stress mechanisms of neurodevelopmental deficits and exacerbation by ethanol and methamphetamine. Birth Defects Res C Embryo Today. 2016;108(2):108-130. doi: 10.1002/bdrc.21134. PMID: 27345013.	Review
173	**Mellon SH, Wolkowitz OM, Schonemann MD, Epel ES, Rosser R, Burke HB, Mahan L, Reus VI, Stamatiou D, Liew CC, Cole SW. Alterations in leukocyte transcriptional control pathway activity associated with major depressive disorder and antidepressant treatment. Transl Psychiatry. 2016;6(5):e821. doi: 10.1038/tp.2016.79. PMID: 27219347; PMCID: PMC5070063.**	**Depression**
174	Xu HJ, Jiang WD, Feng L, Liu Y, Wu P, Jiang J, Kuang SY, Tang L, Tang WN, Zhang YA, Zhou XQ. Dietary vitamin C deficiency depresses the growth, head kidney and spleen immunity and structural integrity by regulating NF-κB, TOR, Nrf2, apoptosis and MLCK signaling in young grass carp *(Ctenopharyngodon idella)*. Fish Shellfish Immunol. 2016;52:111-38. doi: 10.1016/j.fsi.2016.02.033. Epub 2016 Mar 2. PMID: 26944716.	*Off-target*
175	**Wojnicz A, Avendaño Ortiz J, Casas AI, Freitas AE, G López M, Ruiz-Nuño A. Simultaneous determination of 8 neurotransmitters and their metabolite levels in rat brain using liquid chromatography in tandem with mass spectrometry: Application to the murine Nrf2 model of depression. Clin Chim Acta. 2016;453:174-181. doi: 10.1016/j.cca.2015.12.023. Epub 2015 Dec 19. PMID: 26712273.**	**Depression**
176	**Martín-Hernández D, Caso JR, Bris ÁG, Maus SR, Madrigal JL, García-Bueno B, MacDowell KS, Alou L, Gómez-Lus ML, Leza JC. Bacterial translocation affects intracellular neuroinflammatory pathways in a depression-like model in rats. Neuropharmacology. 2016 Apr;103:122-133. doi: 10.1016/j.neuropharm.2015.12.003. Epub 2015 Dec 11. PMID: 26686392.**	**Depression**
177	**Martín-Hernández D, Bris ÁG, MacDowell KS, García-Bueno B, Madrigal JL, Leza JC, Caso JR. Modulation of the antioxidant nuclear factor (erythroid 2-derived)-like 2 pathway by antidepressants in rats. Neuropharmacology. 2016;103:79-91. doi: 10.1016/j.neuropharm.2015.11.029. Epub 2015 Dec 12. PMID: 26686388.**	**Depression**
178	Li L, Feng L, Jiang W-D, Jiang J, Wu P, Zhao J, Kuang S-Y, Tang L, Tang W-N, Zhang Y-A, Zhou X-Q, Liu Y. Dietary pantothenic acid depressed the gill immune and physical barrier function via NF-κB, TOR, Nrf2, p38MAPK and MLCK signaling pathways in grass carp *(Ctenopharyngodon idella)*. Fish Shellfish Immunol. 2015;47(1):500-510. doi: 10.1016/j.fsi.2015.09.038. Epub 2015 Sep 30. PMID: 26432048.	*Off-target*
179	Hermes-Lima M, Moreira DC, Rivera-Ingraham GA, Giraud-Billoud M, Genaro- Mattos TC, Campos ÉG. Preparation for oxidative stress under hypoxia and metabolic depression: Revisiting the proposal two decades later. Free Radic Biol Med. 2015;89:1122-1143. doi: 10.1016/j.freeradbiomed.2015.07.156. Epub 2015 Sep 25. PMID: 26408245.	Review
180	Jiang W-D, Hu K, Zhang J-X, Liu Y, Jiang J, Wu P, Zhao J, Kuang S-Y, Tang L, Tang W-N, Zhang Y-A, Zhou X-Q, Feng L. Soyabean glycinin depresses intestinal growth and function in juvenile Jian carp *(Cyprinus carpio* var Jian*)*: protective effects of glutamine. Br J Nutr. 2015;114(10):1569-1583. doi: 10.1017/S0007114515003219. Epub 2015 Sep 9. PMID: 26349522.	*Off-target*
181	Jiang W-D, Tang R-J, Liu Y, Kuang S-Y, Jiang J, Wu P, Zhao J, Zhang Y-A, Tang L, Tang W-N, Zhou X-Q, Feng L. Manganese deficiency or excess caused the depression of intestinal immunity, induction of inflammation and dysfunction of the intestinal physical barrier, as regulated by NF-κB, TOR and Nrf2 signalling, in grass carp *(Ctenopharyngodon idella)*. Fish Shellfish Immunol. 2015;46(2):406-416. doi: 10.1016/j.fsi.2015.06.007. Epub 2015 Jun 10. PMID: 26072140.	*Off-target*
182	**Freitas AE, Egea J, Buendia I, Gómez-Rangel V, Parada E, Navarro E, Casas AI, Wojnicz A, Ortiz JA, Cuadrado A, Ruiz-Nuño A, Rodrigues ALS, Lopez MG. Agmatine, by improving neuroplasticity markers and inducing Nrf2, prevents corticosterone-induced depressive-like behavior in mice. Mol Neurobiol. 2016;53(5):3030-3045. doi: 10.1007/s12035-015-9182-6. Epub 2015 May 13. PMID: 25966970.**	**Depression**
183	Li L, Feng L, Jiang W-D, Jiang J, Wu P, Kuang S-Y, Tang L, Tang W-N, Zhang Y-A, Zhou X-Q, Liu Y. Dietary pantothenic acid deficiency and excess depress the growth, intestinal mucosal immune and physical functions by regulating NF-κB, TOR, Nrf2 and MLCK signaling pathways in grass carp *(Ctenopharyngodon idella)*. Fish Shellfish Immunol. 2015;45(2):399-413. doi: 10.1016/j.fsi.2015.04.030. Epub 2015 May 6. PMID: 25957886.	*Off-target*
184	**Cunha MP, Budni J, Ludka FK, Pazini FL, Rosa JM, Oliveira Á, Lopes MW, Tasca CI, Leal RB, Rodrigues ALS. Involvement of PI3K/Akt signaling pathway and its downstream intracellular targets in the antidepressant-like effect of creatine. Mol Neurobiol. 2016;53(5):2954-2968. doi: 10.1007/s12035-015-9192-4. Epub 2015 May 6. PMID: 25943184.**	**Depression**

185	**Mendez-David I, Tritschler L, Ali ZE, Damiens MH, Pallardy M, David DJ, Kerdine-Römer S, Gardier AM. Nrf2-signaling and BDNF: A new target for the antidepressant-like activity of chronic fluoxetine treatment in a mouse model of anxiety/depression. Neurosci Lett. 2015;597:121-126. doi: 10.1016/j.neulet.2015.04.036. Epub 2015 Apr 24. PMID: 25916883.**	**Depression**, Anxiety
186	Bakunina N, Pariante CM, Zunszain PA. Immune mechanisms linked to depression via oxidative stress and neuroprogression. Immunology. 2015;144(3):365-373. doi: 10.1111/imm.12443. Epub 2015 Jan 10. PMID: 25580634; PMCID: PMC4557673.	Review
187	**Lukic I, Mitic M, Djordjevic J, Tatalovic N, Bozovic N, Soldatovic I, Mihaljevic M, Pavlovic Z, Radojcic MB, Maric NP, Adzic M. Lymphocyte levels of redox-sensitive transcription factors and antioxidative enzymes as indicators of pro-oxidative state in depressive patients. Neuropsychobiology. 2014;70(1):1-9. doi: 10.1159/000362841. Epub 2014 Aug 21. PMID: 25170744.**	**Depression**
188	Khalifeh S, Oryan S, Digaleh H, Shaerzadeh F, Khodagholi F, Maghsoudi N, Zarrindast MR. Involvement of Nrf2 in development of anxiety-like behavior by linking Bcl2 to oxidative phosphorylation: estimation in rat hippocampus, amygdala, and prefrontal cortex. J Mol Neurosci. 2015;55(2):492-499. doi: 10.1007/s12031-014-0370-z. Epub 2014 Jul 11. PMID: 25007950.	Anxiety
189	Napoli E, Wong S, Hertz-Picciotto I, Giulivi C. Deficits in bioenergetics and impaired immune response in granulocytes from children with autism. Pediatrics. 2014;133(5):e1405-e1410. doi: 10.1542/peds.2013-1545. PMID: 24753527; PMCID: PMC4006429.	Case
190	Stamova BS, Tian Y, Nordahl CW, Shen MD, Rogers S, Amaral DG, Sharp FR. Evidence for differential alternative splicing in blood of young boys with autism spectrum disorders. Mol Autism. 2013;4(1):30. doi: 10.1186/2040-2392-4-30. PMID: 24007566; PMCID: PMC3846739.	Autism
191	Ramkissoon A, Wells PG. Developmental role of nuclear factor E2-related factor 2 in mitigating methamphetamine fetal toxicity and postnatal neurodevelopmental deficits. Free Radic Biol Med. 2013;65:620-631. doi: 10.1016/j.freeradbiomed.2013.07.043. Epub 2013 Aug 7. PMID: 23932974.	*Off-target*
192	**Martín-de-Saavedra MD, Budni J, Cunha MP, Gómez-Rangel V, Lorrio S, Del Barrio L, Lastres-Becker I, Parada E, Tordera RM, Rodrigues AL, Cuadrado A, López MG. Nrf2 participates in depressive disorders through an anti-inflammatory mechanism. Psychoneuroendocrinology. 2013;38(10):2010-2022. doi: 10.1016/j.psyneuen.2013.03.020. Epub 2013 Apr 23. PMID: 23623252.**	**Depression**
193	Chung SD, Lai TY, Chien CT, Yu HJ. Activating Nrf-2 signaling depresses unilateral ureteral obstruction-evoked mitochondrial stress-related autophagy, apoptosis and pyroptosis in kidney. PLoS One. 2012;7(10):e47299. doi: 10.1371/journal.pone.0047299. Epub 2012 Oct 10. PMID: 23071780; PMCID: PMC3468574.	*Off-target*
194	Lee S-Y, Lee S-J, Han C, Patkar AA, Masand PS, Pae C-U. Oxidative/nitrosative stress and antidepressants: targets for novel antidepressants. Prog Neuropsychopharmacol Biol Psychiatry. 2013;46:224-235. doi: 10.1016/j.pnpbp.2012.09.008. Epub 2012 Sep 26. PMID: 23022673.	Review
195	Manandhar S, Choi B-h, Jung K-A, Ryoo I-g, Song M, Kang SJ, Choi H-G, Kim J-A, Park P-H, Kwak M-K. NRF2 inhibition represses ErbB2 signaling in ovarian carcinoma cells: implications for tumor growth retardation and docetaxel sensitivity. Free Radic Biol Med. 2012;52(9):1773-1785. doi: 10.1016/j.freeradbiomed.2012.02.031. Epub 2012 Mar 3. PMID: 22387177.	*Off-target*
196	Maes M, Fišar Z, Medina M, Scapagnini G, Nowak G, Berk M. New drug targets in depression: inflammatory, cell-mediated immune, oxidative and nitrosative stress, mitochondrial, antioxidant, and neuroprogressive pathways. And new drug candidates–Nrf2 activators and GSK-3 inhibitors. Inflammopharmacology. 2012;20(3):127-150. doi: 10.1007/s10787-011-0111-7. Epub 2012 Jan 24. PMID: 22271002.	Review
197	Pacchioni AM, Vallone J, Melendez RI, Shih A, Murphy TH, Kalivas PW. Nrf2 gene deletion fails to alter psychostimulant-induced behavior or neurotoxicity. Brain Res. 2007;1127(1):26-35. doi: 10.1016/j.brainres.2006.10.036. Epub 2006 Nov 17. PMID: 17113054; PMCID: PMC1831835.	*Off-target*
198	Baudouin-Cornu P, Labarre J. Regulation of the cadmium stress response through SCF-like ubiquitin ligases: comparison between *Saccharomyces cerevisiae*, *Schizosaccharomyces pombe* and mammalian cells. Biochimie. 2006;88(11):1673-85. doi: 10.1016/j.biochi.2006.03.001. Epub 2006 Mar 23. PMID: 16584827.	*Off-target*
199	Viana CE, Bortolotto VC, Araujo SM, Dahleh MMM, Machado FR, de Souza Pereira A, Moreira de Oliveira BP, Leimann FV, Gonçalves OH, Prigol M, Guerra GP. Lutein-loaded nanoparticles reverse oxidative stress, apoptosis, and autism spectrum disorder-like behaviors induced by prenatal valproic acid exposure in female rats. Neurotoxicology.:2022;S0161-813X(22)00199-1. doi: 10.1016/j.neuro.2022.12.006. Epub ahead of print 2022 Dec 14. PMID: 36528186.	Autism
200	Kostyuk SV, Ershova ES, Martynov AV, Artyushin AV, Porokhovnik LN, Malinovskaya EM, Jestkova EM, Zakharova NV, Kostyuk GP, Izhevskaia VL, Kutsev SI, Veiko NN. In vitro analysis of biological activity of circulating cell-free DNA isolated from blood plasma of schizophrenic patients and healthy controls-Part 2: Adaptive response. Genes (Basel). 2022;13(12):2283. doi: 10.3390/genes13122283. PMID: 36553550.	SZ
**201**	**Si L, Xiao L, Xie Y, Xu H, Yuan G, Xu W, Wang G. Social isolation after chronic unpredictable mild stress perpetuates depressive-like behaviors, memory deficits and social withdrawal via inhibiting ERK/KEAP1/NRF2 signaling. J Affect Disord. 2022:S0165-0327(22)01452-5. doi: 10.1016/j.jad.2022.12.092. Epub ahead of print 2022 Dec 27. PMID: 36584714.**	**Depression**
202	Moghaddam AH, Eslami A, Jelodar SK, Ranjbar M, Hasantabar V. Preventive effect of quercetin-Loaded nanophytosome against autistic-like damage in maternal separation model: the possible role of Caspase-3, Bax/Bcl-2 and Nrf2. Behav Brain Res. 2023;441:114300. doi: 10.1016/j.bbr.2023.114300. Epub ahead of print 2023 Jan 12. PMID: 36642103.	Autism
203	Zhang Q, Zheng S, Shi X, Luo C, Huang W, Lin H, Peng J, Tan W, Wu K. Neurodevelopmental toxicity of organophosphate flame retardant triphenyl phosphate (TPhP) on zebrafish *(Danio rerio)* at different life stages. Environ Int. 2023;172:107745. doi: 10.1016/j.envint.2023.107745. Epub ahead of print Jan 10 2023. PMID: 36657258.	*Off-target*
